# Immunophenotype and proviral landscape of HTLV-1c infection and pulmonary disease

**DOI:** 10.1016/j.ebiom.2026.106403

**Published:** 2026-07-23

**Authors:** Ashley Hirons, Natasha Jansz, Georges Khoury, Paula Ellenberg, Sarah Collins, James Cooney, John Zaunders, Lewis Williams, Kirthi Nandha Nair, Ashley Huey Yiing Yap, Nicholas Hirons, Liana Mackiewicz, Merle Dayton, Le Wang, Daniel Sijmons, Mohammad Radwanur Talukder, Allegra Holloway Vickas, Amy W. Chung, Marcel Doerflinger, Marc Pellegrini, Lloyd Einsiedel, Geoffrey Faulkner, Damian F.J. Purcell

**Affiliations:** a Department of Microbiology and Immunology at the Peter Doherty Institute for Infection and Immunity, University of Melbourne, Victoria, Australia; b Mater Research Institute - University of Queensland, TRI Building, Woolloongabba, Queensland, Australia; c Walter and Eliza Hall Institute, Melbourne, Victoria, Australia; d Department of Medical Biology, University of Melbourne, Parkville, Victoria, Australia; e NSW State Reference for HIV, Centre for Applied Medical Research, St. Vincent’s Hospital, Sydney, Darlinghurst, New South Wales, Australia; f University of Melbourne, Victoria, Australia; g Department of Medicine, Alice Springs Hospital, Northern Territory, Australia; h Charles Darwin University, Northern Territory, Australia; i Queensland Brain Institute, University of Queensland, Brisbane, Queensland, Australia

**Keywords:** HTLV-1, Defective and chimeric provirus, Bronchiectasis, Lung-homing CD4+ T-cells

## Abstract

**Background:**

Human T-lymphotropic virus 1 (HTLV-1) integrates into host DNA, resulting in life-long infection that underlies malignancy, inflammatory disease, and early all-cause mortality. HTLV-1 subtype-C endures as an endemic infection in Central Australia, and is associated with pulmonary disease. The cellular and viral features driving HTLV-1c pathogenesis remain poorly understood.

**Methods:**

We recruited a cohort of 41 First Nations participants from Alice Springs Hospital in Central Australia. We analysed plasma biomarker sVCAM1, and three circulating CD4^+^ T-cell phenotypes by flow cytometry: Lung-homing (T_LH,_ CCR4^+^CC49d^+^Integrinβ7^−^), regulatory T-cells (T_REG,_ CCR4^+^CD49d^−^CD127^−^) and CCR4^−^ (T_CCR4−_). We determined the proviral load by ddPCR. Haplotype resolved proviruses were assembled from 6 donors using single provirus amplification and long-read sequencing (SPA-ONT-seq). Analysis of CD4^+^ T-cell phenotypes and the proviral landscape was also performed in a humanised mouse model of HTLV-1c infection.

**Findings:**

All HTLV-1c+ participants showed expansion of chronically activated T_LH_ cells. These cells were highly infected with HTLV-1c provirus and enriched in the lung tissue of humanised mice, confirming pulmonary trafficking. Despite extensive structural diversity in the proviral landscape, defective proviruses in participants with pulmonary disease preferentially retain virulence factor *hbz*. Defective proviruses were detected in the sputum of HTLV-1c+ participants. We discovered chimeric HTLV-1c:human proviruses containing internalised host DNA segments, and implicated them in modulating host gene expression.

**Interpretation:**

HTLV-1c likely contributes to pulmonary disease through lung-homing of chronically activated CD4^+^ T-cells harbouring defective proviruses that retain *hbz*. CD49d and *hbz* could represent potential therapeutic targets. Single-provirus sequencing revealed previously unrecognised structural complexity, including functional viral-host chimeras, with implications for HTLV-1 pathogenesis.

**Funding:**

This work was supported by 10.13039/501100000925NHMRC, 10.13039/100015471Mater Foundation, 10.13039/100009781ACH4, and Miller Foundation PhD Scholarship.

**Research in context**

**Evidence before this study**

HTLV-1 subtype-C and its associated pulmonary disease, endemic in Central Australia and Oceania, remain under-recognised and under-researched compared to the globally prevalent subtype-A. PubMed literature searches for “HTLV-1 subtype-C” and “HTLV-1c” returned 20 results, while “HTLV-1” encompassing most HTLV-1 global studies, returned 9609 results. While both subtypes can cause adult T-cell leukaemia/lymphoma (ATL), HTLV-1-associated myelopathy (HAM), and pulmonary disease (HAPD), they exhibit distinct pathogenic biases: HTLV-1a infection is predominantly associated with ATL and HAM, whereas HTLV-1c infection shows a notable tendency for HAPD. Subtype-specific investigations are clinically important, as pathogenic mechanisms underlying distinct disease outcomes remains poorly understood.

**Added value of this study**

This study provides a comprehensive characterisation of cellular, viral genetic and biomarker features associated with HTLV-1c infection and pulmonary disease. We characterised populations of chronically activated of CD4^+^ T-cells infected with HTLV-1c in blood and demonstrated their capacity to traffic to the lungs in a humanised mouse model. We developed a single-provirus amplification and long-read sequencing method (SPA-ONT-seq). Using SPA-ONT-seq we identified defective proviruses that retain the viral *hbz* gene associate with pulmonary disease. We detected HTLV-1c:human chimeric proviruses that can modulate host cell gene expression.

**Implications of all the available evidence**

This work highlights CD49d and *hbz* as potential therapeutic targets for HTLV-1c-associated disease and possibly for HTLV-1-associated inflammatory diseases more broadly. Our methodology revealed extensive proviral genomic aberrations, structural complexity, and chimeric architectures that associate with pathology.

## Introduction

Human T-lymphotropic virus 1 (HTLV-1) is a complex Delta Retrovirus that causes lifelong infection in ∼10 million people worldwide.^1^ Infection is associated with increased all-cause mortality,^2^ and can cause debilitating and life-threatening diseases with slow onset, including adult T-cell leukaemia (ATL)^3^ and HTLV-1-associated myelopathy (HAM).^4^ HTLV-1 infection is also associated with severe sequelae, including HTLV-1-associated pulmonary disease (HAPD),^5^ among other inflammatory diseases.^6^ Despite the large global health burden, there are no registered antiviral therapeutics that effectively reduce virus pathogenesis or vaccines that prevent transmission.^7^^,^^8^

HTLV-1 predominantly integrates a copy of its 9 kb genome into CD4^+^ T-cells.^9^ Seven subtypes of HTLV-1 have been identified based on divergence in their long-terminal repeat (LTR) promoters and envelope gene. While the most common HTLV-1 subtype is -A (HTLV-1a),^10^ the genetically divergent subtype-C (HTLV-1c) is endemic in Central Australia^11^ and Oceania,^12^, ^13^, ^14^ and has persisted in these isolated populations for tens of thousands of years.^1^ Central Australia has among the highest documented HTLV-1 prevalence rates worldwide, affecting 60% of adults in some remote communities.^15^, ^16^, ^17^ HTLV-1c-associated clinical entities result in higher hospital admission rates in this region.^16^ Clinical data indicate a higher association between HTLV-1c infection and HAPD mortality rates compared to HTLV-1a.^5^^,^^15^^,^^16^^,^^18^^,^^19^ As with poor outcomes of HTLV-1 infection in other endemic areas,^2^ HAPD-related deaths are predicted by high proviral load (PVL).^20^ Key genetic differences in HTLV-1c occur in the 3′regulatory region of the proviral genome (*pX* region),^21^ encoding virulence factors *tax* and *hbz*. It is not clear whether the disposition of HTLV-1c towards HAPD is attributable to genetic variations between HTLV-1 subtypes, host factors including comorbidities and genetic variation, or a combination of both. However, recent studies in non-human primate and humanised mouse models highlight the critical contribution of HTLV-1 subtypes to infection outcomes.^22^^,^^23^

Here we sought to characterise the immune phenotype and proviral reservoir of HTLV-1c-infected cells from a cohort of participants with HAPD from Central Australia, alongside analysis in a humanised mouse model of HTLV-1c infection. We identified a subset of circulating CD4^+^ T-cells that traffic to the lungs and are expanded and chronically activated in HTLV-1c infection. We employed a long-read sequencing approach to assemble haplotype-resolved, near full-length proviral genomes from key cell reservoirs associated with HAPD pathogenesis. We uncovered extensive structural diversity in natural HTLV-1c infection, which included host-viral chimeric genomes, and large provirus insertions and deletions (indels). We found that HAPD is characterised by structural variants that retain the coding sequence of *hbz*, presenting it as a promising therapeutic antiviral target. These comprehensive high-resolution data of the HTLV-1c provirus structure in cellular reservoirs provide fundamental information on both host and viral factors implicated in HAPD pathogenesis in HTLV-1c infection.

## Methods

### Sample collection

PBMCs and plasma were isolated from whole blood buffy coats using Ficoll–Paque™ (GE Healthcare) density centrifugation method, as per the manufacturer’s instructions. Whole blood + PBS 1X mixture was added to Ficoll–Paque™ Plus and centrifuged to separate into phases. Plasma was aliquoted and stored at −80 °C. PBMCs were washed three times with PBS 1X + 2 mM EDTA (BioRad) pH 8.0. PBMCs were cryopreserved until use.

### HTLV-1 serological testing

HTLV-1c serology screening was performed by the National Reference Laboratory (NRL) in Melbourne, Australia. The presence of HTLV-1 specific antibodies was determined using the Murex HTLVI+II enzyme immunoassay (DiaSorin) and Serodia HTLV-1 particle agglutination assay (Fujirebio), in accordance with manufacturer’s criteria. Samples reactive on these assays were then subject to confirmation Western Blot using HTLV-I/II Blot 2.4 (MP Diagnostics), according to manufacturer’s instructions.

### HTLV-1c humanised mouse model

NOD-*scid* IL2Rgamma^null^ (NSG) mice were maintained in the PC3 (HTLV-1c infected) and PC2 (uninfected) animal facility at WEHI, Melbourne, as previously described.^22^ Individually ventilated cages housed a maximum of 6 mice from same litter and sex. Twenty-four hours old mice pups were sub-lethally irradiated (150 rad) using Cobalt-60 (Co-60) teletherapy machine (Best Theratronics Ltd.). After 2 h, pups were intra-facially injected with 5 × 10^4^ – 1 × 10^5^ CD34^+^ human cord blood stem cells (Lonza) in PBS 1X. Mice were monitored for 16 weeks, at which point they were analysed for reconstitution of a human immune system (HIS). Peripheral blood obtained from submandibular bleeds were analysed for human CD45 frequency by flow cytometry. All mice with CD45 positivity between 20 and 90% were selected for HTLV-1c experimentation, while mice with a frequency falling outside these values were excluded from the study. No other exclusions were applied. Numbers of mice per group were determined by the HIS reconstitution. Reconstituted male and female mice were randomly assigned into experimental groups, to maintain equal sex proportions between groups and avoid possible bias associated with HIS reconstitution levels. As infected and uninfected mice were housed in different containment level facilities, group allocation was known throughout the experiment and analysis.

The first HTLV-1c transmission event into the hu-NSG mouse model was achieved using HTLV-1c-infected primary human patient PBMCs. Cryopreserved PBMCs were thawed and lethally irradiated (77 Gy, Co-60) and 1 × 10^6^ PBMCs were intraperitoneally injected into each hu-NSG mouse. Successful HTLV-1c infection was determined at 2 weeks post-infection (wpi) with ddPCR as described below, by using peripheral blood obtained from a submandibular bleed and extracting gDNA as described below. Uninfected control mice were maintained for equal durations. Hu-NSG mice were euthanised by asphyxiation, and harvested spleens were homogenised and filtered through a 40 μm sieve. HTLV-1c infected splenocytes were cultured ex vivo in RF10 (RPMI media +10% FBS), supplemented with 20 U/ml human recombinant interleukin-2 (Lonza), and then cryopreserved until further hu-NSG mouse infections.

All subsequent HTLV-1c infections of hu-NSG mice used 5 × 10^5^ lethally irradiated (77 Gy, Co-60) HTLV-1c infected splenocytes, through intraperitoneal injection. At the conclusion of the experiment, hu-NSG mice were euthanised by asphyxiation, or earlier if mouse weight loss exceeded 20%. Spleens were homogenised and filtered using a 40 μm strainer, and either immediately analysed using downstream applications, or cryopreserved until further use. Bronchoalveolar Lavage (BAL) was performed by making an incision in the neck to expose the trachea, and inserting an 18G drawing needle into the trachea. 1 mL PBS was used to collect lavage fluid from the lungs, repeated twice for a total of 3 mL BALF.

### CD4^+^ T-cell phenotyping

Cryopreserved PBMCs were thawed and washed with fluorescence activated cell sorting (FACS) buffer (PBS 1X + 2.5% FBS) before being incubated with a cocktail of anti-human antibodies in FACS buffer: anti-CD3 PerCP-Cy5.5 (clone SK7, RRID:AB_400190), anti-CD4 Alexa Fluor700 (clone RPA-T4, RRID:AB_10563215), anti-CD38 PE-Cy7 (clone HB7, RRID:AB_3686047), anti-CD45RA PE-CF594 (clone HI100, RRID:AB_562298), anti-CD127 BV786 (clone HIL-7R-M21, RRID:AB_2738138), anti-HLA-DR BV711 (clone G46-6, RRID:AB_2738378), anti-Integrin-β7 BV605 (clone FIB504, RRID:AB_2738729) (BD Biosciences); anti-CD49d BV510 (clone 9F10, RRID:AB_2563820) (Biolegend); anti-CCR4 PE (clone 205410, RRID:AB_2291261) (R&D Systems) for 30 min at 4 °C. Samples were washed twice: first with FACS buffer and then with PBS 1X, and then stained for live/dead (NIR) (Invitrogen) and incubated at 4 °C for 15 min. Cells were washed with FACS buffer and filtered using a 70 μM sieve. Single colour controls were prepared using OneComp eBeads™ (Invitrogen), and live/dead compensation controls were prepared using ArC™ Amine Reactive Compensation beads (Invitrogen). Samples were processed using FACS on Aria III or Aria Fusion, or acquired on Fortessa (BD Biosciences). Sorted CD4^+^ T-cell populations were collected in RPMI media (Gibco) for further immediate processing. Data was acquired using FACSDiva™ software v9.0 (BD Biosciences) and processed using FlowJo v10.8.0 software (FlowJo).

### Genomic DNA extraction

Genomic DNA (gDNA) extraction was carried out with the GenElute™ blood genomic DNA kit (Sigma–Aldrich) according to the manufacturer’s recommendations, with sorted CD4^+^ T-cells, human PBMCs and hu-NSG mice splenocytes. For sorted cell populations with low cell recovery, UltraPure™ Herring Sperm DNA (Invitrogen) was added to the solution as carrier DNA to increase total DNA yields, prior to column binding by centrifugation. gDNA extraction from sputum was carried out by first liquefying the samples with Remel™ Sputasol kit (Thermo Fisher Scientific) according to the manufacturer’s instructions, before proceeding with the GenElute™ blood genomic DNA kit. Eluted gDNA was centrifuged and the supernatant was transferred to a fresh tube, to remove any silica fines. When required, gDNA was concentrated by ethanol precipitation. gDNA concentration and A260/280 ratio was determined using the Nanodrop™ 2000 Spectrophotometer (Thermo Fisher Scientific), according to the manufacturer’s instructions. Final gDNA products were stored at −20 °C until further use.

### Droplet digital PCR (ddPCR)

Droplet digital PCR (ddPCR) primers and probes were designed using selection criteria stipulated by BioRad for HTLV-1c *env* and *hbz* regions (Supplementary Table S1). Primers and probes were blasted to ensure no non-specific binding with other sequences of human, mouse or herring fish genome origins. *Env* and *hbz* primers were further validated by PCR using positive control HTLV-1c+ gDNA. Amplification of 25 ng gDNA was prepared with 1.25 U of GoTaq® Hot Start Polymerase (Promega, Madison, Wisconsin, USA) in a 50 μL reaction mixture with final concentrations of 1X Green GoTaq® Flexi Buffer (Promega), 1.5 mM MgCl_2_ (Promega), 0.2 mM dNTPs (Promega), 0.1 μM forward and reverse primers. Mastercycler Nexus (Eppendorf) thermocycler was used for the following PCR protocol: enzymatic activation for 2 min at 95 °C, then 30 cycles of (denaturation for 30 s at 95 °C, annealing for 1 min at 58 °C, extension for 1 min at 72 °C), followed by a final extension of 5 min at 72 °C. PCR products (5 μL) were electrophoresed in a 2% agarose gel with GelRed 1X (Biotium) in TAE buffer 1X at 100 V. Bands were visualised under UV light and correct amplicon sizes validated. Primers and probes for *gag* and *tax* were previously designed and validated for this assay.^24^

Each ddPCR assay run contained non-template controls, negative and positive HTLV-1c gDNA controls, alongside the HTLV-1c+ gDNA samples. Where possible, each sample was run in duplicate or triplicate. ddPCR assay was prepared and run according to manufacturer’s instructions (BioRad). ddPCR reaction mixture was prepared for gDNA (50–100 ng), combining HTLV-1c forward and reverse (900 nM) primers, HTLV-1c-specific probe (250 nm) (Supplementary Table S1), 2X RPP30 CNV assay mix (BioRad) containing forward and reverse primers and probe, and 2X Supermix for probes (no dUTP) (BioRad). PCR preparation and droplet generator oil for probes (BioRad) were emulsified creating 20,000 droplets per well through the QX-200 droplet generator. PCR was performed with C1000 Touch™ thermocycler (BioRad) under the following conditions: 95 °C for 10 min, 40 cycles of (94 °C for 30 s, 58 °C for 1 min) followed by 98 °C for 10 min, with infinite hold at 10 °C. Cycles had a ramp rate of 2 °C/s, lid heat was 105 °C and sample volume was set to 46 ul. Droplets were then analysed for two colour detection of FAM and HEX using the QX200 droplet reader (BioRad). QuantaSoft v1.7.4 (BioRad) was used to acquire and analyse the fluorescence of the droplets in the FAM and HEX channels. Manual negative thresholds were set for the fluorescence amplitude of each primer/probe target, based on the respective NTC and HTLV-1c negative control wells. The proviral load (PVL) was determined from the number of copies of the HTLV-1c target gene region and the reference gene RPP30, per μL of reaction.

### Bead-based immunoassay

Soluble VCAM-1 (sVCAM-1) concentration in patient plasma was measured using Procartaplex Human Simplex assay (ThermoFisher), according to manufacturer’s instructions. Briefly, plasma stored at −80 °C was thawed and diluted 1:100 in Universal Assay Buffer, supplied by the manufacturer, with each sample prepared in duplicate. Standards and background wells were prepared in duplicate. The capture bead mix (1X) was added to each well, and beads were washed with 1X wash buffer (ThermoFisher) using a magnetic plate washer. Standards and samples were added to the plate and incubated for 2 h at room temperature, shaking at 600 rpm. The plate was washed, biotinylated detection antibody mix added, and incubated for 30 min at room temperature, shaking at 600 rpm. Plate was washed again, and Streptavidin-PE solution was added to each well and incubated for 30 min, shaking at 600 rpm. Plate was washed, reading buffer added to each well and incubated for 5 min at room temperature, shaking at 600 rpm. Plate was run on xMAP INTELLIFLEX (Luminex), and data analysis was conducted using ThermoFisher Procartaplex Analysis App software.

### Single provirus amplification (SPA) assay

Single provirus amplification (SPA) assay is a limiting dilution touchdown nested PCR, designed to amplify single copies of integrated HTLV-1c provirus, modelled on a HIV-1 proviral sequencing study with a similar approach.^25^ The SPA assay uses limiting dilution such that less than 30% of wells contain a copy of proviral amplification, which by Poisson distribution indicates that there is more than 80% probability that this amplification comes from a single integrated provirus. HTLV-1c provirus primers were designed with Primer3Plus web-interface program (version 3.2.6)^26^ and SnapGene to determine the sequences with optimal length, GC content, annealing temperatures, while avoiding hairpin structures or 3′ complementarity. Given the presence of LTRs at both ends of the provirus, primers had to be staggered to amplify as much of the full-length provirus as possible without unwanted amplification of an LTR section. Moreover, the amplification included more of the 3′LTR and less of the 5′LTR, given there have been many reports of 5′-deletions found in HTLV-1a infections, particularly ATL cases.^27^^,^^28^ In addition, we assessed specificity in silico to ensure that primers wouldn’t amplify endogenous retroviral LTRs, and confirmed with control PCRs on HTLV-1c− gDNA.

Both rounds of PCR reaction mixture were 20 μl with final concentrations of 0.5 U of Platinum Taq DNA Polymerase High Fidelity (Invitrogen), 1 μM of both forward and reverse primers (Supplementary Table S1), 2 mM MgSO_4_ (Invitrogen), 0.2 mM dNTPs in 1X high fidelity (HI-FI) buffer (Invitrogen). The first PCR round with 1 μl of limiting dilution gDNA, from sorted CD4^+^ T-cell subsets, bulk PBMCs and splenocytes, used a touchdown protocol on the Mastercycler Nexus (Eppendorf) thermocycler as follows: enzymatic activation for 2 min at 94 °C, then three cycles of each annealing temperature (denaturation for 30 s at 94 °C, annealing for 30 s at 64/61/58 °C, extension for 10 min at 68 °C) followed by 21 cycles of (denaturation for 30 s at 94 °C, annealing for 30 s at 55 °C, extension for 10 min at 68 °C), a final extension at 68 °C for 10 min, finishing with an infinite hold at 10 °C. The nested PCR round used 1 μl of PCR product from the first round as the template and applied the same PCR touchdown thermocycler protocol as the first round, but instead with 31 cycles of annealing temperature 55 °C before the final extension time.

PCR products (1 μl) in Gel loading dye 1X (NEB) were electrophoresed in a 0.8% agarose gel with GelRed 1X (Biotium) in TAE buffer 1X at 100 V, for visualisation of positive wells with a UV transilluminator. Positive wells for SPA were selected and all remaining PCR product was electrophoresed as above, and provirus bands were excised under blue light. DNA was purified using NucleoSpin® Gel and PCR Clean-up kit as per manufacturers’ instructions (Macherey–Nagel).

### Long-read provirus sequencing

DNA libraries were prepared from low-input samples using the Ligation sequencing kit with native barcoding expansion pack (SQK-LSK109 with EXP-NBD196) or Native barcoding kit (SQK-NBD114.96) and were sequenced on the Oxford Nanopore Technologies (ONT) MinION platform (r9.4.1 or r10.4.1 chemistry). Basecalling, demultiplexing and adaptor trimming were performed using Guppy v1.8.5 or Dorado v0.3.4 with high-accuracy or super-high accuracy models. Reads were subset from the peak of the read length distribution generated with NanoPlot v1.40.0 and filtered for the presence of primer binding sequences. Lamassemble^29^ was used to generate consensus sequences from 1000 reads (coverage permitting) for each amplicon.

Proviral consensus sequences were aligned to the full-length HTLV-1c consensus sequence^21^ with the BLASTn algorithm, or to the amplified sequence (680–8899 bp) with Minimap2 v2.28 (-ax map-ont). Alignments were visualised and quantified in Seqmonk v1.48.0 (Babraham Bioinformatics). Relative representation of each nucleotide in proviral amplicons was quantified by sliding window, using 1 bp windows with step size of 1 bp, within 680–8898 bp of the full-length proviral sequence. Sampling between participants was normalised by scaling to the largest store. Breakpoint analysis was performed on the BLAST (NCBI) alignments, and a sequence logo of 20 bp ± breakpoints in each amplicon was generated using WebLogo.^30^ Defective proviral sequences were identified at the alignment stage. Multiple sequence alignments of consensus sequences were generated using the Clustal Omega Web Services, which were used to generate guide tree and pairwise identity matrix. Amplicons containing large internal deletions were defined as gaps > 100 nucleotides. Inversions were identified as unmappable regions that mapped to the reference sequence following reverse complementation. Chimeric HTLV-1c proviral amplicons that contained internal human genetic sequences were identified as regions that did not map to the HTLV-1c consensus, but which did map to the HS1 human genome assembly. These regions were extracted, identified using the BLAT web tool and visualised on Seqmonk v1.48.0. For sensitivity analyses, 11 subsampled genomes were randomly selected for each donor, and haplotypes were collapsed for analyses based on identical breakpoint junctions in Supplementary Table S2. Open reading frames were annotated by manual inspection using SnapGene software, against the full-length HTLV-1c consensus sequence^21^ as a reference, and masked regions of ≥5 poly-nucleotides, which are a known source of sequencing errors on the ONT platform.^31^ Regions of microhomology were identified using the R package mhscanR.^32^

### Chimeric provirus plasmid construction

An artificially synthesised DNA fragment encompassing the HTLV-1 subtype-A 5′LTR, the chimeric provirus amplified by the SPA assay, and the 3′LTR of the HTLV-1c was introduced into an AseI/EcoRI cassette. pLJM1-EGFP was cleaved at the NdeI/EcoRI sites and was used as backbone for the construction of the chimeric molecular clone. A molar ratio of 3:1 (Vector: Insert) was used for the ligation in 1 μL of T4 DNA ligase (New England Biolabs) in 1 X T4 ligase buffer (New England Biolabs), 10 μL final volume. Ligations were performed at room temperature for 2 h 2 μl of the ligation mix was used to transform 50 μl of NEB STBL Chemically Competent *E. coli*. Cells were rested on ice for 30 min, shocked at 42 °C for 45 s on a heat block, following which cells were incubated on ice for 2 min 450 μL prewarmed SOC medium was added to cells, and the transformed bacteria were incubated at 37 °C for 1 h, at 180 rpm. 50 μL transformation was plated on LB ampicillin plates. Plates were incubated at 37 °C overnight. Colonies were selected the following day and grown overnight in 4 mL Luria Bertani (LB) Broth supplemented with (100 μg/mL) ampicillin. Plasmids were isolated using the Macherey–Nagel NucleoSpin plasmid purification kit following the manufacturer’s instructions. Plasmids were eluted in 50 μL elution buffer (5 mM Tris/HCl; pH 8.5). A restriction digestion was then performed using EcoRI. The molecular clones displaying the expected restriction digestion pattern were verified by Nanopore sequencing.

### Lentivirus production and transduction of HEK293T cells

Lentivirus was prepared by transfecting HEK293T cells (RRID: CVCL_0063) with either pLJM1-EGFP, or pLJM1-HTLV-1c:ATG101, pLJM1-HTLV-1c:H2BC12-HA or pLJM1-HTLV-1c:CHOP-HA, with pVSV-G (Addgene_8454) and psPAX2 (Addgene_12260) plasmids in a 3:2:1 ratio using FuGENE reagent. The viral supernatant was collected 48 h following transfection, filtered through a 0.45 μm filter and added to HEK293T cells to confirm chimeric proviral expression, or Jurkat cells to assess effects of chimeric proviral integration on cellular gene expression, in media containing 4 μg/mL polybrene (Sigma–Aldrich). 24 h later, media was changed and 2 μg/mL puromycin (Sigma–Aldrich) added for selection for at least 2 days.

### RNA extraction

RNA extraction from cell lines was performed using RNeasy Minikit (QIAGEN). Cultured HEK293T cells at 90%–100% confluence were harvested, washed in PBS 1X and RNA extracted as per manufacturers’ instructions. RNA extraction from clinical PBMCs was performed using TRIzol LS (Invitrogen) as per manufacturers’ instructions.

### Reverse transcription of RNA

RNA generated above was quantified using the Nanodrop RNA-40 program. cDNA was generated from 1 μg of total RNA using 50 μM Oligo(dT)15 (Life Technologies) and 50 ng random hexamers with 200 U Superscript III Reverse Transcriptase (Life Technologies) as per manufacturer’s instructions.

### RT-qPCR

1.25 μL of 1/10 diluted cDNA was used in a 10 μL PCR reaction. All assays were performed in triplicate, and standard curves were produced for all assays. Reaction mixes contained 1 X PowerTrack SYBR Green Master Mix (Thermo Fisher Scientific) and 400 nM forward and reverse primers (Supplementary Table S1). qPCR was conducted on the QuantStudio™ 5 Real-Time PCR (Thermo Fisher Scientific) instrument with cycling conditions of 95 °C for 2 min, followed by 40 cycles at 95 °C for 15 s, and 60 °C for 60 s. Cycle thresholds (Ct) were calculated using QuantStudio™ 5 software. Quantification of mRNA expression was calculated using absolute standard curves, generated from qPCR performed on a serial dilution of plasmid DNA containing chimera inserts at known copy numbers. Standard curves were generated by plotting the Ct values against the logarithm of the copy number. Copy numbers of experimental RNAs were calculated by linear regression of the absolute standard curve. Relative mRNA expression levels were calculated with the standard curve method, using *HPRT* mRNA expression as a control for variation in cDNA concentration between samples.

### RNA-sequencing

3 × 10^5^ Jurkat cells transduced with pLJM1-EGFP or pLJM1-HTLV-1c:H2BC12-HA were preserved in 150 μL Zymo DNA/RNA Shield and submitted to Plasmidsaurus for RNA extraction and 90 bp single-end Illumina library preparation, sequencing and analysis. Briefly, poly-A mRNA capture was followed by reverse transcription using an oligo-dT primer incorporating a sample barcode and UMI, then second-strand synthesis, tagmentation, and library amplification to append i5, i7, and P5/P7 sequences for sequencing on the Illumina NovaSeq X Plus. Raw reads were filtered using FastP v0.24.0, for poly-A tail trimming, 3′ quality-based trimming, a minimum Phred score of 15, and a minimum read length of 50 bp. Filtered reads were aligned to the hg38 consensus genome with STAR v2.7, excluding non-canonical splice junctions. PCR and optical duplicates were using UMICollapse v1.1.0. Gene expression was quantified with featureCounts (subread v2.1.1) and differential expression was performed using edgePython v0.2.5.

### Preparation of whole cell extract

3 × 10^6^ HEK293T cells were treated with trypsin–EDTA and washed twice in cold MTPBS. Cells were lysed with 500 μL KALB lysis buffer [150 mM NaCl, 50 mM Tris–HCl (pH 7.5), 1% (vol/vol) Triton X-100, 1 mM EDTA (pH 7.5)] supplemented with 1 X cOmplete protease inhibitor (Roche) with rotation at 4 °C for 10 min, followed by centrifugation at 13,000 rpm at 4 °C for 5 min. Supernatant was collected as whole cell extract.

### Western blot

Proteins were resolved by reducing SDS-PAGE on 4%–12% Bis-Tris gels (Life Technologies) in MES buffer (Life Technologies), and transferred to a PVDF membrane sing the iBlot 2 Dry Blotting System (Thermo Fisher Scientific) according to the manufacturer’s instructions. Membranes were blocked in 5% (w/v) skim milk powder in 0.1% Tween-20/PBS overnight at room temperature. The membrane was probed with a HA-Tag (C29F4) Rabbit mAb antibody (Cell Signalling, 3724 T) diluted 1:2000 in blocking solution overnight at 4 °C or for 1 h at room temperature, washed over 30 min with 0.1% Tween-20/MTPBS (6 changes), incubated with Goat anti-Rabbit IgG secondary antibody conjugated to HRP (Invitrogen, 65–6120) diluted 1:2000 in blocking solution for 1 h, and rinsed as above. Antibody binding was visualised using ECL substrate (Thermo Fisher Scientific) according to manufacturer’s instructions, and imaged on the Amersham Imager 800 (Cytiva). Following visualisation, the membrane was incubated in stripping buffer (0.2 M Glycine, 0.1% SDS, 0.05% Tween-20, adjusted to pH 2.2 with HCl) for 5 min twice, followed by two washes in PBS for 10 min each, followed by two washed in 0.1% Tween-20/PBS for 5 min. The stripped membrane was then blocked in 5% (w/v) skim milk powder in 0.1% Tween-20/PBS overnight at room temperature, and probed with an HRP conjugated antibody against beta Actin (Invitrogen, 8A3R) diluted 1:500 in blocking solution for 1 h, and rinsed and visualised as above.

### Statistics

All calculations of means, medians, inter-quartile ranges (IQRs) and univariate and bi-variate statistical analyses were performed using Graphpad PRISM software v9.0. When required, PVL data was log_10_(x) transformed. Clinical data was assumed to have non-parametric distribution, while humanised mouse data was assumed to have parametric distribution. Non-parametric statistical hypothesis testing between two groups was performed using the Mann–Whitney test. Non-parametric statistical hypothesis testing between three or more groups for continuous variables was performed using the Kruskal–Wallis test. Non-parametric statistical hypothesis testing between two groups for continuous variables with matched measurements was performed by using the Wilcoxon matched-pairs signed rank test. Non-parametric statistical hypothesis testing between three or more groups for continuous variables with matched measurements was performed by using the Friedman test. Parametric statistical hypothesis testing between two groups for continuous variables was performed using unpaired t-test with single pooled variance. Parametric statistical hypothesis testing between two groups with matching data for continuous variables was performed by using the paired t test, and between three groups by RM one-way ANOVA with single pooled variance. When required by these non-parametric and parametric statistical tests, false discovery rate (FDR) control for multiple comparisons used two-stage linear step-up procedure of Benjamini, Krieger and Yekutieli, to adjust p values (q values). Statistical contingency tests of categorical variables between three groups were performed using Fisher’s exact test. Correlation analysis between two variables was performed using Spearman test for non-parametric data and Pearson test for parametric data. The Kolmogorov–Smirnov test was used to compare breakpoint frequency distributions between HAPD+ and HAPD− groups. Genomic distributions of the chimeric proviral intervals were analysed with a permutation test, by comparing observed counts per chromosome to a null distribution generated through 10,000 permutations p values were considered significant when ∗p < 0.05, ∗∗p < 0.01, ∗∗∗p < 0.001, ∗∗∗∗p < 0.0001. Data was pre-processed for multivariate analysis: all dataset input variables underwent Z-score transformation prior to analysis. To preserve sample variance, missing values were imputed with average following transformation. PVL was log10 transformed to mitigate the impact of outliers while preserving all samples. Partial least squares discriminant analyses (PLS-DA) were generated using scikit-learn 1.6.1. PLS-DA was conducted using the PLSRegression class in scikit-learn with 2 components. Latent dimensions were oriented in the direction of positive disease status and ordered based on the amount of explained input variance. For visualisation, loadings were scaled to have unit max norm.

### Ethics

Participants were recruited at Alice Springs Hospital, Mparntwe, Northern Territory, as part of an HTLV-1c cohort, with fully informed consent in primary language and full written consent, in accordance with the National Health and Medical Research Council (NHRMC), Central Australian Human Research Ethics Committee (CAHREC) and Declaration of Helsinki Ethical Principles. Participant numbers were determined by the number of consents obtained over the two-year collection period 2018–2019. This study is approved by CAHREC under the ethics reference HREC-17-2930, and The University of Melbourne Human Research Ethics Committee (1442830.1). Other negative control buffy blood samples were obtained from the Lifeblood Red Cross, Naarm, Melbourne, under the research agreement 17-08VIC-01.

All work involving the humanised (hu) mouse model was approved by the WEHI Animal Ethics Committee (2017.016) and adhered to the Animal Research: Reporting of In Vivo Experiments (ARRIVE) guidelines.

### Role of funders

Funders for the project and for investigators did not have any role in study design, data collection, data analysis, interpretation or writing of the report.

## Results

### HTLV-1c associated pulmonary disease is sustained by chronically activated and infected lung-homing CD4^+^ T-cells

Identifying the phenotype of HTLV-1-infected CD4^+^ T-cells is important for understanding key proviral reservoirs implicated in disease, and harnessing that knowledge for developing therapeutic strategies that reduce proviral burden. CCR4^+^CD4^+^ T-cells have been identified as an HTLV-1a provirus reservoir.^33^^,^^34^ Given the clinical significance of HAPD in subtype-C infection in Central Australia, we first characterised CD4^+^ T-cells associated with lung homing (T_LH_, CCR4^+^CD49d^+^Integrinβ7^−^). Given that CCR4^+^CD49d^−^ T-cells were reported to preferentially migrate to the skin, and CD49d^+^Integrinβ7^+^ cells reported to preferentially migrate to the gut,^35^^,^^36^ we reasoned that the CCR4^+^CD49d^+^Integrinβ7^−^ phenotype likely represents cells that traffic to the respiratory tract.^37^^,^^38^ We also measured chronic activation markers HLA-DR^+^CD38^+^ in this reservoir, which is important in the Central Australian setting where frequent comorbidities and coinfections exist.

PBMCs from an HTLV-1c cohort, comprising infected (n = 27) and uninfected (n = 14) First Nations donors from Mparntwe (Alice Springs), Central Australia, were stained using a 10-colour panel and processed via flow cytometry (Supplementary Fig. S1). Demographic and clinical details of the HTLV-1c cohort are detailed in Table 1, where the HTLV-1c+ group was further stratified by HAPD. Chronic kidney disease^39^ (p = 0.008), blood stream infection^16^ (current or previous) (p = 0.006), strongyloidiasis^16^ (current) (p = 0.049) – known associations with HTLV-1 infection in Central Australia, were significantly different between the cohort groups. Hospital-based matched controls account for the higher rates of comorbidities and reduced life expectancy in First Nations people in Central Australia due to the ongoing impacts of colonisation, when compared to non-Indigenous individuals. The PVL of HTLV-1c+ individuals, measured by ddPCR using PBMC-derived gDNA where yield was sufficient, was not different between HAPD− (n = 10, median = 2.77 × 10^3^ copies/PBMCs) and HAPD+ (n = 13, 2.28 × 10^3^ copies/PBMCs) groups (p = 0.94) (Table 1).

**Table 1:** Demographics and clinical characteristics of HTLV-1 cohort from Central Australia.

	HTLV-1c− (n = 14)	HTLV-1c+ HAPD− (n = 12)	HTLV-1c+ HAPD+ (n = 15)	p value
Demographics, n (%)				
Age (median)	48.5	57.5	55.0	0.10
Female AAB	42.9% (6/14)	75.0% (9/12)	40.0% (6/15)	0.17
Male AAB	57.1% (8/14)	25.0% (3/12)	60.0% (9/15)	
Lifestyle, n (%)				
Smoking (current or previous)	78.6% (11/14)	25.0% (3/12)	40.0% (6/15)	**0.02**
Alcohol consumption	66.6% (8/12^b^)	50.0% (6/12)	33.3% (4/12)	0.44
Clinical conditions, n (%)				
CLD	0% (0/14)	16.7% (2/12)	0% (0/15)	0.08
Diabetes	57.1% (8/14)	83.3% (10/12)	60.0% (9/15)	0.30
CKD	21.4% (3/14)	83.3% (10/12)	46.7% (7/15)	**0.008**
BSI (current or previous)	0% (0/14)	50.0% (6/12)	40.0% (6/15)	**0.006**
Strongyloidiasis (current)	0% (0/14)	25.0% (3/12)	33.3% (5/15)	**0.049**
Pulmonary disease^a^	6.7% (1/14)	0% (0/12)	100% (15/15)	N/A^c^
Malignancy	0% (0/14)	16.7% (2/12)	13.3% (2/15)	0.43
HTLV-1c burden, copies per 10^6^ PBMCs				
PVL (median)	N/A	2.77 × 10^3^ (n = 10^b^)	2.28 × 10^3^ (n = 13^b^)	0.94

41 people were recruited for the HTLV-1c pathogenesis study at Alice Springs Hospital, Mparntwe, Central Australia, with written and verbal consent in English and primary language. This cohort comprised three subgroups: HTLV-1c− (n = 14), HTLV-1c+ HTLV-1c associated pulmonary disease negative (HAPD−) (n = 12) and HTLV-1c+ HAPD+ (n = 15). Participant information collected included age, sex assigned at birth, lifestyle, clinical conditions and HTLV-1c proviral load, determined by droplet digital PCR.

AAB, assigned at birth; CLD, chronic liver disease; CKD, chronic kidney disease; BSI, blood stream infection; ATL, adult T cell leukemia/lymphoma; HAM, HTLV-1 associated myelopathy; PVL, proviral load; HAPD, HTLV-1 associated pulmonary disease.

Bold indicates statistical significance where p < 0.05.

a Pulmonary disease includes bronchiectasis, bronchiolitis and chronic obstructive pulmonary disease.

b Indicates missing data. Statistical significance between three groups for continuous variables was assessed using Kruskal–Wallis test and for categorical variables using Fisher’s exact test. Statistical significance of PVL between HTLV-1c+ groups was assessed with Mann Whitney test.

c Statistical testing not applicable as groups are stratified by pulmonary disease.

**Fig. 1:**
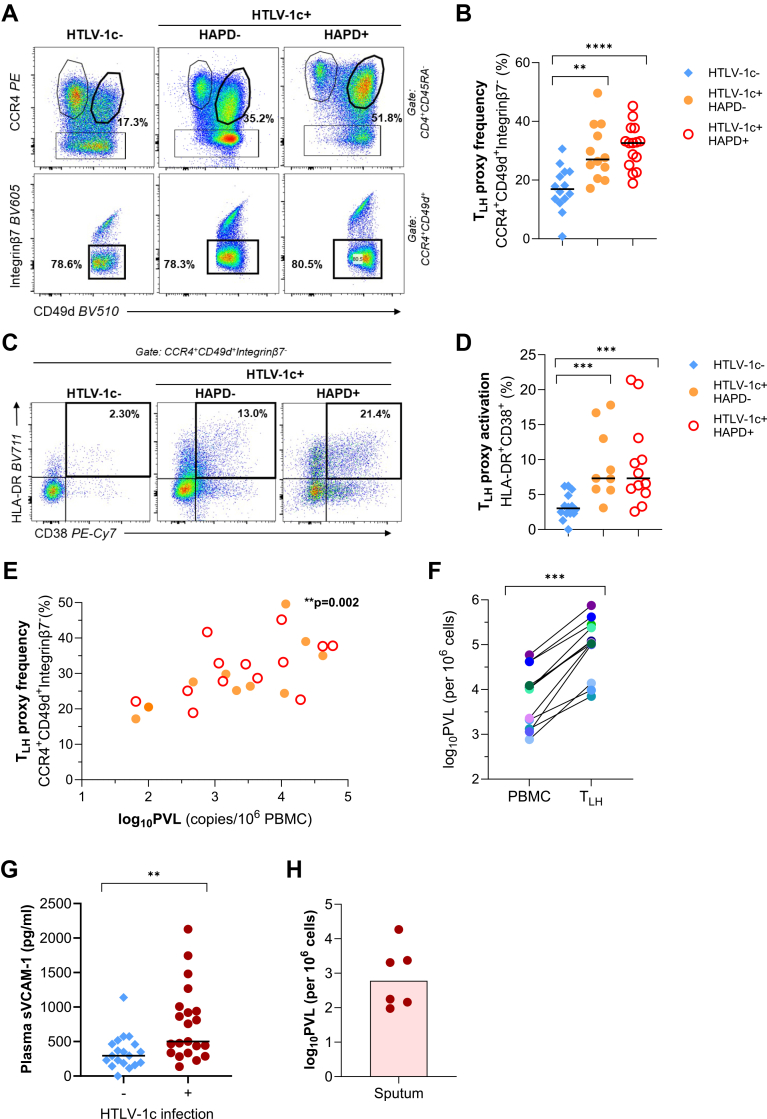
**HTLV-1c infection is associated with expansion and activation of lung homing proxy CD4^+^ T-cells containing high levels of provirus.** (A) Flow cytometry plots of CCR4 (PE) and Integrin-β7 (BV605) with CD49d (BV510), showing expansion of CCR4^+^CD49d^+^Integrinβ7^−^ population for HTLV-1c+ individuals. (B) Frequency of lung homing proxy (T_LH_) cells as a percentage of CD4^+^CD45RA^−^ population in 41 participants (HTLV-1c− n = 14; HTLV-1c+ HAPD− n = 12; HTLV-1c+ HAPD+ n = 15). Median indicated by black line. (C) Flow cytometry plots of HLA-DR (BV711) and CD38 (PE-Cy7) showing increased chronic activation of T_LH_ cells (CCR4^+^CD49d^+^Integrinβ7^−^) in HTLV-1c+ individuals. (D) Frequency of chronic activation marker expression (HLA-DR^+^CD38^+^) in T_LH_ reservoir. Median indicated by black line. (E) Correlation between T_LH_ frequency and log_10_PVL (copies/10^6^ PBMC) of HTLV-1c+ participants. (F) log_10_PVL of T_LH_ and PBMC reservoirs in 12 HTLV-1c+ participants, each colour represents different donor. (G) Concentration of soluble VCAM-1 in plasma from 40 participants (HTLV-1c− n = 19, HTLV-1c+ n = 21). Median indicated by black line. (H) log_10_PVL of sputum in 6 HTLV-1c+ participants. Statistical significance in (B) and (D) assessed by Kruskal–Wallis test with false discovery rate (FDR) adjustment, in (E) Spearman test, in (F) Wilcoxon matched-pairs signed rank test, in (G) Mann–Whitney test, ∗p < 0.05, ∗∗p < 0.01, ∗∗∗p < 0.001, ∗∗∗∗p < 0.0001.

We measured the frequency of T_LH_ CD4^+^ T-cell proxy phenotype in clinical samples to identify HTLV-1c-associated phenotypic changes in peripheral blood that could mediate pulmonary disease. There was a significant expansion of T_LH_ cells in both HAPD− (median = 27.00%, p = 0.001) and HAPD+ (32.60%, p < 0.0001) HTLV-1c+ groups when compared to negative controls (16.95%) (Fig. 1A and B). Furthermore, T_LH_ cells in HTLV-1c+ donors displayed significantly higher chronic activation marker expression (HLA-DR^+^CD38^+^) than controls (HAPD− p = 0.0007, HAPD+ p = 0.0007) (Fig. 1C and D). Interestingly, the frequency of T_LH_ cells was positively correlated to the PVL in PBMCs (p = 0.01) (Fig. 1E), indicating that infection is driving expansion of this reservoir. To this end, we assessed HTLV-1c provirus enrichment in this population by quantifying the PVL in paired T_LH_ phenotype and PBMC samples in 12 HTLV-1c+ participants by ddPCR, where we had sufficient bulk gDNA from these matched populations. The T_LH_ reservoir had ten-fold higher PVL (median = 1.14 × 10^5^ copies/10^6^ cells; p = 0.0005) relative to PBMCs (1.04 × 10^4^) (Fig. 1F), which may reflect near-universal infection or multiple copies of provirus per cell, as observed in ATL associated with HTLV-1a, and should be addressed in future studies.

We wanted to confirm whether these chronically activated, HTLV-1c-infected, circulating T_LH_ cells could migrate to the lung. As bronchoalveolar lavage fluid (BALF) and lung tissue biopsies were not available in the Central Australian clinical setting, we utilised multiple complementary approaches to address this question: a humanised mouse model to directly assess lung trafficking, and indirect clinical markers of pulmonary involvement in human samples. CD34^+^ stem cell engrafted humanised NOD-scid *Il2rg*^null^ (hu-NSG) mice swiftly establish HTLV-1c infection when injected with lethally irradiated HTLV-1c+ cells, reaching 100% PVL by 4 weeks as confirmed by ddPCR in peripheral blood.^22^ Using this humanised mouse model, we have shown HTLV-1c-induced CD3^+^ T-cell migration into the lungs and lung histopathology.^22^ Expanding on this work with our current study, we harvested splenocytes of 13 hu-NSG mice at 2 weeks post-infection (wpi) (HTLV-1c+ n = 8; uninfected n = 7) when the PVL more closely resembled human infection (mean = 3.92 × 10^4^ copies/10^6^ splenocytes) and analysed samples by flow cytometry with the same 10-colour panel of anti-human antibodies as the clinical cohort study. We further harvested hu-NSG samples from blood and lung at 6 wpi (HTLV-1c+ n = 3), when the mice present with high grade infection, for analysis by flow cytometry.

We confirmed that frequency of T_LH_ phenotype was significantly expanded in HTLV-1c+ mice (mean = 33.75%, p = 0.02) compared to controls (15.74%) in splenocytes at 2 wpi (Supplementary Fig. S2A and B). In addition, these cells in HTLV-1c+ mice displayed over three times the frequency of chronic activation marker expression than uninfected mice (p < 0.0001) (Supplementary Fig. S2C and D). Furthermore, we showed T_LH_ splenocyte cells are more highly infected with HTLV-1c provirus (mean = 9.40 × 10^4^ copies/10^6^ cells; p = 0.01), compared to paired bulk splenocytes (2.41 × 10^4^) (Supplementary Fig. S2E) (n = 5). Finally, we determined that T_LH_ phenotype is indeed present at significantly higher levels in BALF compared to paired circulating PBMCs (p = 0.03) of HTLV-1c+ hu-NSG mice at 6 wpi (n = 3) (Supplementary Fig. S2F and G). This direct demonstration of T_LH_ enrichment in the lungs validates the phenotypic marker as indicative of lung-homing capacity and provides evidence that HTLV-1c-infected T_LH_ cells traffic to pulmonary tissue in vivo.

To complement these experimental findings with further clinical observations, we examined two indirect markers of lung trafficking in human samples: soluble vascular cell adhesion molecule-1 (sVCAM-1) plasma levels and proviral DNA in sputum. Transmigration from blood to tissues is mediated by the expression of very late antigen-4 (VLA-4) and its constituent CD49d on the surface of T-cells, and interaction with VCAM-1 on endothelial cells.^40^^,^^41^ High levels of chronic T-cell activation-induced inflammation at tissue barriers trigger shedding of VCAM-1 from endothelial cells.^41^ Therefore, we measured the plasma concentration of sVCAM-1 in 40 samples from Central Australia (HTLV-1c− n = 19, HTLV-1c+ n = 21) coinciding with upregulation of CD49d expression on chronically activated CD4^+^ T-cells. Indeed, sVCAM-1 was significantly higher in HTLV-1c+ plasma (median = 501.2 pg/mL, p = 0.006) compared to controls (294.6 pg/mL) (Fig. 1G), consistent with inflammation and potential tissue trafficking. We further isolated gDNA from HTLV-1c+ patient sputum samples from Central Australia (n = 6) and detected the presence of provirus (median = 1.11 × 10^3^ copies/10^6^ cells) (Fig. 1H), confirming HTLV-1c-infected cells are present in the respiratory tract.

Together these observations support a model in which migration of chronically activated, HTLV-1c-infected CD4^+^ T-cells to the lungs contributes to pathogenesis, inflammation and pulmonary disease, consistent with well-documented HAPD in this region.

### T_REG_ dysregulation defines HTLV-1c persistence

We next evaluated another cell population identified as an expanded HTLV-1a provirus reservoir in our investigation of subtype-C infection in Central Australia: T_REG_ FOXP3^+^ CD4^+^ T-cells.^42^ We used surface proxy markers for FOXP3^+^ T_REGS_ (CCR4^+^CD49d^−^CD127^−^)^43^ to define this phenotype, enabling downstream long-read sequencing of the provirus, alongside measuring chronic activation with HLA-DR^+^CD38^+^ expression. We also included CCR4^−^ phenotype (T_CCR4−_, CCR4^−^CD49d^+/−^) as a control reservoir, for cells not involved in tissue homing or immune regulation.

When examining the T_REG_ proxy phenotype in peripheral blood samples from Central Australia, the frequency did not differ significantly between HTLV-1c+ HAPD− (median = 8.61%, p = 0.18) or HAPD+ (8.44%, p = 0.20) participants compared to HTLV-1c− controls (5.82%) (Supplementary Fig. S3A), contrasting reports of expanded FOXP3^+^CD4^+^ T-cells in HTLV-1a infection.^42^ T_REG_ proxy cells, however, displayed significantly higher chronic activation in both HTLV-1c+ groups compared to negative controls (p = 0.0006, 0.0006, respectively) (Supplementary Fig. S3B). We measured the PVL by ddPCR and determined T_REG_ phenotype cells were enriched for HTLV-1c provirus (median = 1.08 × 10^5^ copies/10^6^ cells; p = 0.0005) relative to matched PBMCs (1.08 × 10^4^) (n = 8) (Supplementary Fig. S3C).

T_CCR4−_ cell frequencies did not differ between HTLV-1c+ groups (HAPD− median = 21.40%, HAPD+ 19.30%) and controls (29.80%, p = 0.12, 0.05, respectively) (Supplementary Fig. S3A). Analogous to T_LH_ and T_REG_ reservoirs, significantly higher frequencies of chronically activated cells were seen in HTLV-1c+ participants in T_CCR4−_ phenotype when compared to negative controls (p = 0.0003, 0.0003, respectively) (Supplementary Fig. S3B). T_CCR4−_ phenotype cells (5.74 × 10^4^; p = 0.04) also displayed significant enrichment of HTLV-1c provirus compared to matched PBMCs (1.08 × 10^4^) (n = 8) (Supplementary Fig. S3C).

These findings were broadly recapitulated in hu-NSG mice: the frequency of T_REG_ proxy and T_CCR4−_ cells did not display significant HTLV-1c-associated changes (Supplementary Fig. S3D), but were chronically activated compared to negative controls (p = 0.003, 0.0002, respectively) (Supplementary Fig. S3E). T_CCR4−_ cells were significantly enriched for HTLV-1c provirus (mean = 1.17 × 10^5^; p = 0.003) compared to bulk splenocytes (2.41 × 10^4^), however not in T_REG_ proxy phenotype (3.04 × 10^4^) (Supplementary Fig. S3F). These findings are consistent with our previous work in HTLV-1c hu-NSG mice,^22^ and findings in subtype-A HBZ-transgenic mice^44^ and clinical HAM,^45^^,^^46^ indicating that reduced FOXP3 expression contributes to inflammatory phenotypes.

To confirm that sex assigned at birth (AAB) in our cross-sectional clinical cohort was not associated with differences in circulating frequencies, activation levels of CD4^+^ T-cell phenotypes, or PVL, we analysed disaggregated data for both HTLV-1c− and HTLV-1c+ groups, and did not find any significant differences between sex AAB (Supplementary Fig. S4A and B). To broadly analyse differences in CD4^+^ T-cell phenotypes and activation between HTLV-1c− and HTLV-1c+ individuals to confirm our univariate analyses, we performed multivariate Partial Least Squares Discriminant Analysis (PLS-DA) with the dependent variable of HTLV-1c infection status. We controlled for potential confounding clinical variables including age, sex AAB, smoking status (current or previous), BSI (current or previous), and strongyloidiasis (current). We confirmed that HTLV-1c− and HTLV-1c+ donors from Central Australia were separable when considering all CD4^+^ T-cell phenotypes and activation frequencies (Supplementary Fig. S4C and D). We similarly performed a PLS-DA on the HTLV-1c+ group with a dependent variable of HAPD status, however we were not able to show distinct separation between HAPD− and HAPD+ (Supplementary Fig. S4E and F). Therefore, we reasoned that there must be other contributing viral factors that are further associated with pulmonary disease in HTLV-1c infection.

### Single-provirus long-read sequencing reveals structural diversity and DNA-repair breakpoint signatures in the HTLV-1c provirus landscape

To discern molecular features of HTLV-1c pathogenesis and HAPD, we characterised the provirus landscape within circulating PBMCs and the cellular reservoirs phenotyped above. We profiled individual, contiguous, provirus genomes with nucleotide resolution. To this end, we employed a bespoke single provirus amplification (SPA) strategy coupled with long-read sequencing on the Oxford Nanopore Technologies (ONT) platform (SPA-ONT-seq). SPA-ONT-seq advances upon previous genome sequencing strategies used in HTLV-1 studies which involve bulk-genome and short-read sequencing.^21^^,^^28^^,^^47^, ^48^, ^49^, ^50^ The SPA assay uses a limiting dilution, highly-processive, touchdown, nested PCR to amplify and enrich for single integrations of provirus,^25^ (Supplementary Fig. S5A), avoiding amplification of host integration sites to respect First Nations sovereignty over this genomic information. Such an enrichment strategy is necessary to achieve sufficient sequencing depth of single-copy kilobase-scale proviral genomes integrated within eukaryotic host genomes, particularly where the proviral load is low. Validation of this approach by long-read whole genome sequencing in hu-NSG mice confirmed the efficiency of the SPA assay. SPA-ONT-seq enriches for full-length and type 1 defective proviral genomes^28^ possessing both LTRs, but not type 2 genomes that retain a single LTR (Supplementary Fig. S6A), with preferential amplification of shorter genomes (Supplementary Fig. S6B) due to mean DNA fragment lengths <10 kb (Supplementary Fig. S6C). We sequenced proviral amplicons from PBMCs of six HTLV-1c+ participants (HAPD− n = 3, HAPD+ n = 3) and T_LH_, T_REG_ and T_CCR4−_ reservoirs from two of those individuals (HAPD− n = 1, HAPD+ n = 1). We additionally sequenced proviral amplicons from splenocytes of two HTLV-1c+ humanised mice at 6 wpi. At this timepoint the PVL in splenocytes reached ∼100%, providing sufficient genomic material for investigation. A consensus sequence was generated from >1000 reads for each provirus, to correct for random sequencing errors, and aligned to an HTLV-1c Central Australian consensus genome for analysis^21^ (Supplementary Fig. S5B).

**Fig. 2:**
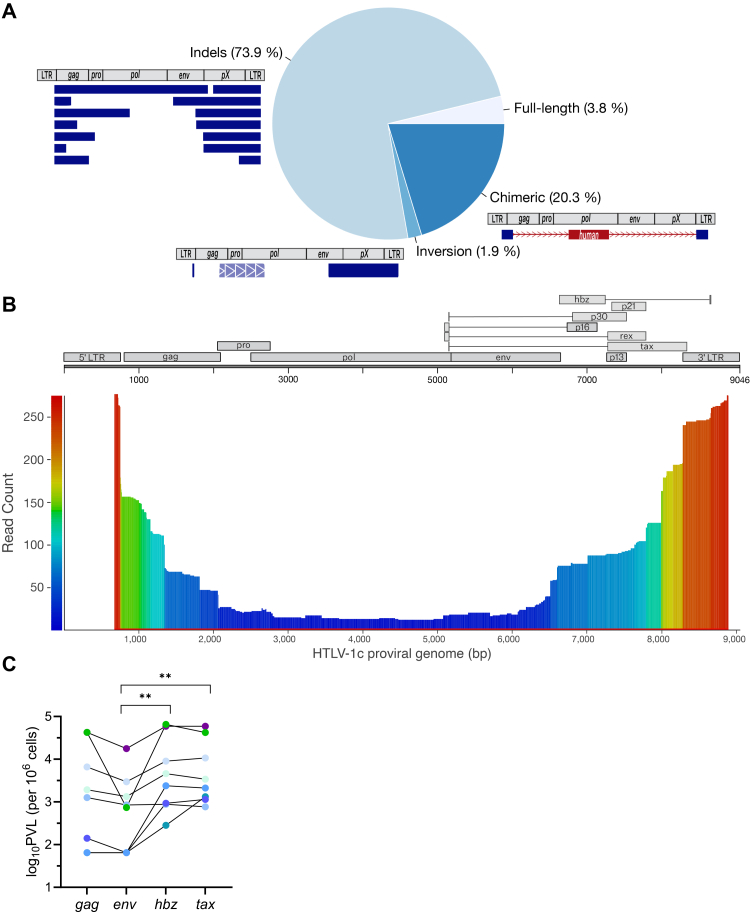
**The proviral landscape in HTLV-1c infection shows deletion of the structural genes.** (A) Distribution of HTLV-1c structural variants of 260 proviral genomes assembled by SPA-ONT-seq from 6 HTLV-1c+ participants from Mparntwe, shown with a schematic representation of each variant. (B) Coverage of each nucleotide in the HTLV-1c proviral genome from 260 individual, contiguous genomes assembled by SPA-ONT-seq from six HTLV-1c+ individuals, aligned to the HTLV-1c consensus sequence (grey). Coverage was quantified at each nucleotide along the consensus, represented as counts along the provirus (hot-cold gradient). (C) log_10_ copies of proviral genes *gag*, *env*, *hbz* and *tax*, per 10^6^ PBMCs, detected by ddPCR in 8 HTLV-1c+ participants from Mparntwe, each colour represents different donor. Statistical significance in (C) assessed using Friedman test with false discovery rate (FDR) adjustment, ∗∗p < 0.01.

In total, 260 individual proviral consensus sequences were assembled from clinical samples with a mean length of 2240 bp (Supplementary Fig. S7A), detailed in Supplementary Table S2. Nucleotide homology to the consensus sequence was between 94 and 100% for all proviruses, indicating that the genome is relatively stable at the single-nucleotide level. Pairwise analysis found that sequences largely clustered by genome retention (Supplementary Fig. S7B and C). Our analyses revealed high structural diversity: 73.9% of the sequenced proviral genomes harboured large internal deletions, 1.9% contained a sequence inversion, while only 3.8% were full-length. Interestingly, 20.3% of proviral sequences were chimeric, such that they possess an internal sequence that mapped to the human genome flanked by HTLV-1c sequences (Fig. 2A). Alignment of individual proviral genomes assembled in this study (excluding chimeras and inversions) to the HTLV-1c consensus genome is presented highlighted by donor as Supplementary Fig. S8. Nucleotide coverage analysis of the proviruses from Central Australia determined the LTRs containing primer binding sites showed the greatest coverage, with counts between 190 and 260. The *gag* gene and *pX* genes were detected with counts between 43 and 147. Meanwhile, structural and enzymatic regions *pro*, *pol* and *env* were rarely retained in the defective proviruses, with counts between 11 and 37 (Fig. 2B). To provide an alternative quantitative validation of the SPA-ONT-seq method in bulk gDNA samples, we designed ddPCR assays across the HTLV-1c genome to assess the presence of *gag*, *env*, *hbz*, and *tax* (Supplementary Table S2), in the PBMCs of 8 HTLV-1c+ participants (P085 and P136, and n = 6 independent donors). As with SPA-ONT-seq, the 3′regulatory gene regions were present at higher levels in proviral DNA than the structural genes. Significantly higher PVLs were observed for *hbz* (median = 3.49 × 10^3^ copies/10^6^ cells; p = 0.004) and *tax* (2.77 × 10^3^; p = 0.008) than *env* (7.92 × 10^2^) (Fig. 2C), corroborating the differential gene retention patterns observed by SPA-ONT-seq.

**Fig. 3:**
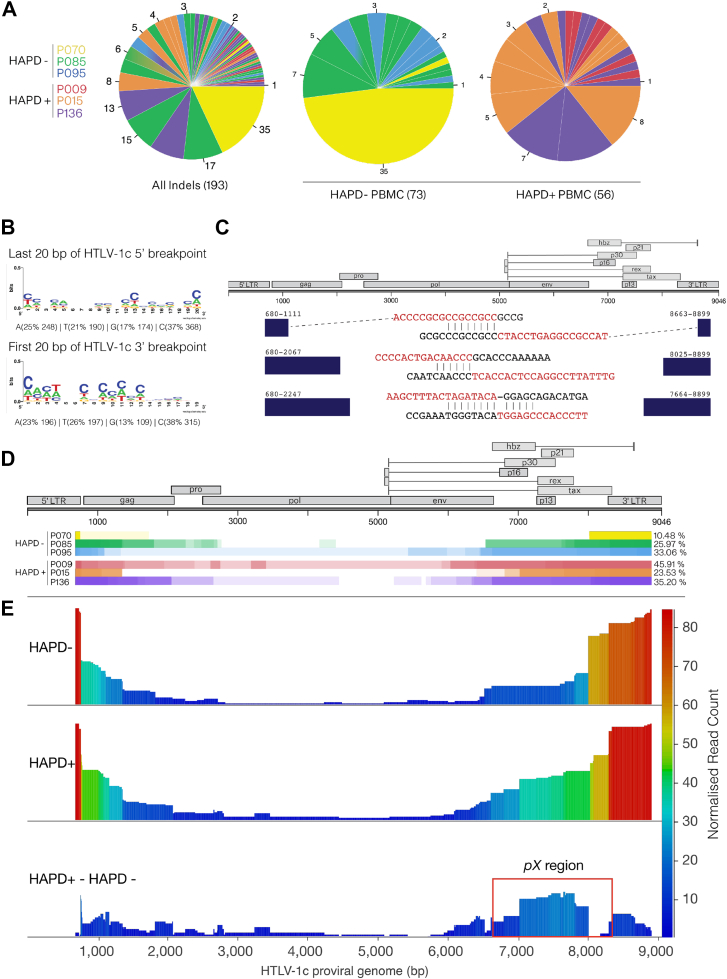
**Molecular features of the HTLV-1c proviral landscape and association with pulmonary disease.** (A) Quantification of unique breakpoints in 193 defective proviral genomes containing a large internal deletion, coloured by donor (n = 6) (left), and in 129 defective genomes amplified from PBMCs stratified by HAPD status (n = 6) (right). Recurrent inter-donor breakpoints are indicated by a colour gradient, coloured by donor (green-purple-blue, and orange-green segments) and did not correspond to HAPD status. The distribution of unique and recurrent breakpoints does not differ between HAPD− and HAPD+ donors (ns, Kolmogorov–Smirnov test). (B) Sequence motif generated from 20 bp ± the breakpoint junction of defective provirus containing a large internal deletion, with the proportion of each nucleotide across breakpoint junctions below. (C) Schematic of representative defective proviral genomes (blue) aligned to the HTLV-1c consensus, with nucleotide resolution of regions of microhomology observed at the breakpoints. Nucleotides from the assembled defective genomes are depicted in red, flanking sequences from the HTLV-1c consensus sequence that show homology are depicted in black, and nucleotide identity is indicated by a line between them. The top defective genome was identified 5 times across 3 independent donors (HAPD−: P085, P095; HAPD+: P136), the middle defective genome was detected 15 times in P136, and the bottom defective genome was detected 2 times in P085. (D) Nucleotide coverage of proviral genomes from PBMCs stratified and coloured by participant. Average coverage shown as a percentage. Coverage is represented by opacity. (E) Normalised coverage of each nucleotide of proviral genomes enriched from PBMCs (hot-cold gradient) stratified by HAPD– (top, n = 3) or HAPD+ (middle, n = 3), and difference (bottom).

We analysed the breakpoint junction in defective proviral genomes to determine the frequency of recurrent genome re-arrangements allowing assessment of dominance metrics and within-donor redundancy of the structural variants assembled by SPA-ONT-seq. We detected 56 unique breakpoints in the 194 defective proviral genomes containing large insertions or deletions (indels) from the clinical samples (Fig. 3A). We detected frequent recurrent intra-donor breakpoint junctions, noting one proviral genome with breakpoints at the 5′LTR and the *tax* coding region was detected 35 times in a single HAPD− donor. An identical provirus sequence detected multiple times could indicate clonal expansion, however we were unable to confirm clonality by sequencing integration sites in this study. We identified two breakpoints that were common across multiple donors occurring between the *gag* gene and the 3′LTR, identified in both HAPD− and HAPD+ donors. However, the distribution of unique and recurrent breakpoints did not differ between HAPD− and HAPD+ donors. We then examined the breakpoint junction sequences ±20 bp, which could inform mechanisms underlying genome deletions, and found that all breakpoints were cytosine rich (Fig. 3B). Notably, when examining individual breakpoints, we frequently observed regions of microhomology between the 5′ and 3′ breakpoints in defective genomes (Fig. 3C). Microhomology scoring was performed on proviruses with a large internal deletion spanning a contiguous region with no insertions, 10 bp ± the breakpoints. Microhomologies of 4–10 bp in length were found at 58 out of 185 (31.3%) of the deletion breakpoint junctions identified (Supplementary Table S3).

In the hu-NSG mice that lack any adaptive immune response to the HTLV-1c infection, 72 proviral sequences were assembled with a mean length of 788 bp (Supplementary Fig. S9A), detailed in Supplementary Table S4. Overall, the proviral landscape of hu-NSG mice detected by SPA-ONT-seq demonstrated similar structural diversity to human infection: 81.9% of proviruses detected contained large internal deletions, and the remaining 18.1% were HTLV-1c:cellular chimeras (Supplementary Fig. S9B). The defective hu-NSG mice provirus sequences similarly contained deletions in the structural and enzymatic genes. However, at this late infection timepoint, the internal deletions detected were more extensive than human infection (Supplementary Fig. S9C). Overall, defective provirus breakpoint characteristics were very similar between mice (Supplementary Fig. S9D). Akin to human infection, we observed regions of microhomology at individual provirus breakpoint junctions (Supplementary Fig. S9E). Taken together, our analyses have revealed high structural diversity in the HTLV-1c proviral landscape, with signs of defective DNA repair pathways.

### Retention of *hbz* gene distinguishes HAPD in HTLV-1c infection

Following in-depth characterisation of the structural diversity in the defective proviral landscape of HTLV-1c infection, we sought to identify genetic features of proviruses that associate with pulmonary disease. We observed a consistent trend for higher genomic coverage in PBMCs isolated from HAPD+ individuals (mean = 33.34%), when compared with HAPD− (mean = 23.00%) (Fig. 3D, Supplementary Fig. S8). The most striking difference of the proviral landscape between groups lay in the 3′regulatory region, which was retained in HAPD+ participants at a markedly higher rate than HAPD−. This region includes the *hbz* gene, which is expressed from an antisense transcript initiating in the 3′LTR (Fig. 3E). Greater 3′ genome retention in HAPD was observed when performing the analysis with equal subsampling per donor (n = 6, 11 genomes per donor) and in analysis of unique haplotype-resolved structural variants (n = 43) to control for pseudoreplication (Supplementary Fig. S10A–D). Furthermore, full-length provirus was only amplified from HAPD+ participants. We sought to confirm that *hbz* RNA expression is retained in a defective provirus landscape. We isolated total RNA from four HTLV-1c+ donors from Central Australia with matched PVLs (2 HAPD+, 2 HAPD-) (Supplementary Fig. S11A) and three HTLV-1c− donors, and performed RT-qPCR for *hbz*, *tax/rex*, *env* and *gag* transcripts. We detected expression of housekeeping gene *HPRT* in 3/3 HTLV-1c− samples, and 3/4 HTLV-1c+ samples, but could only detect expression of *hbz*, and to a lesser extent *tax/rex* in one HAPD+ sample (Supplementary Fig. S11B). Together, these analyses suggest that in a defective HTLV-1c proviral landscape, retention of viral genes, particularly *hbz,* may contribute to HAPD.

### Tissue compartmentalisation of defective and intact HTLV-1c provirus reservoirs

**Fig. 4:**
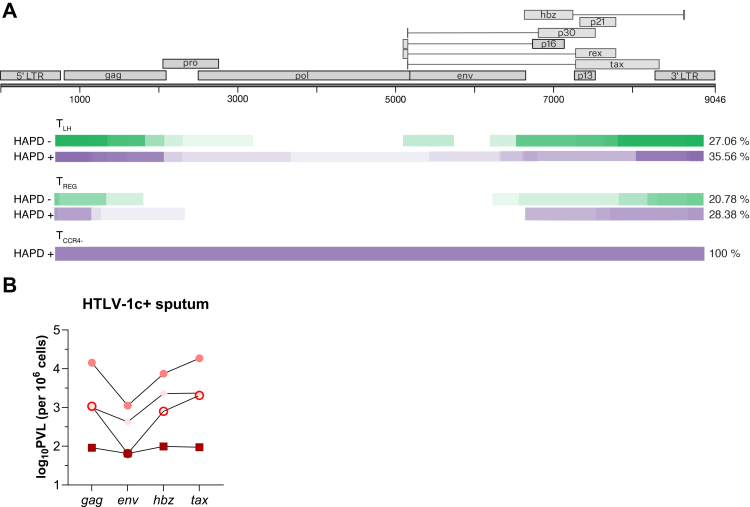
**Cellular reservoirs of defective and full-length HTLV-1c proviral genomes.** (A) Genome coverage of proviral genomes enriched from isolated CD4^+^ T-cell phenotypes (lung homing T_LH_, regulatory T_REG_, CCR4− control T_CCR4−_), stratified and coloured by donor and HAPD status. Average coverage shown as a percentage. Coverage of genome is represented by opacity. (B) log_10_ copies of proviral genes *gag*, *env*, *hbz* and *tax*, per 10^6^ cells in sputum, detected by ddPCR in 4 HTLV-1c+ participants from Mparntwe. Each colour represents different donor.

The enrichment of HTLV-1c provirus in the activated and expanded phenotypic T_LH_ cells and the association with HAPD prompted investigation of the proviral landscape at high resolution in this critical reservoir. We mapped the provirus sequences assembled from this phenotype of two participants (P085 HAPD-, P136 HAPD+) to the HTLV-1c consensus genome, and detected a defective provirus landscape. Many provirus clones of the T_LH_ proxy reservoir in both donors retained some, or all, of *gag* and *pX* regions, however P136 also maintained some coverage in *pol* and *env* coding regions. Overall, P136 displayed greater genomic coverage (35.56%) than P085 (27.06%) in these cells (Fig. 4A). To confirm that T_LH_ cells with defective provirus migrate to the lungs, we extracted gDNA from sputum of HTLV-1c+ participants (n = 4). Importantly, we found that *hbz and tax* were detected at higher levels than *env* in sputum (Fig. 4B), confirming that defective provirus in HTLV-1c+ cells traffic to and infiltrate the lungs and likely contribute to pulmonary disease.

In the T_REG_ proxy reservoir, we also detected highly defective provirus. P136 (HAPD+) showed greatest retention of the *pX* region in this reservoir, and greater overall genomic coverage in these cells (mean = 28.38%) when compared to P085 (HAPD-) (20.78%). However, the genomic retention in T_REG_ remained lower than the matched T_LH_ proxy reservoir (Fig. 4A).

Remarkably, in the T_CCR4−_ reservoir of P136 (HAPD+), we detected 7/7 full-length provirus (Fig. 4A). Unfortunately, we were unable to amplify provirus from T_CCR4−_ cells of HAPD− donor P085 due to a low number of infected cells isolated in this reservoir. However, in P136 these proviral sequences displayed over 99.3% homology with the Central Australian HTLV-1c consensus sequence, indicating genetic stability in this reservoir.^21^ Analysis of the open reading frames (ORFs) and spliced products determined these full-length proviruses could produce intact HTLV-1 protein products, with the exception of Pol, which contained the same cytosine to thymine mutation (Q28X) in 7/7 proviruses, which is predicted to truncate Pol upstream of the reverse transcription domain. Examination of this region in other proviruses from our dataset determined the Q28X Pol mutation was also present for P136 in T_LH_ and PBMC sequences, and in 4 proviruses from P009 (HAPD+), P085 (HAPD+) and P095 (HAPD-). Analysis of all publicly available HTLV-1c sequences saw this variant present in sequences from the Solomon Islands^12^ and Central Australia,^11^^,^^21^ and in an HTLV-1a assembly from Central China.^51^ Recurrent detection of this variant in multiple datasets raises the possibility that stop codon suppression may be utilised at this site, enabling translational readthrough to produce the full Pol protein, as has been observed with Murine leukaemia virus.^52^ Notwithstanding, these data raise the possibility that CCR4^−^CD45RA^−^CD4^+^ T-cells may represent a cellular reservoir of HTLV-1c that maintains structural integrity of the proviral landscape and infectivity.

### Defective proviruses maintain HBZ open reading frames and encode junction-spanning and fusion products

**Fig. 5:**
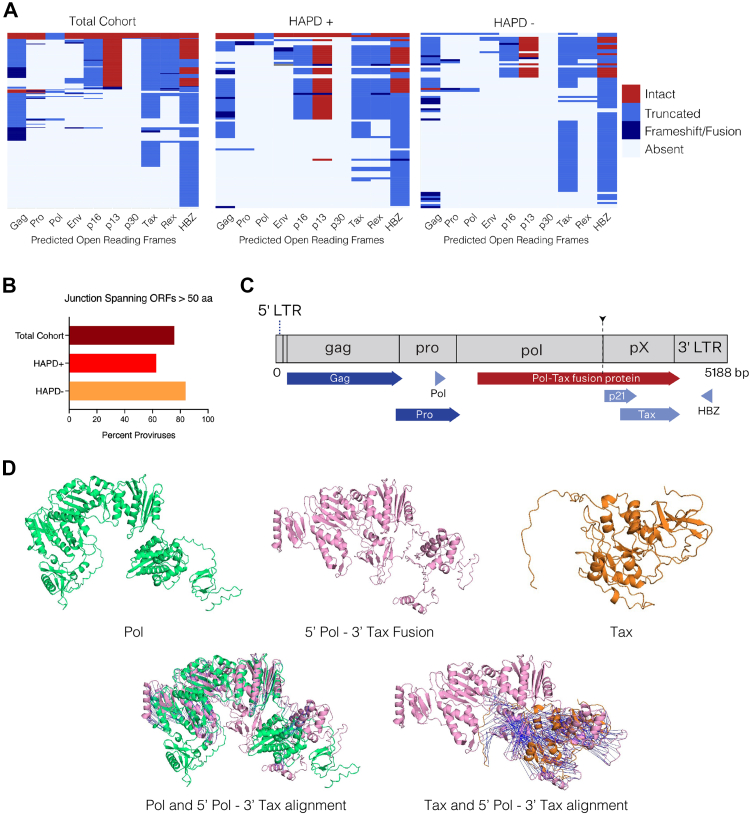
**Coding potential of defective HTLV-1c proviruses.** (A) Heatmap depicting coding potential of 260 proviral genomes from 6 HTLV-1c+ participants, scored as intact, truncated (including N and C terminal truncations), frameshift or fusion proteins, or absent in the population (left) (n = 6) and stratified by HAPD status (right). Each row represents an individual proviral genome, and each column represents an open reading frame. (B) Quantification of proviral genomes that contain one or more novel predicted open reading frames encoded by sequences that span the breakpoint junctions of defective genomes, as a proportion of the total number of genomes (260) and stratified by HAPD status. (C) A schematic representation of an open reading frame that spans a breakpoint junction in a defective HTLV-1c genome. Proviral sequences are depicted in grey, canonical open reading frames in blue, truncated open reading frame predictions in light blue, and putative fusion open reading frames in red. (D) 3D modelling of HTLV-1c Pol (green), Tax (orange), and putative 5′Pol-3′Tax fusion protein (pink), as a product of a novel ORF spanning the deletion junction of a defective provirus. Blue lines indicate regions of structural alignment on overlays. Predicted structures were generated in AlphaFold v2.

The strong association between HTLV-1c infection and pulmonary disease,^5^^,^^15^^,^^16^ and between higher HTLV-1c PVL and more extensive lung injury or bronchiectasis-related deaths,^20^^,^^53^ suggests that defective proviruses contribute to immune dysfunction. To this end, we analysed the HTLV-1 ORFs of each individual clone from PBMCs, T_LH_, T_REG_ and T_CCR4−_, by aligning the provirus sequences to the Central Australian HTLV-1c consensus genome. p13 and spliced HBZ were the most frequently intact products (30.77%, 23.07%, respectively), while HBZ and Tax ORFs displayed the most potential truncated products (71.52%, 61.54%, respectively). Intact doubly spliced HTLV-1 mRNA products (*tax*, *rex*, *p30, p16*^23^) were largely missing in the defective provirus landscape. A small number of HTLV-1 products contained point mutation or frameshifts arising in indels (Fig. 5A). As the proviral landscape in the humanised mouse model harboured more extensive internal deletions, the HTLV-1 ORFs were largely absent. Truncated HBZ and Gag were the most common remaining products (59.72%, 45.83%, respectively) (Supplementary Fig. S12A).

Interestingly, most defective proviruses (human infection = 75.8%, hu-NSG mice = 37.6%) contained uncharacterised ORFs of ≥50 amino acids, that spanned the deletion, chimeric, or inversion provirus breakpoint junctions (Fig. 5B and C, Supplementary Fig. S12B). The predicted products of these unique junction-spanning ORFs vary; some encode HTLV-1 viral fusion (Fig. 5C) or viral:cellular fusion proteins, others HTLV-1 proteins with an extended open reading frame, or completely frameshifted products. We used AlphaFoldv2^54^ to model the structures of these putative viral fusion proteins and demonstrated potential structural divergence from individual HTLV-1 proteins (Fig. 5D). This could affect the proteins, RNAs and pathways with which these proteins interact, and in turn transmission and pathogenesis.

### Chimeric provirus genomes represent a means for HTLV-1c to influence host gene expression

**Fig. 6:**
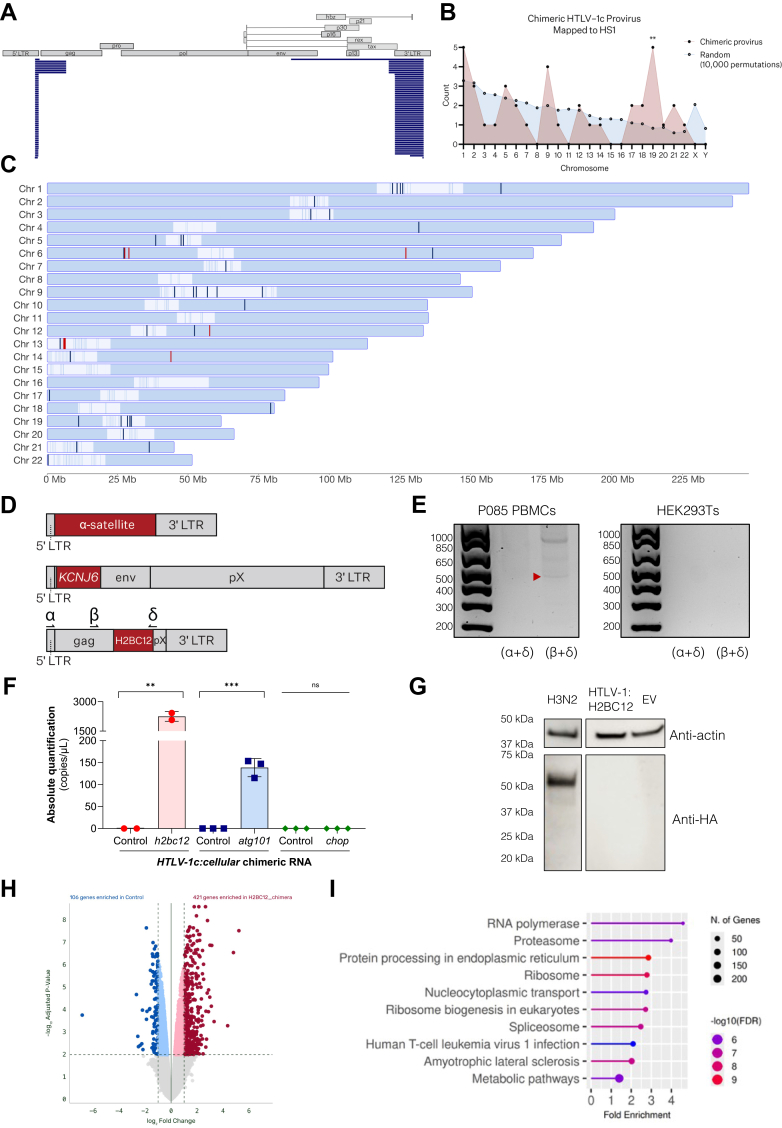
**Chimeric HTLV-1c:cellular genomes place host coding and non-coding sequences between HTLV-1c LTR promoters.** (A) Multiple sequence alignment of the proviral segments of the HTLV-1c:cellular chimeric proviruses, to the Central Australian HTLV-1c consensus genome sequence. 51 chimeric genomes were amplified from five people living with HTLV-1c. (B) Quantification of chromosomal distribution of alignments of the cellular segments of chimeric genomes to the HS1 human genome assembly, compared to a random distribution generated by 10,000 permutations (p = 0.002, permutation test). (C) Chromosomal distribution of the cellular segments of the chimeric genomes when aligned to the HS1 human genome assembly. Each chromosome is represented as a blue box, centromeric repeats are represented in white, and coordinates of the alignment are represented as navy blue lines for chimeras originating from clinical samples, and red originating from humanised mice. (D) Schematic representations of HTLV-1c:cellular chimeric proviral genomes incorporating centromeric (top), intronic (middle), or exonic (bottom) host segments. Symbols (α, β and δ) represent the approximate position of primers used to PCR validate the HTLV-1c:H2BC12 provirus. Proviral sequences are represented in grey, and host sequences in red. (E) PCR validation of HTLV-1c:H2BC12. The gel image on the left shows the junction spanning PCR results in DNA isolated from PBMCs of P085, and the gel image on the right shows the junction spanning PCR results in DNA isolated from HEK293Ts as a negative control. Ladder band sizes are as indicated. The red-filled triangle indicates an on-target product amplified with the β + δ primer pair, confirmed by ONT long-read sequencing. Attempts to PCR amplify the HTLV-1c:H2BC12 provirus with the α + δ primer pair did not return a product. (F) Expression of *HTLV-1c:H2BC12* (red), *HTLV-1c:ATG101* (blue) and *HTLV-1c:CHOP* (green) chimeric proviruses by RT-qPCR in HEK293T cells transduced with respective chimeric proviral constructs, relative to empty vector controls. Bar graphs depict mean ± SEM. (G) Western blot for HA-tag in H3N2 influenza viral lysate as an antibody control (left), whole cell lysates from HEK293T cells transduced with HTLV-1c:H2BC12-HA construct (middle, expected size 22 kDa) or empty vector control (right), with actin as a loading control. (H) Volcano plot showing differential expression between Jurkat cells transduced with chimeric *HTLV-1c:H2BC12* proviral genome compared with an empty vector control. Each dot represents a gene, those in red represent genes that are upregulated (FDR < 0.01) and those in blue, downregulated in *HTLV-1c:H2BC12* cells compared with controls. (I) Top 10 KEGG pathway enrichment of genes differentially expressed (edgeR, FDR < 0.01) in Jurkat cells transduced with chimeric *HTLV-1c:H2BC12* genomes compared with empty vector controls, ordered by fold enrichment. Statistical significance in (F) was assessed with unpaired T-tests, non-significant (ns) p > 0.05, ∗p < 0.05, ∗∗p < 0.01, ∗∗∗p < 0.001.

A striking feature of the HTLV-1c proviral landscape was the presence of chimeric genomes containing host-derived sequences internalised between HTLV-1c LTRs, representing 20.3% of the sequenced proviral landscape. Coordinates of the host-derived sequences relative to the T2T-CHM13 genome assembly are listed in Supplementary Table S5, while actual host sequences themselves are not disclosed, in accordance with HREC-17-2930. The chimeras were predominantly comprised of cellular sequences flanked by the HTLV-1c LTRs. However, 8/53 chimeric proviruses included some HTLV-1c coding sequence. Notably, one contained the entire *pX* region (Fig. 6A). The size of the internal cellular segments ranged from 107 to 3262 bp and aligned to positions broadly distributed throughout the human genome proportional to chromosome size. Cellular segments that mapped to gene-rich chromosome 19 were detected with increased frequency (p = 0.002) (Fig. 6B and C). Cellular segments of the chimeras were markedly enriched for centromeric repeats, accounting for 58.7% of the chimeric proviruses detected. Of the remaining chimeric proviruses, 6.8% contained intergenic cellular sequences, 9.1% intronic, and 22.7% coding sequence. Of note, seven chimeric proviruses amplified from a single participant contained the coding region of the histone H2BC12 gene (Fig. 6D). The chimeric *HTLV-1c:H2BC12* proviral sequence was independently validated by junction PCR and ONT long-read sequencing from bulk PBMC gDNA (Fig. 6E).

The identification of chimeric HTLV-1c:cellular proviruses was recapitulated in hu-NSG mice splenocytes (Supplementary Table S5). The HTLV-1c chimeric provirus breakpoints in hu-NSG mice varied; some occurred at LTR junctions, while others occurred in the *gag* and *pX* regions (Supplementary Fig. S9C). The hu-NSG mice chimeric proviruses contained cellular segments ranging in size from 61 to 371 bp, mapping to predominantly non-coding RNAs in the human genome (Fig. 6C). Notably, five chimeras contained the coding region of C/EBP homologous protein (CHOP), three chimeras contained sequences of cytosolic glutamic acid-CTC tRNA (tRNA-Glu anticodon CTC), and another three tRNA-Asp anticodon GTC (Supplementary Fig. S9D). The chimeric provirus-CHOP and provirus-tRNA-Glu sequences were independently validated by junction PCR and sequencing from bulk splenocyte gDNA (Supplementary Fig. S9E).

To assess whether chimeric proviruses could confer functional effects on infected cells, we introduced selected chimeric proviral genomes into HEK293T cells through lentiviral integration. We focused on *HTLV-1c:H2BC12*, detected seven times in donor P085, as it is predicted to encode a mutant HIST1H2BK protein; a C-terminal HA tag was added to enable detection of the potential fusion protein. We also introduced *HTLV-1c:ATG101*, identified in donor P015, and *HTLV-1c:CHOP*, detected five times in hu-NSG mice, as both proviruses incorporated 5′UTR sequences suggesting possible non-coding regulatory activity.^55^ Expression of predicted *HTLV-1c:H2BC12* and *HTLV-1c:ATG101*, but not *HTLV-1c:CHOP* transcripts were confirmed by RT-qPCR (Fig. 6F). The predicted HTLV-1c:HIST1H2BK-HA fusion protein was not detected by Western blot, suggesting it is either not translated or unstable and potentially degraded as defective ribosomal products (DRiPs) and served as peptide for antigen presentation by MHC class I^56^ (Fig. 6G). RNA-seq analysis in Jurkat cells harbouring the chimeric *HTLV-1:H2BC12* genome compared with an empty vector control, shows that the top dysregulated pathways are involved in transcription, protein processing and metabolism, consistent with a hypothesis of defective protein production (Fig. 6H and I, Supplementary Table S6). Taken together, these findings show that chimeric HTLV-1c:cellular proviruses represent a previously unrecognised form of viral genome rearrangement and provide an alternate mechanism by which HTLV-1 can modulate host gene expression.

## Discussion

HAPD was first reported to follow HTLV-1a infection where lymphocyte infiltration of lung tissue^57^^,^^58^ and HTLV-1a mRNA expression in BALF,^59^^,^^60^ accompany an inflammatory cytokine milieu.^19^ While less frequently reported in HTLV-1a infection, HAPD is commonly associated with HTLV-1c infection in First Nations adults in Central Australia.^5^^,^^18^^,^^20^ As in individuals infected with subtype-A infection, HTLV-1c provirus has been detected in sputum samples from First Nations people in Central Australia,^24^ indicating infiltration of infected cells into lung tissue. Animal model studies, including macaque^23^ and humanised mice,^22^ further demonstrate a propensity of subtype-C infection to drive lung disease. The mechanisms by which HTLV-1-infected cells, in particular subtype-C, drive lung pathogenesis, and what factors increase individual risk of HAPD, have remained unclear. We characterised a chronically activated CD4^+^ T-cell phenotype which migrates to the lungs, that harbours defective provirus with retained *hbz* expression potential. We observed upregulation of CD49d on CD4^+^ T-cells in HAPD+ participants, which could facilitate transmigration of these HTLV-1c-infected cells into pulmonary tissue through interaction with VCAM-1 on endothelial cells. Chronic inflammation at the tissue barrier triggers the shedding of VCAM-1 from endothelial cells,^41^ and we observed increased plasma sVCAM-1 in HTLV-1c+ samples, consistent with reports in HTLV-1a-associated disease.^61^ Together, these findings suggest a model in which chronically activated, *hbz*-expressing CD4^+^ T-cells may traffic to the lungs via CD49d:VCAM-1 interactions, contributing to chronic inflammation, tissue damage, and progression to bronchiectasis.^19^^,^^62^ We propose sVCAM-1 could be utilised as a biomarker for disease progression and therapeutic evaluation. Similarly, targeting CD49d as a therapeutic approach may mitigate the infiltration of these activated and pathogenic cells into the lungs, and similarly in subtype-A infection into the central nervous system (CNS),^63^ to avoid disease progression to HAPD and HAM. Evaluation of anti-CD49d antibodies in humanised mouse models of HTLV-1 could provide critical preclinical data towards translation.

We have developed SPA-ONT-seq which enables sequencing of individual, contiguous, haplotype-resolved HTLV-1 genomes. This approach can be applied to other HTLV-1 subtypes, extending insights from bulk short-read, Cas9-based targeted long-read protocols, and primer walking assemblies by enabling single-genome analysis and improved detection of structural variation.^21^^,^^28^^,^^47^, ^48^, ^49^, ^50^^,^^64^ Our characterisation of the HTLV-1c proviral landscape in six participants from Central Australia identified genetic aberrations associated with HAPD. Across all CD4^+^ T-cell reservoirs examined, we observed greater retention of the 3′LTR and *pX* genome regions of the HTLV-1c provirus in HAPD+ compared to HAPD− donors. Notably, the expression potential of *hbz* was more frequently preserved in the defective genomes of HAPD+ participants. Preferential retention of 3′ proviral regions including *hbz* has been previously documented in HTLV-1a infection, particularly in the context of ATL.^64^ Our findings extend these observations to HTLV-1c and demonstrate their relevance to HAPD pathogenesis. Given that *hbz* RNA and protein products act on viral and cellular pathways to promote viral persistence, T-cell proliferation, migration to sites of inflammation, and host gene expression dysregulation,^65^ our findings suggest a possible role for antisense *hbz* expression in HAPD pathogenesis. In HTLV-1a infection, *hbz* RNA transcript levels correlate with HAM disease severity,^66^ suggesting that *hbz* products may contribute to HTLV-1–associated inflammatory diseases more broadly. As large internal deletions ablate regulatory enhancers and silencers,^67^^,^^68^ it will be important to map the transcriptional landscape of defective HTLV-1c proviruses. Nevertheless, in subtype-A infection, defective proviruses with large indels have been shown to produce mRNA transcripts encoding truncated proteins in patients with ATL.^49^^,^^69^ This raises the possibility that both full-length and defective genomes contribute functional *hbz* transcripts and proteins relevant to HAPD. Taken together, the frequent retention of the *hbz* gene and its 3′LTR promoter in defective proviruses positions *hbz* gene products as promising and conserved targets for the development of HTLV-1 therapeutics, including new RNA-based approaches.^70^

Therapeutic strategies to target HTLV-1 infection must consider replication-competent reservoirs of infection. We found HTLV-1c provirus in a high proportion of chronically activated T_CCR4−_ cells. In one donor where sufficient CCR4^−^ cells were available for analysis, we detected only full-length provirus in this reservoir by SPA-ONT-seq. This preliminary observation requires validation in larger cohorts, to investigate the possibility that CCR4^−^CD4^+^ T-cells contain replication-competent provirus contributing to the persistence of HTLV-1c infection. CCR4^+^ expression is commonly associated with cell trafficking to sites of inflammation,^71^ so CCR4^−^ cells infected with intact provirus might remain in circulation where they can facilitate cell-to-cell transmission. This will be addressed in future cohort studies with high-quality primary samples, across multiple viral subtypes.

Outside of the T_CCR4−_ reservoir, we detected extensive genetic damage to the provirus. That this occurred in humanised mouse infection lacking any adaptive immune responses likely excludes immune selection of deleted variants. Instead, HTLV-1 infection promotes genomic instability through direct interactions with the host genome, which can in turn lead to alterations in the proviral genome itself.^72^ The relative paucity of single nucleotide polymorphisms (SNPs) alongside extensive structural variation is consistent with defects in DNA repair or replication stress. Reflecting this process, breakpoints displayed regions of 4–10 bp microhomology in 31% proviruses, a signature that may arise through multiple mechanisms. Microhomology at deletion junctions can indicate either template switching events during reverse transcription,^73^^,^^74^ or microhomology-mediated end joining (MMEJ), an error-prone DNA double-strand break repair pathway.^75^ Evidence from HTLV-1a infection indicates that somatic mutations accumulating during clonal proliferation, rather than integration-related mutations, account for most defective proviruses,^72^ supporting a predominantly post-integration model. Consistent with this interpretation, we observed high levels of defective provirus in chronically activated T-cells, which significantly expand in HTLV-1c infection proportional to PVL and are thus more likely to acquire replication errors through successive cell divisions. We hypothesise that viral-driven genomic instability is exacerbated by an accumulation of replicative mutations due to increased proliferation upon infection. Conversely, the CCR4^−^ phenotype does not undergo comparable expansion, potentially preserving full-length provirus in this reservoir through reduced exposure to replication-associated mutagenesis. However, the contribution of reverse transcription errors, integration-associated DNA repair, and post-integration proliferative stress to the proviral deletion landscape remains to be fully elucidated and may vary across cellular contexts and disease states.

The presence of HTLV-1c:cellular chimeric proviruses further reflects the genomic instability inherent to HTLV-1 infection. We propose that most recombination events generating chimeric genomes are deleterious, leading to loss of proviral function, and are neutral with respect to host fitness. In some cases, however, the capture of host sequences may enable replication-defective proviruses to acquire additional regulatory functions. We provided evidence that cellular sequences placed under the control of viral regulatory elements can drive the ectopic expression of cellular sequences to modulate endogenous host gene expression. Moreover, the presence of chimeric RNAs and proteins or defective ribosomal products (DRiPs) may trigger an inflammatory response or even autoimmunity. The mechanisms by which chimeric proviral genomes contribute to pathogenesis remains unknown; however, their frequency suggests this warrants further investigation.

This study has several limitations. The cross-sectional and hospital-based cohort will require replication in larger community-based studies to validate these important findings. While we demonstrated a shared lung-homing phenotype in paired blood and bronchoalveolar samples from HTLV-1c+ humanised mice, analyses in matched clinical specimens from humans will be necessary to confirm tissue localisation and functional relevance. Future studies should consider tracking continuous clinical measurements used to grade pulmonary disease. As only subtype-C is endemic in Central Australia, we could not assess whether similar tropism or risk associations occur with subtype-A. Logistical constraints limited the viability of some clinical samples, precluding transcriptomic analyses. Finally, while the SPA assay greatly improved resolution of the HTLV-1 proviral landscape, it selectively amplifies genomes retaining both LTRs, and enriches for lower molecular weight genomes, thereby underrepresenting truncated and full-length proviruses. Without integration-site resolution, we cannot definitively establish clonal relationships between proviruses sharing identical structures, validate the genomic context of all chimeric events, or fully elucidate the mechanisms generating structural variants. Despite these constraints, this study provides a high-resolution, haplotype resolved view of the HTLV-1c reservoir, establishing a framework for future population-based and longitudinal studies of viral persistence and disease.

In summary, we have shown that chronically activated HTLV-1c-infected CD4^+^ T-cells that migrate to the lungs induce pathogenesis and the development of HAPD. We identified CD49d expression on these T-cells as a potential therapeutic target to block the migration of HTLV-1-infected cells from circulation into tissues, such as the lungs and CNS. Furthermore, the extensive chronic activation of all CD4^+^ phenotypes in HTLV-1c+ participants likely results in overall T-cell dysfunction and diminishes an effective host immune response, contributing to poorer health outcomes and lower survival rate of HTLV-1c-infected individuals. We developed SPA-ONT-seq to capture individual, haplotype-resolved HTLV-1 proviral genomes, and detected extensive internal proviral deletions in HTLV-1c+ participants from Central Australia and a humanised mouse model of infection, which displayed hallmarks of microhomology mediated-end joining. We found that the *hbz* gene is preferentially retained in HAPD, suggesting that *hbz* expression is implicated in the development of pulmonary disease. And finally, we have described HTLV-1c:cellular chimeric proviruses, which represent a form of viral genome rearrangement and provide an alternate mechanism by which HTLV-1 can modulate host gene expression or prompt immunopathogenesis. This study provides direction for future investigations into potential therapeutic targets, including *hbz,* and efforts to ultimately eliminate the reservoir of integrated provirus.

## Contributors

AH, NJ, GK and DP designed the research project. AH and NJ wrote the manuscript. GK, PE, JC, JZ, KNN, AY, NH, LW, LM, MD, LW, DS, SC, MRT, AHV, AC, MD, MP, GJF, LE, and DP were involved in review and editing. AH, NJ, PE, JC, KNN, AY, NH, LW, LM, MD, LW, DS, SC, MRT, and AHV conducted sample collection, preparation and quality control. DP, GK, LE, JZ, AC, GJF, MD and MP provided resources and guidance. AH and NJ analysed the data. AH, NJ, GFJ, LE and DP have access to and verified all underlying data. AH, NJ, PE, MP, LE, GJF and DP were involved in funding acquisition. All authors read and approved the final manuscript.

## Data sharing statement

Oxford Nanopore Technologies sequencing data and RNA sequencing data generated by this study were deposited in the Sequence Read Archive (SRA), under BioProject SRA: PRJNA1210085. Sequences which relate to genomic information of First Nation’s peoples are not available in accordance with HREC-17-2930. Please contact NTHREC (NTHREC@menzies.edu.au) for further information. Summary statistics and information describing these data, are published in this manuscript.

Any other data requests and queries should be directed to Professor Damian Purcell (dfjp@unimelb.edu.au).

## Declaration of interests

The authors declare no competing interests.

## Appendix A Supplementary data

Supplemental Western blots
