# Supplementary figures and images for: Immunophenotype and proviral landscape of HTLV-1c infection and pulmonary disease
Source: eBioMedicine. 2026 Jul 23;130:106403. doi: 10.1016/j.ebiom.2026.106403 (PMC13427564; doi:10.1016/j.ebiom.2026.106403)

Anti-HA

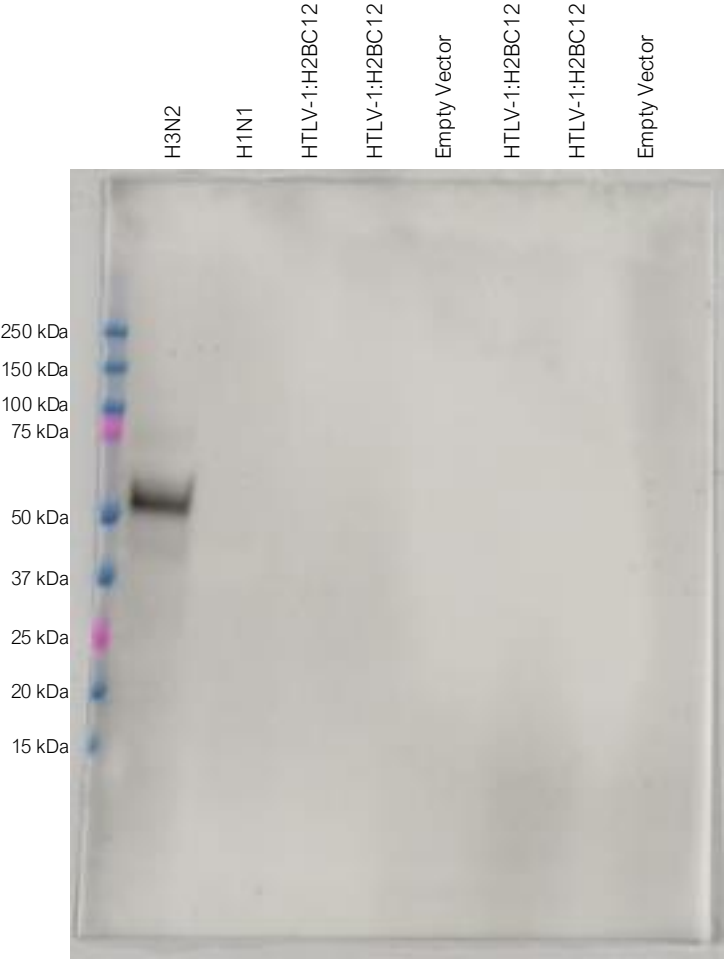

Anti-β-actin

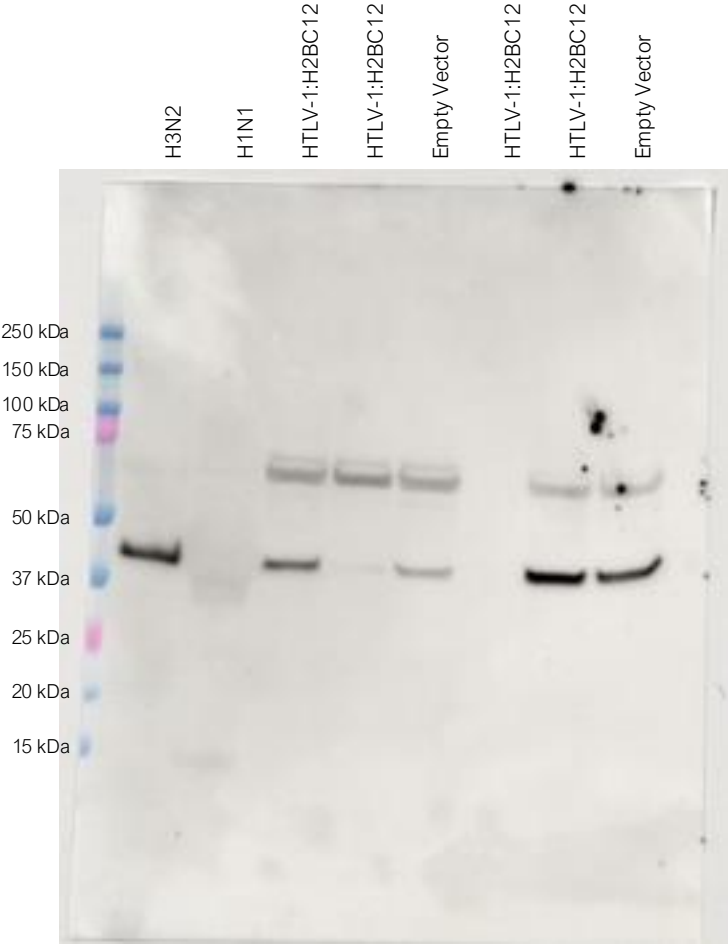

Supplement: Supplemental Western blots [file mmc1.pdf]
