# Supplementary material for: Immunophenotype and proviral landscape of HTLV-1c infection and pulmonary disease
Source: eBioMedicine. 2026 Jul 23;130:106403. doi: 10.1016/j.ebiom.2026.106403 (PMC13427564; doi:10.1016/j.ebiom.2026.106403)
Supplement: Supplementary Tables S1–S6 [file mmc4.pdf]

Supplementary Table 1 – Oligonucleotide primers and probes used in this study.

Name	Target	Strand	Sequence (5’-3’)
Droplet digital PCR assay			
ODP 3083	HTLV-1c *gag* region	Forward primer	CAAATGAAGGACCTACAGGC
ODP 3084	HTLV-1c *gag* region	Reverse primer	TATCTAGCTGCTGGTGATGG
ODP 3321	HTLV-1c gag region	Probe	6FAM-ACCATCCGGCTTGCAGT-MGBNFQ
ODP 4033	HTLV-1c *env* region	Forward primer	TACCATGCCACCTATTCCCT
ODP 4034	HTLV-1c *env* region	Reverse primer	TTCAGGCGTGAGACTTCTTG
ODP 4035	HTLV-1c *env* region	Probe	6FAM-ACAGGGGCCGTCTCCAGCCCC-MGBNFQ
ODP 3986	HTLV-1c *hbz* region	Forward primer	CCTCTTTTTCCCGCTCTCTT
ODP 3987	HTLV-1c *hbz* region	Reverse primer	GAGGGAGGAGGAGGAATCTG
ODP 3988	HTLV-1c *hbz* region	Probe	6FAM-TTCGCTTTCTCTTCTCCTCG-MGBNFQ
ODP 3085	HTLV-1c *tax* region	Forward primer	TCCAGGCCTTATTTGGACAT
ODP 3086	HTLV-1c *tax* region	Reverse primer	CGTGTGAGAGTAGGACTGAG
ODP 3318	HTLV-1c *tax* region	Probe	6FAM-CATGATTTCCGGGCCTTGC-MGBNFQ
Single provirus amplification (SPA) assay			
ODP 3847	HTLV-1c 5’LTR	Forward primer	CACGTCTGACTGCCGGCTTG
ODP 3848	HTLV-1c 3’LTR	Reverse primer	CGGAACTTTCGATCGGTAGC
ODP 3849	HTLV-1c 5’LTR	Forward nested primer	AAGCACCGGCACCCTTACT
ODP 3850	HTLV-1c 3’LTR	Reverse nested primer	CGCAGAACAGAAAACGAAA
Chimeric provirus validation PCR			
HTLV1c_nested_FWD	HTLV-1c 5’LTR	Forward primer	AAGCACCGGCACCCTTACT
HTLV1c_gag_fwd	HTLV-1c gag region	Forward primer	ATGGGCCAAATCTTCCCTCGTA
DDIT3_2_R	DDIT3	Reverse primer	TCATGTTAAGATGAGCGGGTG
DDIT3_3’_junction_R	DDIT3-HTLV-1c 3’ LTR chimeric junction	Reverse primer	GGGAGCGCCGG CGAGAGA
tRNA_hbz_LTR_R	tRNA-Glu- HTLV-1c *hbz* chimeric junction	Reverse primer	CCATGGTCAGTATCATCTGGTTCCCTGAC
Quantitative PCR			

ODP 4426	HTLV-1c *gag* qPCR	Forward primer	CCCAAATCAGCCGTGCT
ODP 4427	HTLV-1c *gag* qPCR	Reverse primer	GAGGTCTAGTAGGAGGGCATC
ODP 4429	HTLV-1c *env* qPCR	Forward primer	CCGTCTAGCCCTATGGACA
ODP 4430	HTLV-1c *env* qPCR	Reverse primer	CCACTGGCGGCTATTAAGAC
ODP 4431	HTLV-1c *tax/rex* qPCR	Forward primer	CAACACCATGGCCCACT
ODP 4432	HTLV-1c *tax/rex* qPCR	Reverse primer	GGGGTCCCAGGTGATCT
ODP 4433	HTLV-1c *hbz* qPCR	Forward primer	CCTCAGGGCCATTTCGG
ODP 4434	HTLV-1c *hbz* qPCR	Reverse primer	CTAGAGATAGCACACCGTCAAG
ODP 2004	Human *HPRT*	Forward primer	GACCAGTCAACAGGGGACAT
ODP 2005	Human *HPRT*	Reverse primer	CCTGACCAAGGAAAGCAAAG
qpcr_Ddit3_F	DDIT3	Forward primer	CTTTGGCTCTGTCGCTGCCA
qpcr_Hatag_R	HA-Tag	Reverse primer	AGCGTAATCTGGAACATCGTATGG
qpcr_h2bc12_F	*H2BC12*	Forward primer	GCCTGGCGCATTACAACAAG
qpcr_h2bc12_junction_R	*H2BC12*:HTLV-1c breakpoint junction	Reverse primer	ATAGGCCTTGGTGCCCTCG
qpcr_Atg_F	*ATG101*	Forward primer	TTACAAGGGCCTGGCATGTG
qpcr_Atg_Junction_R	*ATG101*:HTLV-1c breakpoint junction	Reverse primer	TGGGGCCTATGGTCAGTATCAAATTAT

Note: 6FAM – 5’ fluorescent dye; MGBNFQ – 3’ minor groove binding non-fluorescent quencher

Supplementary Table 1: Oligonucleotide primers and probes used in this study. Droplet digital (dd)PCR assay includes primers and probes for HTLV-1c *gag*, *env*, *hbz* and *tax*. Single provirus amplification (SPA) assay includes nested primers for near full-length genome amplification. Chimeric provirus validation PCR includes primers spanning HTLV-1c:cellular junctions. Quantitative (q) PCR primers targeting the specific mRNA transcripts of *gag* and spliced *hbz*, *tax* and *env*, and housekeeping HRPT gene.

Supplementary Table 2 - HTLV-1c provirus annotations and breakpoints assembled from human donors

Provirus	Genome structure	Length (bp)	Identity to consensus (%)	1st proviral segment			2nd proviral segment			3rd proviral segment			4th proviral segment		
				Start	*End*	*Strand*	*Start*	*End*	*Strand*	*Start*	*End*	*Strand*	*Start*	*End*	*Strand*
P136_Treg_provirus13	Indel	257	100	0	45	+	8006	8219	+						
P085_Lung_provirus15	Indel	313	99.57	0	81	+	7987	8219	+						
P085_Lung_provirus17	Indel	313	99.57	0	81	+	7987	8219	+						
P085_Lung_provirus12	Indel	313	99.57	0	81	+	7987	8219	+						
P085_Treg_provirus09	Indel	313	99.57	0	81	+	7987	8219	+						
P085_Lung_provirus38	Indel	313	99.57	0	81	+	7987	8219	+						
P085_Treg_provirus12	Indel	380	98.34	0	362	+	8201	8219	+						
P085_PBMC_provirus40	Indel	381	98.62	0	362	+	8201	8219	+						
P085_Treg_provirus02	Indel	381	98.62	0	362	+	8201	8219	+						
P015_PBMC_provirus03	Indel	381	98.62	0	362	+	8201	8219	+						
P085_PBMC_provirus32	Indel	380	98.34	0	362	+	8201	8219	+						
P085_Lung_provirus11	Indel	313	99.57	0	81	+	7987	8219	+						
P085_PBMC_provirus08	Chimeric	481	99.32	0	80	+	7923	8219	+						
P085_PBMC_provirus28	Indel	612	98.97	0	218	+	1740	2032	+	8115	8219	+			
P015_PBMC_provirus09	Indel	654	99.29	0	90	+	7653	8219	+						
P095_PBMC_provirus18	Indel	654	98.61	0	431	+	7983	8219	+						
P136_Lung_provirus13	Indel	653	98.84	0	431	+	7983	8219	+						
P015_PBMC_provirus17	Indel	654	99.29	0	90	+	7653	8219	+						
P136_Lung_provirus11	Indel	652	98.84	0	431	+	7983	8219	+						
P015_PBMC_provirus10	Indel	654	99.29	0	90	+	7653	8219	+						
P095_PBMC_provirus17	Indel	677	94.36	0	431	+	7983	8219	+						
P015_PBMC_provirus27	Indel	654	99.29	0	90	+	7653	8219	+						
P085_PBMC_provirus48	Indel	654	98.84	0	431	+	7983	8219	+						
P085_PBMC_provirus35	Indel	381	98.62	0	362	+	8201	8219	+						
P015_PBMC_provirus28	Indel	875	98.55	0	52	+	7395	8219	+						
P015_PBMC_provirus12	Indel	875	98.67	0	52	+	7395	8219	+						
P015_PBMC_provirus29	Indel	874	98.42	0	52	+	7395	8219	+						
P015_PBMC_provirus39	Indel	875	98.55	0	52	+	7395	8219	+						
P015_PBMC_provirus41	Indel	874	98.42	0	52	+	7395	8219	+						

P085_Lung_provirus37	Indel	951	98.57	0	699	+	7964	8219	+						
P070_PBMC_provirus22	Indel	975	98.56	0	79	+	7319	8219	+						
P070_PBMC_provirus23	Indel	975	98.67	0	79	+	7319	8219	+						
P070_PBMC_provirus32	Indel	974	98.67	0	79	+	7319	8219	+						
P070_PBMC_provirus34	Indel	974	98.56	0	79	+	7319	8219	+						
P070_PBMC_provirus03	Indel	975	98.78	0	79	+	7319	8219	+						
P070_PBMC_provirus05	Indel	975	98.78	0	79	+	7319	8219	+						
P070_PBMC_provirus25	Indel	975	98.78	0	79	+	7319	8219	+						
P070_PBMC_provirus26	Indel	975	98.56	0	79	+	7319	8219	+						
P070_PBMC_provirus17	Indel	976	98.67	0	79	+	7319	8219	+						
P070_PBMC_provirus29	Indel	976	98.89	0	79	+	7319	8219	+						
P070_PBMC_provirus14	Indel	976	98.78	0	79	+	7319	8219	+						
P070_PBMC_provirus01	Indel	976	98.89	0	79	+	7319	8219	+						
P070_PBMC_provirus10	Indel	976	98.89	0	79	+	7319	8219	+						
P070_PBMC_provirus11	Indel	975	98.67	0	79	+	7319	8219	+						
P070_PBMC_provirus27	Indel	976	98.89	0	79	+	7319	8219	+						
P070_PBMC_provirus12	Indel	976	98.67	0	79	+	7319	8219	+						
P070_PBMC_provirus13	Indel	975	98.56	0	79	+	7319	8219	+						
P070_PBMC_provirus24	Indel	976	98.89	0	79	+	7319	8219	+						
P070_PBMC_provirus06	Indel	976	98.78	0	79	+	7319	8219	+						
P070_PBMC_provirus30	Indel	975	98.67	0	79	+	7319	8219	+						
P070_PBMC_provirus33	Indel	974	98.56	0	79	+	7319	8219	+						
P070_PBMC_provirus36	Indel	975	98.67	0	79	+	7319	8219	+						
P070_PBMC_provirus35	Indel	976	98.78	0	79	+	7319	8219	+						
P070_PBMC_provirus28	Indel	975	98.67	0	79	+	7319	8219	+						
P070_PBMC_provirus09	Indel	975	98.67	0	79	+	7319	8219	+						
P070_PBMC_provirus02	Indel	975	98.67	0	79	+	7319	8219	+						
P070_PBMC_provirus16	Indel	975	98.67	0	79	+	7319	8219	+						
P070_PBMC_provirus08	Indel	975	98.56	0	79	+	7319	8219	+						
P070_PBMC_provirus07	Indel	976	98.78	0	79	+	7319	8219	+						
P070_PBMC_provirus21	Indel	976	98.78	0	79	+	7319	8219	+						
P070_PBMC_provirus15	Indel	976	98.67	0	79	+	7319	8219	+						
P070_PBMC_provirus20	Indel	975	98.67	0	79	+	7319	8219	+						
P070_PBMC_provirus31	Indel	976	98.78	0	79	+	7319	8219	+						

P070_PBMC_provirus18	Indel	976	98.67	0	79	+	7319	8219	+						
P070_PBMC_provirus19	Indel	976	98.78	0	79	+	7319	8219	+						
P085_Lung_provirus36	Chimeric	961	98.52	0	77	+	7609	8219	+						
P085_PBMC_provirus11	Indel	951	98.57	0	699	+	7964	8219	+						
P085_Treg_provirus10	Chimeric	1030	98.85	0	79	+	7609	8219	+						
P095_PBMC_provirus19	Chimeric	1027	99.35	0	66	+	7608	8219	+						
P015_PBMC_provirus24	Chimeric	1014	99.02	0	79	+	7609	8219	+						
P015_PBMC_provirus23	Chimeric	1014	99.02	0	79	+	7609	8219	+						
P085_PBMC_provirus26	Chimeric	1028	98.85	0	80	+	7609	8219	+						
P085_PBMC_provirus05	Chimeric	1042	98.69	0	79	+	7609	8219	+						
P015_PBMC_provirus35	Chimeric	1155	99.02	0	79	+	7609	8219	+						
P070_PBMC_provirus04	Indel	1149	98.4	0	1063	+	8129	8219	+						
P085_PBMC_provirus15	Indel	889	98.02	0	282	+	1838	2139	+	3549	3797	+	8153	8219	+
P085_Lung_provirus03	Chimeric	1274	99.02	0	80	+	7608	8219	+						
P009_PBMC_provirus16	Indel	1303	98.69	0	89	+	6998	8219	+						
P085_Lung_provirus31	Indel	1350	99.03	0	925	+	7790	8219	+						
P015_PBMC_provirus08	Indel	1346	98.73	0	93	+	6962	8219	+						
P085_Lung_provirus28	Chimeric	1365	98.52	0	80	+	7609	8219	+						
P136_Treg_provirus04	Chimeric	1373	98.53	0	79	+	7609	8219	+						
P015_PBMC_provirus37	Chimeric	1363	98.85	0	79	+	7609	8219	+						
P136_PBMC_provirus12	Chimeric	1373	98.53	0	79	+	7609	8219	+						
P136_PBMC_provirus25	Chimeric	1373	99.35	0	80	+	7608	8219	+						
P085_PBMC_provirus09	Chimeric	1369	98.52	0	80	+	7609	8219	+						
P085_Lung_provirus34	Chimeric	1368	98.52	0	77	+	7609	8219	+						
P085_Lung_provirus06	Chimeric	1369	97.87	0	80	+	7609	8219	+						
P136_Treg_provirus11	Chimeric	1374	98.85	0	79	+	7609	8219	+						
P085_Lung_provirus27	Chimeric	1366	98.69	0	75	+	7609	8219	+						
P015_PBMC_provirus34	Indel	1345	98.65	0	93	+	6962	8219	+						
P085_Lung_provirus32	Chimeric	1390	99.02	0	80	+	7608	8219	+						
P085_PBMC_provirus36	Chimeric	1353	98.85	0	80	+	7608	8219	+						
P015_PBMC_provirus06	Indel	1345	98.65	0	93	+	6962	8219	+						
P015_PBMC_provirus11	Chimeric	1606	99.02	0	80	+	7609	8219	+						
P136_PBMC_provirus33	Chimeric	1639	99.18	0	79	+	7609	8219	+						
P136_PBMC_provirus17	Chimeric	1712	98.69	0	79	+	7609	8219	+						

P136_PBMC_provirus34	Indel	1719	99.33	0	80	+	6575	8219	+						
P015_PBMC_provirus15	Chimeric	1759	98.36	0	79	+	7609	8219	+						
P136_PBMC_provirus26	Chimeric	1713	99.18	0	80	+	7609	8219	+						
P015_PBMC_provirus36	Chimeric	1696	98.36	0	79	+	7609	8219	+						
P136_Lung_provirus03	Chimeric	1781	99.02	0	79	+	7609	8219	+						
P085_Treg_provirus08	Chimeric	1794	97.19	0	663	+	7475	8219	+						
P085_Treg_provirus11	Chimeric	1790	98.66	0	663	+	7475	8219	+						
P085_PBMC_provirus20	Chimeric	1794	98.66	0	663	+	7475	8219	+						
P015_PBMC_provirus38	Chimeric	1710	98.69	0	79	-	7608	8219	-						
P085_PBMC_provirus31	Indel	1794	98.77	0	507	+	6916	8219	+						
P085_Treg_provirus04	Chimeric	1797	98.79	0	663	+	7475	8219	+						
P085_PBMC_provirus24	Chimeric	1793	98.66	0	663	+	7475	8219	+						
P009_PBMC_provirus17	Indel	1756	98.3	0	1708	+	8161	8192	+						
P136_PBMC_provirus10	Chimeric	1827	99.35	0	80	+	7609	8219	+						
P085_Treg_provirus06	Chimeric	1797	98.79	0	663	+	7475	8219	+						
P015_PBMC_provirus32	Chimeric	1840	98.39	0	79	+	7597	8219	+						
P085_Treg_provirus07	Chimeric	1797	98.79	0	663	+	7475	8219	+						
P085_PBMC_provirus47	Indel	1793	98.77	0	507	+	6916	8219	+						
P085_Lung_provirus08	Indel	1831	98.73	0	516	+	6878	8219	+						
P085_Lung_provirus26	Indel	1900	98.96	0	292	+	6591	8219	+						
P136_PBMC_provirus09	Chimera	1827	99.35	0	80	+	7609	8219	+						
P015_PBMC_provirus31	Chimeric	2050	98.36	0	80	+	7609	8219	+						
P136_PBMC_provirus30	Chimeric	2052	98.69	0	79	+	7609	8219	+						
P136_Treg_provirus02	Indel	1980	99.5	0	603	+	6833	8219	+						
P085_PBMC_provirus38	Chimeric	2080	98.52	0	78	+	7609	8219	+						
P136_Lung_provirus16	Chimeric	2053	99.18	0	80	+	7609	8219	+						
P085_PBMC_provirus25	Chimeric	2044	98.69	0	80	+	7609	8219	+						
P085_PBMC_provirus44	Indel	2102	97.24	0	2032	+	8115	8219	+						
P136_Treg_provirus09	Chimeric	2053	98.85	0	79	+	7609	8219	+						
P085_PBMC_provirus27	Indel	2125	98.47	0	2032	+	8115	8219	+						
P136_Lung_provirus04	Indel	2232	95.15	0	1387	+	7344	8219	+						
P085_PBMC_provirus03	Indel	2232	98.77	0	1138	+	7117	8219	+						
P085_Treg_provirus05	Indel	2232	98.68	0	1138	+	7117	8219	+						
P009_PBMC_provirus14	Indel	2151	98.91	0	689	+	6747	8219	+						

P085_PBMC_provirus29	Indel	2235	98.86	0	1138	+	7117	8219	+						
P085_Lung_provirus23	Indel	2237	99.03	0	1138	+	7117	8219	+						
P136_PBMC_provirus06	Indel	2245	98.99	0	1387	+	7345	8219	+						
P136_PBMC_provirus31	Indel	2244	98.92	0	1387	+	7345	8219	+						
P136_PBMC_provirus05	Indel	2245	98.92	0	1387	+	7345	8219	+						
P085_Lung_provirus09	Indel	2237	99.03	0	1138	+	7117	8219	+						
P085_Lung_provirus25	Indel	2237	99.03	0	1138	+	7117	8219	+						
P085_Lung_provirus20	Indel	2237	99.03	0	1138	+	7117	8219	+						
P136_Lung_provirus02	Indel	2243	98.85	0	1387	+	7345	8219	+						
P085_Lung_provirus29	Indel	2237	99.03	0	1138	+	7117	8219	+						
P085_PBMC_provirus45	Indel	2237	99.03	0	1138	+	7117	8219	+						
P136_Lung_provirus08	Indel	2245	98.92	0	1387	+	7345	8219	+						
P136_PBMC_provirus07	Indel	2245	98.99	0	1387	+	7345	8219	+						
P136_Lung_provirus07	Indel	2244	98.92	0	1387	+	7345	8219	+						
P136_Lung_provirus21	Indel	2244	98.99	0	1387	+	7345	8219	+						
P085_Lung_provirus16	Indel	2237	99.03	0	1138	+	7117	8219	+						
P085_PBMC_provirus30	Indel	2235	98.86	0	1138	+	7117	8219	+						
P136_Lung_provirus05	Indel	2245	98.99	0	1387	+	7345	8219	+						
P136_PBMC_provirus15	Indel	2245	98.99	0	1387	+	7345	8219	+						
P136_PBMC_provirus24	Indel	2243	98.99	0	1387	+	7345	8219	+						
P085_Lung_provirus10	Indel	2238	99.03	0	1138	+	7117	8219	+						
P085_Lung_provirus41	Indel	2237	99.03	0	1138	+	7117	8219	+						
P085_PBMC_provirus43	Indel	2235	98.86	0	1138	+	7117	8219	+						
P136_PBMC_provirus18	Indel	2244	98.92	0	1387	+	7345	8219	+						
P085_Lung_provirus14	Indel	2238	99.03	0	1138	+	7117	8219	+						
P136_Lung_provirus20	Indel	2243	98.92	0	1387	+	7345	8219	+						
P136_Lung_provirus22	Indel	2246	99.06	0	1387	+	7345	8219	+						
P136_PBMC_provirus27	Chimeric	2401	99.02	0	80	+	7609	8219	+						
P015_PBMC_provirus01	Indel	2488	98.77	0	396	+	6113	8219	+						
P015_PBMC_provirus26	Indel	2492	98.81	0	396	+	6113	8219	+						
P095_PBMC_provirus10	Indel	2499	99.59	0	53	+	5767	8219	+						
P095_PBMC_provirus02	Indel	2500	99.59	0	53	+	5767	8219	+						
P015_PBMC_provirus13	Indel	2550	98.94	0	671	+	6335	8219	+						
P015_PBMC_provirus18	Indel	2548	98.89	0	671	+	6335	8219	+						

P015_PBMC_provirus05	Indel	2550	98.94	0	671	+	6335	8219	+						
P015_PBMC_provirus19	Indel	2492	98.86	0	396	+	6113	8219	+						
P095_PBMC_provirus11	Indel	2499	99.59	0	53	+	5767	8219	+						
P015_PBMC_provirus40	Indel	2546	98.99	0	671	+	6335	8219	+						
P015_PBMC_provirus02	Indel	2549	98.94	0	671	+	6335	8219	+						
P015_PBMC_provirus21	Indel	2551	99.1	0	671	+	6335	8219	+						
P015_PBMC_provirus33	Indel	2518	98.78	0	671	+	6337	8219	+						
P095_PBMC_provirus15	Indel	2617	99.39	0	330	+	5939	8219	+						
P015_PBMC_provirus07	Indel	2549	98.94	0	671	+	6335	8219	+						
P015_PBMC_provirus25	Indel	2549	98.99	0	671	+	6335	8219	+						
P095_PBMC_provirus16	Indel	2617	99.48	0	330	+	5939	8219	+						
P085_Treg_provirus03	Indel	2751	98.71	0	48	+	5512	8219	+						
P085_Lung_provirus21	Indel	2751	98.71	0	48	+	5512	8219	+						
P095_PBMC_provirus13	Chimeric	2742	99.35	0	80	+	7609	8219	+						
P015_PBMC_provirus30	Indel	2769	98.47	0	425	+	5867	8219	+						
P095_PBMC_provirus01	Indel	2723	99.56	0	253	+	5742	8219	+						
P136_PBMC_provirus11	Indel	2759	99.39	0	482	+	5926	8219	+						
P085_PBMC_provirus39	Indel	2716	94.23	0	1567	+	6984	8219	+						
P136_PBMC_provirus21	Indel	2759	99.39	0	482	+	5926	8219	+						
P136_Treg_provirus03	Indel	2759	99.43	0	482	+	5926	8219	+						
P136_Lung_provirus09	Indel	2758	99.39	0	482	+	5926	8219	+						
P136_PBMC_provirus14	Indel	2759	99.39	0	482	+	5926	8219	+						
P136_Treg_provirus15	Indel	2758	99.39	0	482	+	5926	8219	+						
P136_Treg_provirus10	Indel	2758	99.35	0	482	+	5926	8219	+						
P136_PBMC_provirus16	Indel	2758	99.35	0	482	+	5926	8219	+						
P136_PBMC_provirus28	Indel	2759	99.35	0	482	+	5926	8219	+						
P136_PBMC_provirus20	Indel	2758	99.35	0	482	+	5926	8219	+						
P136_Treg_provirus01	Indel	2760	99.43	0	482	+	5926	8219	+						
P136_Treg_provirus05	Indel	2759	99.39	0	482	+	5926	8219	+						
P136_PBMC_provirus32	Indel	2759	99.39	0	482	+	5926	8219	+						
P085_Lung_provirus19	Indel	2750	98.67	0	48	+	5512	8219	+						
P085_PBMC_provirus01	Indel	2782	98.35	0	1567	+	6984	8219	+						
P136_Treg_provirus07	Indel	2880	96.75	0	1677	+	7020	8219	+						
P136_PBMC_provirus02	Chimeric	2954	98.85	0	79	+	7609	8219	+						

P085_Lung_provirus02	Indel	2857	98.4	0	1612	+	6962	8219	+						
P136_PBMC_provirus23	Chimeric	2903	99.18	0	80	+	7609	8219	+						
P085_PBMC_provirus07	Indel	3023	97.65	0	671	+	5836	8219	+						
P085_Treg_provirus01	Indel	3050	98.28	0	671	+	5836	8219	+						
P085_Lung_provirus01	Indel	3051	98.62	0	671	+	5836	8219	+						
P085_PBMC_provirus46	Indel	3053	98.62	0	671	+	5836	8219	+						
P085_Lung_provirus07	Indel	3055	98.7	0	671	+	5836	8219	+						
P085_Lung_provirus13	Indel	3056	98.7	0	671	+	5836	8219	+						
P085_Lung_provirus22	Indel	3056	98.7	0	671	+	5836	8219	+						
P085_PBMC_provirus41	Indel	3052	98.66	0	671	+	5836	8219	+						
P085_Lung_provirus35	Indel	3056	98.74	0	671	+	5836	8219	+						
P085_PBMC_provirus34	Indel	3119	97.98	0	1382	+	4404	5027	+	7134	8219	+			
P085_Lung_provirus30	Indel	3057	98.74	0	671	+	5836	8219	+						
P085_PBMC_provirus33	Indel	3054	98.7	0	671	+	5836	8219	+						
P085_PBMC_provirus37	Indel	3054	98.74	0	671	+	5836	8219	+						
P085_PBMC_provirus42	Indel	3054	98.66	0	671	+	5836	8219	+						
P085_PBMC_provirus21	Indel	3048	98.53	0	671	+	5836	8219	+						
P085_Lung_provirus24	Indel	3056	98.7	0	671	+	5836	8219	+						
P015_PBMC_provirus16	Indel	3159	98.76	0	663	+	5715	8219	+						
P085_Lung_provirus04	Indel	3056	98.74	0	671	+	5836	8219	+						
P015_PBMC_provirus20	Indel	3164	98.92	0	663	+	5715	8219	+						
P085_Lung_provirus18	Indel	3056	98.7	0	671	+	5836	8219	+						
P085_Lung_provirus33	Indel	3129	98.84	0	1382	+	4404	5027	+	7134	8219	+			
P085_Lung_provirus40	Indel	3127	98.77	0	1382	+	4404	5027	+	7134	8219	+			
P009_PBMC_provirus15	Indel	3159	98.86	0	718	+	5773	8219	+						
P085_Lung_provirus05	Indel	3129	98.84	0	1382	+	4404	5027	+	7134	8219	+			
P085_Lung_provirus44	Indel	3133	98.56	0	2499	+	7566	8219	+						
P009_PBMC_provirus04	Chimeric	2132	93.98	0	81	-	8192	8219	-						
P009_PBMC_provirus06	Inversion	3251	97.85	0	99	+	1973	2116	-	2554	2777	+	5400	8219	+
P009_PBMC_provirus13	Indel	3271	98.89	0	329	+	5259	8219	+						
P136_PBMC_provirus04	Inversion	3180	99.42	0	99	+	100	623	-	5646	8219	+			
P009_PBMC_provirus10	Inversion	3250	98.86	0	99	+	1973	2116	-	2554	2777	+	5400	8219	+
P009_PBMC_provirus09	Inversion	3250	98.86	0	99	+	1973	2116	-	2554	2777	+	5400	8219	+
P136_PBMC_provirus35	Indel	3519	99.4	0	350	+	5065	8219	+						

P136_PBMC_provirus01	Inversion	3477	99.61	0	86	+	1413	2020	-	5425	8219	+			
P136_Lung_provirus06	Indel	3489	99.54	0	908	+	5632	8219	+						
P136_PBMC_provirus19	Indel	3537	99.49	0	1010	+	5687	8219	+						
P136_PBMC_provirus13	Indel	3489	99.5	0	908	+	5632	8219	+						
P009_PBMC_provirus12	Indel	3733	98.7	0	1282	+	5755	8219	+						
P136_PBMC_provirus08	Indel	3699	98.74	0	1984	+	2024	2093	+	7387	8219	+			
P136_PBMC_provirus03	Indel	3700	98.74	0	1984	+	2024	2093	+	7387	8219	+			
P136_PBMC_provirus22	Indel	3729	99.51	0	98	+	4485	8219	+						
P095_PBMC_provirus06	Chimeric	3766	99.51	0	99	+	5410	8219	+						
P095_PBMC_provirus09	Indel	3913	99.63	0	435	+	4740	8219	+						
P095_PBMC_provirus12	Indel	3909	99.51	0	435	+	4740	8219	+						
P136_Lung_provirus15	Indel	3911	99.48	0	1625	+	5921	8219	+						
P136_Lung_provirus01	Indel	4153	99.57	0	662	+	4276	8219	+						
P095_PBMC_provirus04	Chimeric	3955	99.35	0	80	+	7609	8219	+						
P136_PBMC_provirus37	Indel	5174	99.1	0	3759	+	6791	8219	+						
P136_Lung_provirus12	Indel	5600	99.04	0	3008	+	5164	8219	+						
P136_Lung_provirus18	Indel	6268	99.02	0	6130	+	8047	8219	+						
P095_PBMC_provirus03	Indel	6377	98.78	0	5312	+	7144	8219	+						
P009_PBMC_provirus01	Full-length	8212	99.48	0	8219	+									
P009_PBMC_provirus03	Full-length	8212	99.44	0	8219	+									
P136_CCR4_provirus01	Full-length	8211	99.45	0	8219	+									
P136_CCR4_provirus02	Full-length	8209	99.43	0	8219	+									
P136_CCR4_provirus03	Full-length	8213	99.48	0	8219	+									
P136_CCR4_provirus04	Full-length	8208	99.42	0	8219	+									
P136_CCR4_provirus05	Full-length	8211	99.45	0	8219	+									
P136_CCR4_provirus06	Full-length	8210	99.44	0	8219	+									

P136_CCR4_provirus07	Full-length	8213	99.48	0	8219	+												

Indel=insertion/deletion

Supplementary Table 2: HTLV-1c provirus annotations and breakpoints assembled from human donors. HTLV-1c consensus genome sequences assembled from each donor were aligned to the amplified region within the HTLV-1c consensus sequence obtained from Hirons et al.[21]. Each provirus is listed with its genome structure (full-length, indel, inversion or chimeric), assembled amplicon length, and percent identity with the published consensus sequence. Coordinates and strand of each segment within each provirus is listed, relative to the primer binding sites on the consensus sequence.

Supplementary Table 3 – Microhomology of HTLV-1c defective provirus breakpoints

Provirus	MH size (bp)	MH sequence
Human donors		
P085_Treg_provirus03	6	CCCCCA
P085_Lung_provirus26	10	AAATGGCCCT
P009_PBMC_provirus13	8	CTACTCCC
P095_PBMC_provirus18	8	CCGCCGCC
P136_Lung_provirus13	8	CCGCCGCC
P136_Lung_provirus11	8	CCGCCGCC
P095_PBMC_provirus17	8	CCGCCGCC
P085_PBMC_provirus48	8	CCGCCGCC
P136_PBMC_provirus11	5	CAGAT
P136_PBMC_provirus21	5	CAGAT
P136_Treg_provirus03	5	CAGAT
P136_Lung_provirus09	5	CAGAT
P136_PBMC_provirus14	5	CAGAT
P136_Treg_provirus15	5	CAGAT
P136_Treg_provirus10	5	CAGAT
P136_PBMC_provirus16	5	CAGAT
P136_PBMC_provirus28	5	CAGAT
P136_PBMC_provirus20	5	CAGAT
P136_Treg_provirus01	5	CAGAT
P136_Treg_provirus05	5	CAGAT
P136_PBMC_provirus32	5	CAGAT
P085_PBMC_provirus31	4	TACA
P085_PBMC_provirus47	4	TACA
P085_PBMC_provirus03	6	CCCCCA
P085_PBMC_provirus29	6	CCCCCA
P085_Lung_provirus23	6	CCCCCA
P085_Lung_provirus09	6	CCCCCA
P085_Lung_provirus25	6	CCCCCA
P085_Lung_provirus20	6	CCCCCA
P085_Lung_provirus29	6	CAACCC
P085_PBMC_provirus45	6	CCCCCA
P085_Lung_provirus16	6	CCCCCA
P085_PBMC_provirus30	6	CCCCCA
P085_Treg_provirus05	6	CCCCCA
P085_Lung_provirus10	6	CCCCCA
P085_Lung_provirus41	6	CCCCCA
P085_PBMC_provirus43	6	CCCCCA
P085_Lung_provirus14	6	CCCCCA
P009_PBMC_provirus12	6	AAGACC
P085_PBMC_provirus34	9	GCCCCTCCT
P136_PBMC_provirus31	6	CAACCC
P136_PBMC_provirus05	6	CAACCC
P136_Lung_provirus02	6	CAACCC
P136_Lung_provirus08	6	CAACCC
P136_PBMC_provirus07	6	CAACCC

P136_Lung_provirus07	6	CAACCC
P136_Lung_provirus21	6	CAACCC
P136_Lung_provirus05	6	CAACCC
P136_PBMC_provirus15	6	CAACCC
P136_PBMC_provirus24	6	CAACCC
P136_PBMC_provirus18	6	CAACCC
P136_Lung_provirus20	6	CAACCC
P136_Lung_provirus22	6	CAACCC
P085_PBMC_provirus39	4	TACA
P136_Lung_provirus15	9	ACTCCCCTC
P085_Lung_provirus44	6	CCAACA
P136_PBMC_provirus37	10	AACATCAGAT

MH=microhomology

Supplementary Table 3: Microhomology of HTLV-1c defective provirus breakpoints. Sequences of ± 10bp at defective provirus breakpoint junctions were assessed for microhomology (MH). All defective proviruses containing microhomology breakpoints are listed from human donors, detailing size and sequence.

Supplementary Table 4 – HTLV-1c provirus annotations and breakpoints assembled from humanised mice samples

Provirus	Genome structure	Length	Identity to consensus (%)	1st proviral segment			2nd proviral segment		
				Start	*End*	*Strand*	*Start*	*End*	*Strand*
M86_Splenocytes_provirus25	Indel	171	100	0	79	+	8127	8219	+
M84_Splenocytes_provirus02	Indel	302	99.42	0	173	+	8089	8219	+
M84_Splenocytes_provirus26	Indel	323	99.57	0	234	+	8126	8219	+
M84_Splenocytes_provirus16	Indel	324	99.57	0	234	+	8126	8219	+
M86_Splenocytes_provirus08	Indel	336	100	0	91	+	7962	8219	+
M86_Splenocytes_provirus14	Indel	349	99.5	0	152	+	8022	8219	+
M84_Splenocytes_provirus33	Chimeric	417	99.26	0	84	+	7953	8219	+
M84_Splenocytes_provirus34	Chimeric	417	99.26	0	84	+	7953	8219	+
M84_Splenocytes_provirus13	Chimeric	418	99.63	0	84	+	7953	8219	+
M84_Splenocytes_provirus30	Indel	447	99.04	0	417	+	8200	8219	+
M84_Splenocytes_provirus09	Chimeric	459	99.67	0	84	+	7912	8219	+
M84_Splenocytes_provirus36	Chimeric	459	99.67	0	84	+	7912	8219	+
M86_Splenocytes_provirus28	Indel	471	98.98	0	392	+	8136	8219	+
M86_Splenocytes_provirus11	Indel	471	98.98	0	392	+	8136	8219	+
M86_Splenocytes_provirus01	Indel	471	98.72	0	392	+	8136	8219	+
M86_Splenocytes_provirus24	Indel	471	98.72	0	392	+	8136	8219	+
M86_Splenocytes_provirus30	Indel	471	98.98	0	392	+	8136	8219	+
M86_Splenocytes_provirus31	Indel	471	98.98	0	392	+	8136	8219	+
M86_Splenocytes_provirus36	Indel	471	98.72	0	392	+	8136	8219	+
M86_Splenocytes_provirus18	Indel	472	99.23	0	392	+	8136	8219	+
M84_Splenocytes_provirus10	Indel	489	99.03	0	412	+	8140	8219	+
M86_Splenocytes_provirus20	Indel	543	99.44	0	187	+	7859	8219	+
M86_Splenocytes_provirus35	Indel	543	99.44	0	187	+	7859	8219	+
M86_Splenocytes_provirus37	Indel	544	99.72	0	187	+	7859	8219	+
M86_Splenocytes_provirus06	Indel	563	84.65	0	95	+	6681	8219	+
M84_Splenocytes_provirus31	Indel	566	98.59	0	498	+	8146	8219	+
M84_Splenocytes_provirus20	Indel	569	99.2	0	498	+	8146	8219	+

M86_Splenocytes_provirus26	Indel	688	99.35	0	80	+	7605	8219	+
M84_Splenocytes_provirus04	Indel	689	99.02	0	80	+	7605	8219	+
M84_Splenocytes_provirus25	Indel	690	99.19	0	80	+	7605	8219	+
M84_Splenocytes_provirus05	Indel	691	99.51	0	81	+	7607	8219	+
M86_Splenocytes_provirus15	Indel	691	99.51	0	81	+	7607	8219	+
M84_Splenocytes_provirus07	Indel	691	99.18	0	81	+	7607	8219	+
M84_Splenocytes_provirus01	Indel	691	99.35	0	81	+	7607	8219	+
M84_Splenocytes_provirus14	Indel	692	99.67	0	81	+	7607	8219	+
M84_Splenocytes_provirus22	Indel	692	99.67	0	81	+	7607	8219	+
M86_Splenocytes_provirus12	Indel	692	99.51	0	81	+	7607	8219	+
M84_Splenocytes_provirus24	Indel	692	99.67	0	81	+	7607	8219	+
M86_Splenocytes_provirus07	Indel	692	99.67	0	81	+	7607	8219	+
M84_Splenocytes_provirus03	Indel	692	99.51	0	81	+	7607	8219	+
M86_Splenocytes_provirus29	Indel	698	94.44	0	80	+	7605	8219	+
M84_Splenocytes_provirus17	Indel	710	98.61	0	504	+	8006	8219	+
M86_Splenocytes_provirus03	Inversion	719	99.51	0	79	+	7611	8219	+
M84_Splenocytes_provirus35	Indel	737	98.87	0	707	+	8186	8219	+
M86_Splenocytes_provirus32	Indel	770	99.1	0	442	+	7888	8219	+
M86_Splenocytes_provirus17	Indel	776	99.01	0	708	+	8147	8219	+
M86_Splenocytes_provirus33	Indel	777	99.15	0	708	+	8147	8219	+
M84_Splenocytes_provirus11	Indel	881	99.56	0	191	+	7539	8218	+
M84_Splenocytes_provirus15	Indel	1026	99.47	0	278	+	7467	8219	+
M86_Splenocytes_provirus09	Chimeric	1030	98.97	0	870	+	8116	8219	+
M86_Splenocytes_provirus05	Chimeric	1030	99.08	0	870	+	8116	8219	+
M86_Splenocytes_provirus02	Chimeric	1031	99.08	0	870	+	8116	8219	+
M86_Splenocytes_provirus13	Chimeric	1031	99.08	0	870	+	8116	8219	+
M86_Splenocytes_provirus23	Chimeric	1032	99.2	0	870	+	8116	8219	+
M84_Splenocytes_provirus21	Chimeric	1034	99.67	0	361	+	7605	8219	+
M84_Splenocytes_provirus18	Indel	1056	99.61	0	36	+	7198	8219	+
M84_Splenocytes_provirus06	Indel	1057	99.61	0	36	+	7198	8219	+
M84_Splenocytes_provirus32	Indel	1057	99.71	0	36	+	7198	8219	+

M86_Splenocytes_provirus10	Chimeric	1059	99.35	0	79	+	7608	8219	+
M86_Splenocytes_provirus27	Indel	1062	99.63	0	262	+	7416	8219	+
M84_Splenocytes_provirus19	Indel	1067	99.03	0	925	+	8068	8219	+
M84_Splenocytes_provirus27	Indel	1170	98.93	0	840	+	7882	8219	+
M86_Splenocytes_provirus16	Indel	1172	99.67	0	285	+	7320	8219	+
M86_Splenocytes_provirus34	Indel	1264	97.57	0	654	+	7602	8219	+
M86_Splenocytes_provirus21	Indel	1298	99.53	0	245	+	7159	8219	+
M84_Splenocytes_provirus12	Indel	1414	99.6	0	667	+	7466	8219	+
M84_Splenocytes_provirus29	Chimeric	1494	99.55	0	91	+	7096	8219	+
M84_Splenocytes_provirus28	Indel	1540	99.44	0	124	+	6792	8219	+
M86_Splenocytes_provirus22	Indel	1581	99.27	0	80	+	6711	8219	+
M86_Splenocytes_provirus19	Indel	1603	98.92	0	1387	+	7991	8219	+
M86_Splenocytes_provirus04	Indel	1793	99.31	0	507	+	6914	8219	+
M84_Splenocytes_provirus23	Indel	1832	99.29	0	991	+	7376	8219	+

Supplementary Table 4: HTLV-1c provirus annotations and breakpoints assembled from humanised mice samples. HTLV-1c consensus genome sequences assembled from each mouse were aligned to the amplified region within the HTLV-1c consensus sequence obtained from Hirons et al.[21]. Each provirus is listed with its genome structure (full-length, indel, inversion or chimeric), assembled amplicon length, and percent identity with the published consensus sequence. Coordinates and strand of each segment within each provirus is listed, relative to the primer binding sites on the consensus sequence.

Supplementary Table 5: HTLV-1c:human chimeric provirus annotations. HTLV-1c consensus genome sequences assembled from each donor were aligned to the amplified region within the HTLV-1c consensus sequence obtained from Hirons et al.[21]. Regions that did not map to the HTLV-1c consensus were aligned to the human genome assembly HS1. All chimeric proviruses assembled from human donors and humanised mice in this study are listed. Annotations include cellular segment size, genomic coordinates of the cellular segment within HS1, and genomic features.

See excel file.

Supplementary Table 6: FDR, and LogFC of differentially expressed genes expressed in Jurkats that harbour an *HTLV-1:H2BC12* proviral genome relative to an empty vector control, related to Figure 6H and 6I.

See excel file.
