# Supplementary material for: Immunophenotype and proviral landscape of HTLV-1c infection and pulmonary disease
Source: eBioMedicine. 2026 Jul 23;130:106403. doi: 10.1016/j.ebiom.2026.106403 (PMC13427564; doi:10.1016/j.ebiom.2026.106403)
Supplement: Supplementary Figs. S1–S12 [file mmc5.pdf]

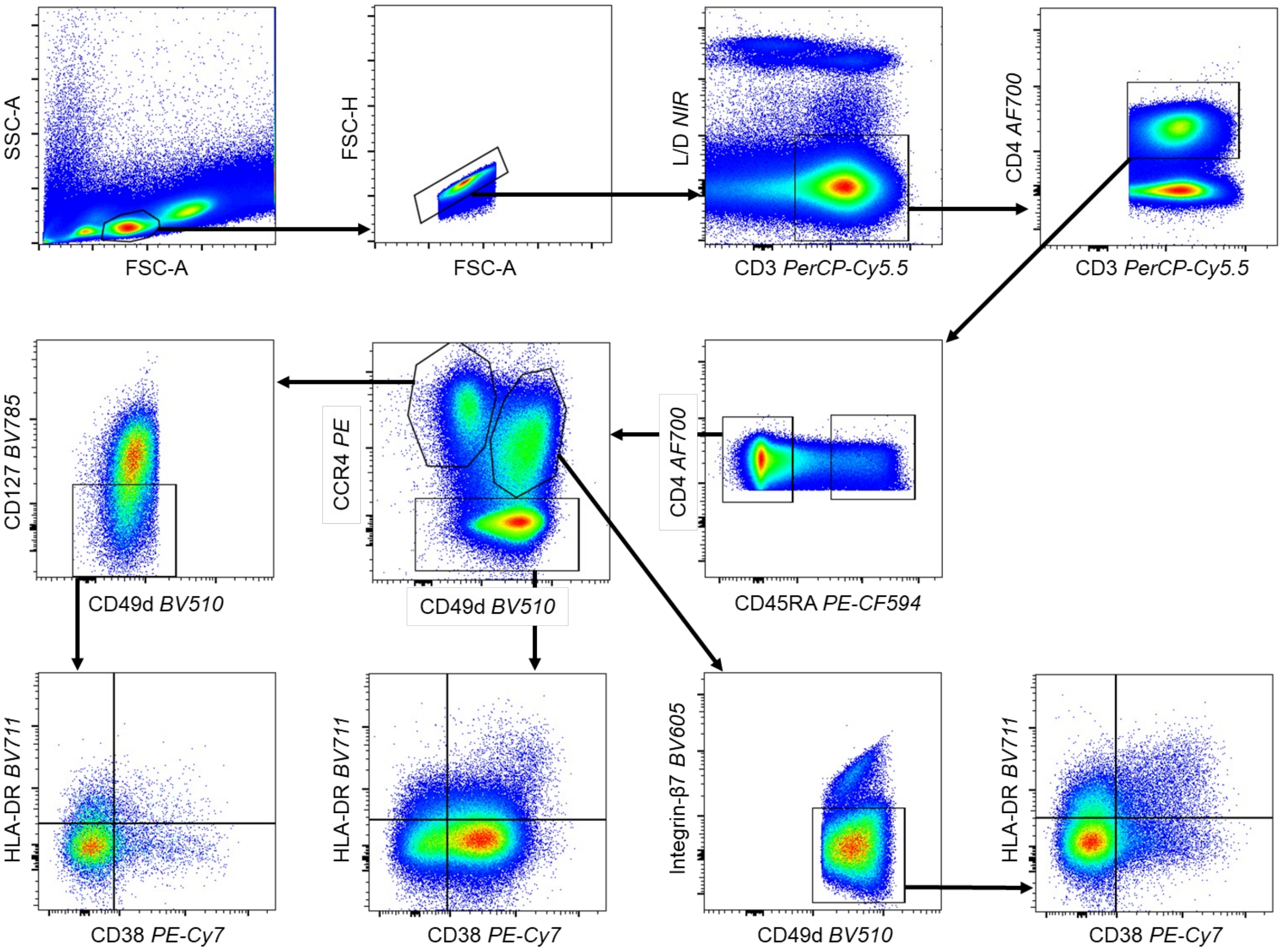

Supplementary Figure 1: Fluorescence activated cell sorting (FACS) gating strategy for HTLV-1c proviral reservoir phenotyping. Lymphocytes were identified by FSC-A vs SSC-A, and then doublet exclusion by FSC-A vs FSC-H, followed by selection of live CD3^{+} cells. CD4^{+}CD45RA^{-} cells were gated as the parent population for phenotyping analyses. The three phenotype populations sorted from CD4^{+}CC45RA^{-} cells were T_{LH} (lung homing proxy, CCR4^{+}CD49d^{+}Integrinβ7^{-}), T_{REG} (FOXP3^{+} proxy, CCR4^{+}CD49d^{-}CD127^{-}) and T_{CCR4-} (CCR4^{-}CD49d$^{+/-}$). These three populations were also gated on HLA-DR^{+}CD38^{+} for activation analyses.

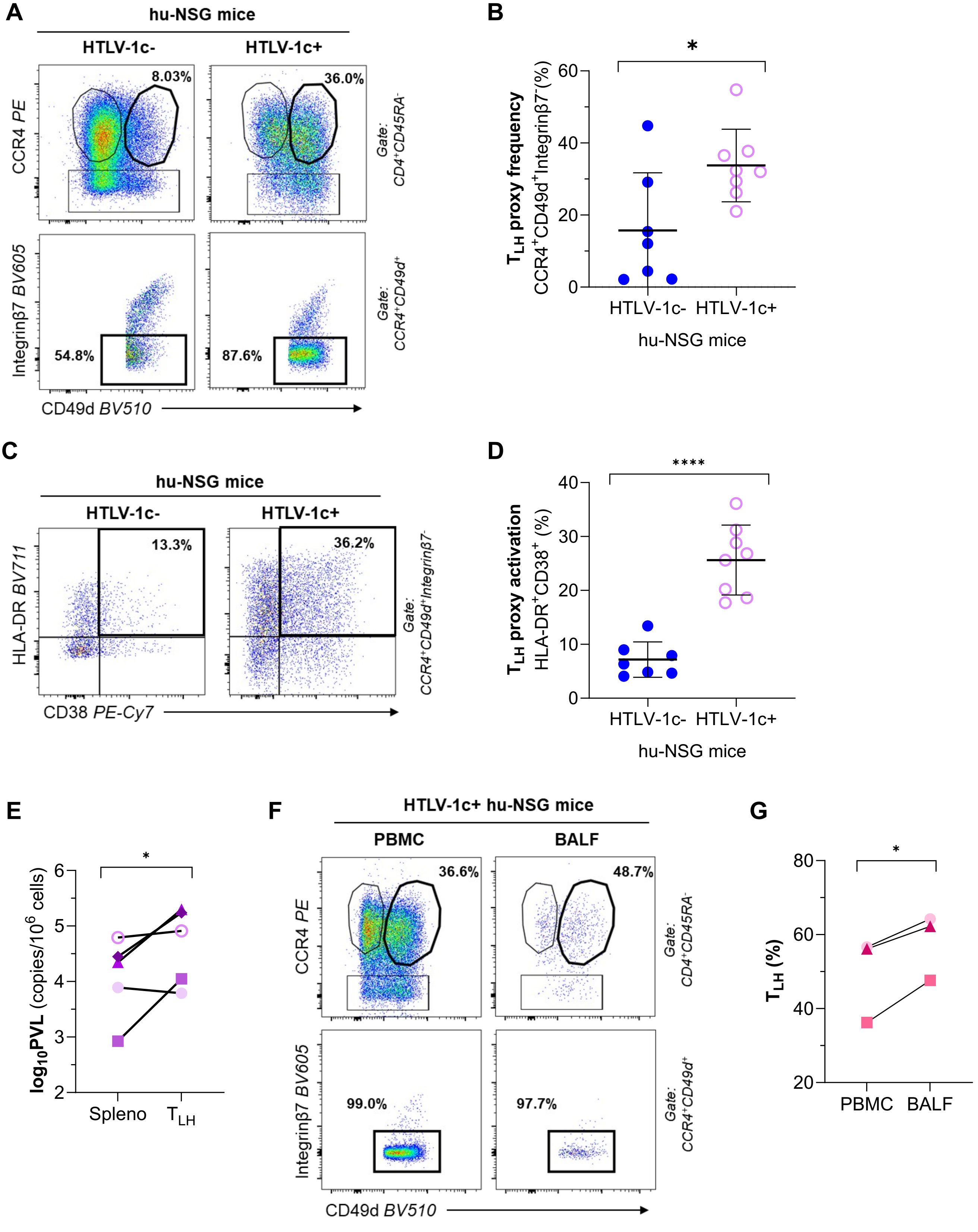
A
hu-NSG mice
HTLV-1c-
HTLV-1c+
8.03%
36.0%
CCR4 PE
Gate: CD4+CD45RA-
Integrinβ7 BV605
54.8%
87.6%
Gate: CCR4+CD49d+
CD49d BV510
B
*
TLH proxy frequency CCR4+CD49d+Integrinβ7-(%)
60
40
20
0
HTLV-1c-
HTLV-1c+
hu-NSG mice
C
hu-NSG mice
HTLV-1c-
HTLV-1c+
13.3%
36.2%
HLA-DR BV711
CD38 PE-Cy7
Gate: CCR4+CD49d+Integrinβ7-
D
****
TLH proxy activation HLA-DR+CD38+ (%)
40
30
20
10
0
HTLV-1c-
HTLV-1c+
hu-NSG mice
E
*
log10PVL (copies/10^6 cells)
6
5
4
3
2
Spleno
TLH
F
HTLV-1c+ hu-NSG mice
PBMC
BALF
36.6%
48.7%
CCR4 PE
Gate: CD4+CD45RA-
Integrinβ7 BV605
99.0%
97.7%
Gate: CCR4+CD49d+
CD49d BV510
G
*
TLH (%)
80
60
40
20
PBMC
BALF

Supplementary Figure 2: Validation of $CD4^+$ lung homing T-cell phenotype associated with disease in HTLV-1c+ humanised mice. (A) Flow cytometry plots of CCR4 (PE) and Integrin-β7 (BV605) with CD49d (BV510), showing expansion of $CCR4^+CD49d^+Integrin\beta7^-$ population for HTLV-1c+ humanised mice. (B) Frequency of lung homing proxy (T_{LH}) cells as a percentage of $CD4^+CD45RA^-$ population in splenocytes from an HTLV-1c humanised mouse model at 2 weeks post infection (wpi) (HTLV-1c- n=7, HTLV-1c+ n=8). Mean indicated by black line ± SD. (C) Flow cytometry plots of HLA-DR (BV711) and CD38 (PE-Cy7) in T_{LH} cells showing increased chronic activation in HTLV-1c+ mice. (D) Frequency of chronic activation marker expression ($HLA\text{-}DR^+CD38^+$) in T_{LH} reservoir of humanised mouse splenocytes. Mean indicated by black line ± SD. (E) log_{10}PVL (copies/10^6 cells) of matched T_{LH} and PBMC reservoirs in 5 HTLV-1c+ humanised mice, each colour represents different mouse. (F) Flow cytometry plots of CCR4 (PE) and Integrin-β7 (BV605) with CD49d (BV510) in an HTLV-1c+ hu-NSG mouse (6wpi), showing higher frequency of T_{LH} ($CCR4^+CD49d^+Integrin\beta7^-$) in BALF compared to PBMCs. (G) Frequency of T_{LH} proxy phenotype in matched BALF and PBMC samples from hu-NSG mice at 6wpi (n=3). Statistical significance in (B) and (D) was assessed with t-test with FDR adjustment, in (E) with RM one-way ANOVA with single pooled variance and FDR adjustment, and in (G) with paired t-test. *$p<0.05$, **$p<0.01$, **$p<0.001$.

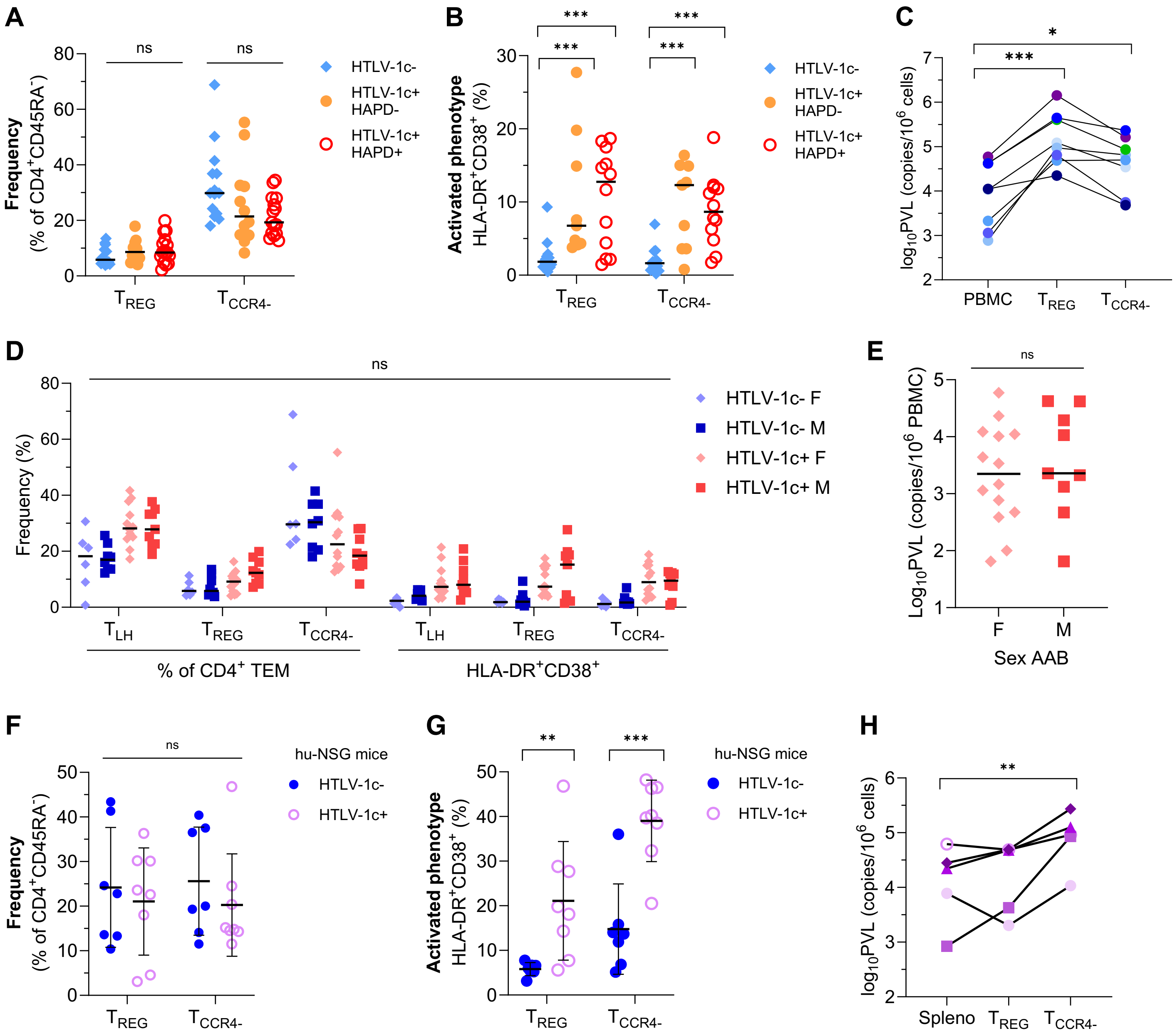
A
Frequency
(% of CD4+CD45RA-)
ns
ns
HTLV-1c-
HTLV-1c+ HAPD-
HTLV-1c+ HAPD+
TREG
TCCR4-
B
Activated phenotype
HLA-DR+CD38+ (%)
***
***
***
***
HTLV-1c-
HTLV-1c+ HAPD-
HTLV-1c+ HAPD+
TREG
TCCR4-
C
log10PVL (copies/10^6 cells)
*
***
PBMC
TREG
TCCR4-
D
Frequency (%)
ns
HTLV-1c- F
HTLV-1c- M
HTLV-1c+ F
HTLV-1c+ M
TLH
TREG
TCCR4-
% of CD4+ TEM
HLA-DR+CD38+
E
Log10PVL (copies/10^6 PBMC)
ns
F
M
Sex AAB
F
Frequency
(% of CD4+CD45RA-)
ns
hu-NSG mice
HTLV-1c-
HTLV-1c+
TREG
TCCR4-
G
Activated phenotype
HLA-DR+CD38+ (%)
**
***
hu-NSG mice
HTLV-1c-
HTLV-1c+
TREG
TCCR4-
H
log10PVL (copies/10^6 cells)
**
Spleno
TREG
TCCR4-

Supplementary Figure 3: Characteristics of T_{REG} and T_{CCR4-} CD4$^+$ T-cells in HTLV-1c infection. (A) Frequency of T_{REG} (FOXP3$^+$ proxy, CCR4$^+$CD49d$^-$ CD127$^-$) and T_{CCR4-} (CCR4-CD49d+/-) cells as a percentage of CD4$^+$CD45RA$^-$ population in 41 participants (HTLV-1c- n=14; HTLV-1c+ HAPD- n=12; HTLV-1c+ HAPD+ n=15). Median indicated by black line. (B) Frequency of chronic activation marker expression (HLA-DR$^+$CD38$^+$) in T_{REG} and T_{CCR4-} reservoirs. Median indicated by black line. (C) log_{10}PVL (copies/10^6 cells) measured in matched T_{REG} and T_{CCR4-} reservoirs and bulk PBMCs, each coloured dot represents a different donor. (D) Frequency of T_{REG} and T_{CCR4-} cells as a percentage of CD4$^+$CD45RA$^-$ population in splenocytes from an HTLV-1c humanised mouse model at 2 weeks post infection (HTLV-1c- n=7, HTLV-1c+ n=8). Mean indicated by black line ± SD. (E) Frequency of chronic activation marker expression (HLA-DR$^+$CD38$^+$) in T_{REG} and T_{CCR4-} reservoirs of humanised mouse splenocytes. Mean indicated by black line ± SD. (F) log_{10}PVL (copies/10^6 cells) of paired T_{REG}, T_{CCR4-} and bulk splenocyte (spleno) reservoirs in 5 HTLV-1c+ humanised mice, each colour represents different mouse. Statistical significance was assessed in (A) and (B) using Kruskal-Wallis test with false discovery rate (FDR) adjustment, in (C) using Friedman test with FDR adjustment, in (D) and (E) using unpaired T-tests with FDR adjustment, and in (F) using RM one-way ANOVA with single pooled variance and FDR adjustment. *$p<0.05$, **$p<0.01$, ***$p<0.001$, ****$p<0.0001$.

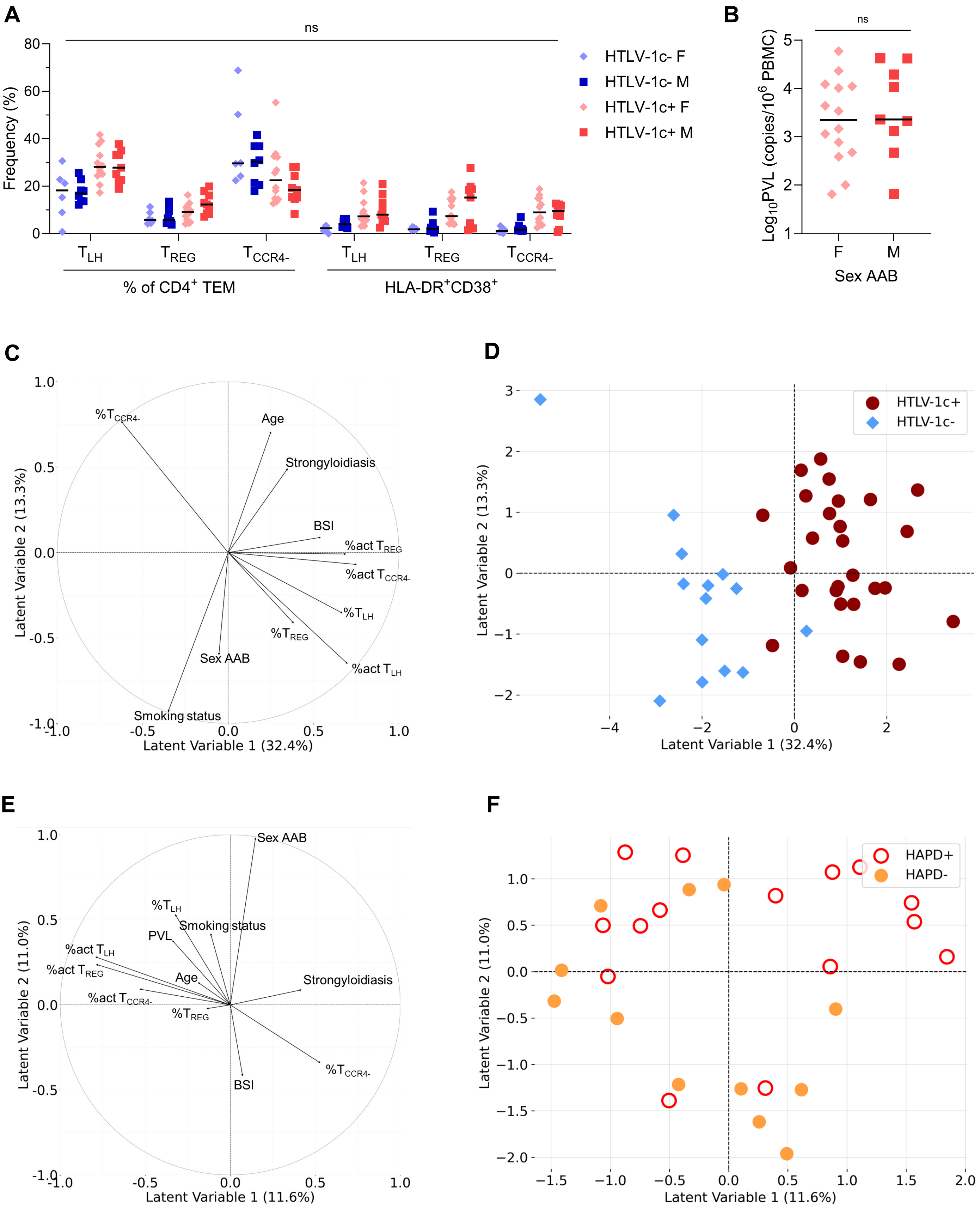

A
ns
Frequency (%)
HTLV-1c- F
HTLV-1c- M
HTLV-1c+ F
HTLV-1c+ M
TLH
TREG
TCCR4-
% of CD4+ TEM
HLA-DR+CD38+
B
ns
Log10PVL (copies/10^6 PBMC)
F
M
Sex AAB
C
%TCCR4-
Age
Strongyloidiasis
BSI
%act TREG
%act TCCR4-
%TLH
%TREG
%act TLH
Sex AAB
Smoking status
Latent Variable 1 (32.4%)
Latent Variable 2 (13.3%)
D
HTLV-1c+
HTLV-1c-
Latent Variable 1 (32.4%)
Latent Variable 2 (13.3%)
E
Sex AAB
%TLH
Smoking status
PVL
%act TLH
%act TREG
Age
Strongyloidiasis
%act TCCR4-
%TREG
%TCCR4-
BSI
Latent Variable 1 (11.6%)
Latent Variable 2 (11.0%)
F
HAPD+
HAPD-
Latent Variable 1 (11.6%)
Latent Variable 2 (11.0%)

Supplementary Figure 4: Controlling T-cell phenotype data for potential demographic and clinical confounders. (A) T-cell phenotype and activation frequencies for HTLV-1c- and HTLV-1c+ groups, stratified by sex assigned at birth (AAB). (B) Log10(PVL) (copies per 10^6 PBMCs) of HTLV-1c+ donors, stratified by sex AAB. (C) Partial least squares discriminant analysis (PLS-DA) latent variable loadings, for total cohort. (D) PLS-DA scores for latent variables 1 and 2, for all donors. HTLV-1c- donors are depicted with blue triangles, HTLV-1c+ donors are depicted with maroon circles. (E) PLS-DA latent variable loadings, for HTLV-1c+ subgroup. (F) PLS-DA scores for latent variables 1 and 2, for HTLV-1c+ donors. HAPD- donors are depicted with orange filled circles, HAPD+ donors are depicted with empty red circles. Statistical significance in (A) was assessed with multiple Mann-Whitney tests with false discovery rate (FDR) adjustment, and in (B) with Mann-Whitney test. Non-significant=ns, $p>0.05$.

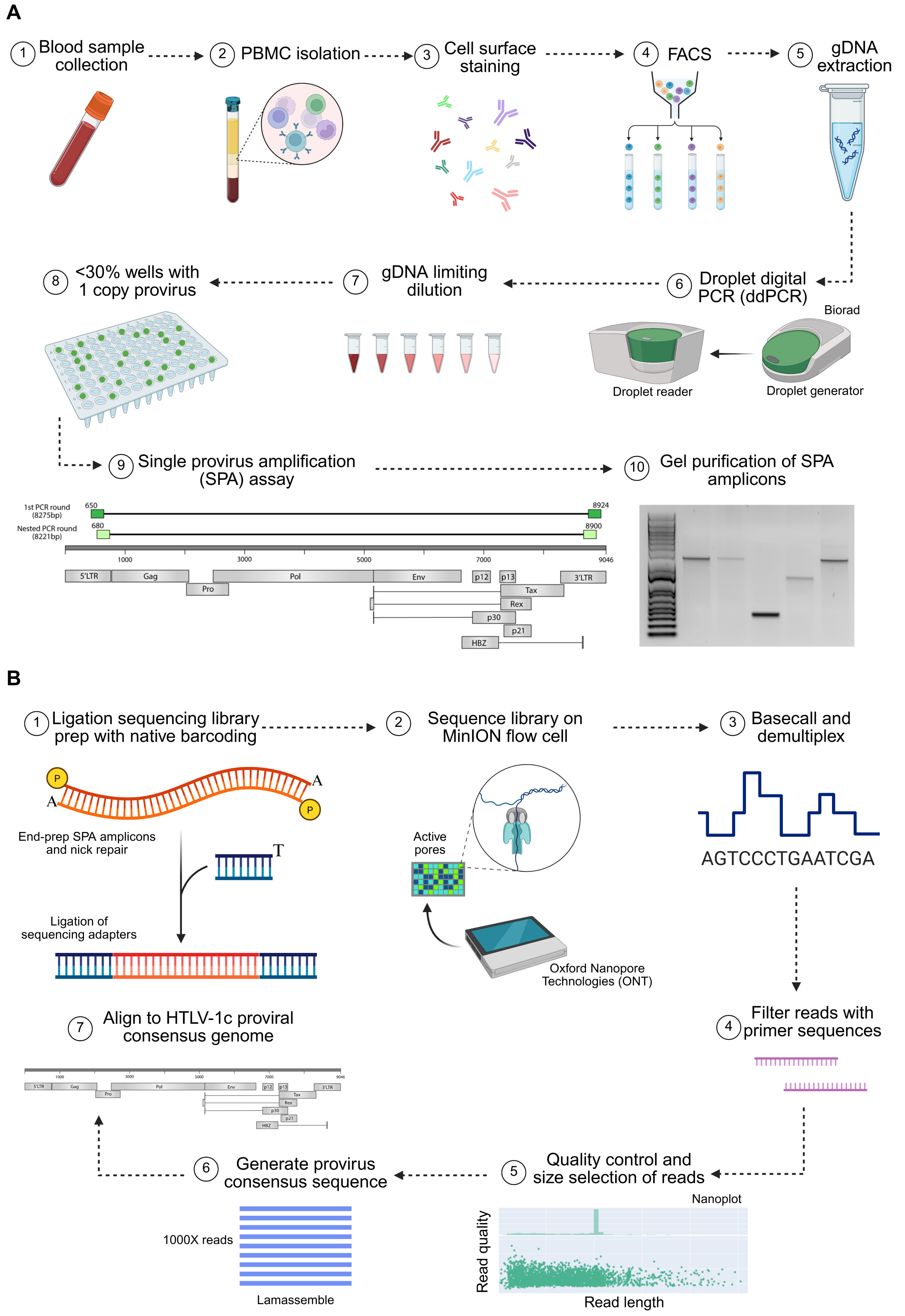

A
1 Blood sample collection
2 PBMC isolation
3 Cell surface staining
4 FACS
5 gDNA extraction
6 Droplet digital PCR (ddPCR)
Biorad
Droplet reader
Droplet generator
7 gDNA limiting dilution
8 <30% wells with 1 copy provirus
9 Single provirus amplification (SPA) assay
1st PCR round (8275bp)
Nested PCR round (8221bp)
650
8924
680
8900
1000
3000
5000
7000
9046
5'LTR
Gag
Pro
Pol
Env
p12
p13
3'LTR
Tax
Rex
p30
p21
HBZ
10 Gel purification of SPA amplicons
B
1 Ligation sequencing library prep with native barcoding
End-prep SPA amplicons and nick repair
Ligation of sequencing adapters
2 Sequence library on MinION flow cell
Active pores
Oxford Nanopore Technologies (ONT)
3 Basecall and demultiplex
AGTCCCTGAATCGA
4 Filter reads with primer sequences
5 Quality control and size selection of reads
Nanoplot
Read quality
Read length
6 Generate provirus consensus sequence
1000X reads
Lamassemble
7 Align to HTLV-1c proviral consensus genome

Supplementary Figure 5: Single provirus amplification and Oxford Nanopore Technologies sequencing (SPA-ONT-seq) design. (A) SPA assay workflow: 1) Whole blood collection at Alice Springs Hospital with written and verbal consent in primary language; 2) PBMC isolation by Ficoll-Paque centrifugation method; 3) PBMC cell surface staining with cocktail of anti-human antibodies; 4) Fluorescence activated cell sorting (FACS) of $CD4^{+}$ T-cell phenotypes; 5) genomic DNA (gDNA) extraction and purification; 6) Droplet digital PCR of gDNA to determine the proviral load (PVL), by quantifying the number of *tax* copies per genome, with *RPP30* used as the reference gene; 7) Limiting dilution of gDNA based on the PVL determined by ddPCR; 8) Less than 30% of wells with one copy of HTLV-1c provirus; 9) Single provirus amplification (SPA) assay using a nested touchdown PCR protocol with primers staggered in the LTRs to amplify near full-length genomes; 10) Agarose gel purification of provirus positive wells. (B) ONT-seq workflow: 1) Amplified provirus was prepared for sequencing by ligation of sequencing adapters and barcodes to enable multiplexing; 2) Pooled DNA libraries were sequenced on a MinION; 3) Basecalling of reads, demultiplexing of samples, and adapter trimming was performed using Guppy or Dorado and; 4) reads were filtered for the presence of the nested primer binding sequences; 5) Reads were size selected from the peak of the read-length distribution; 6) Filtered reads were used to generate a consensus sequence using the Lamassemble package; 7) Consensus sequences were then aligned to an Australian HTLV-1c consensus sequence. Figure made with BioRender.

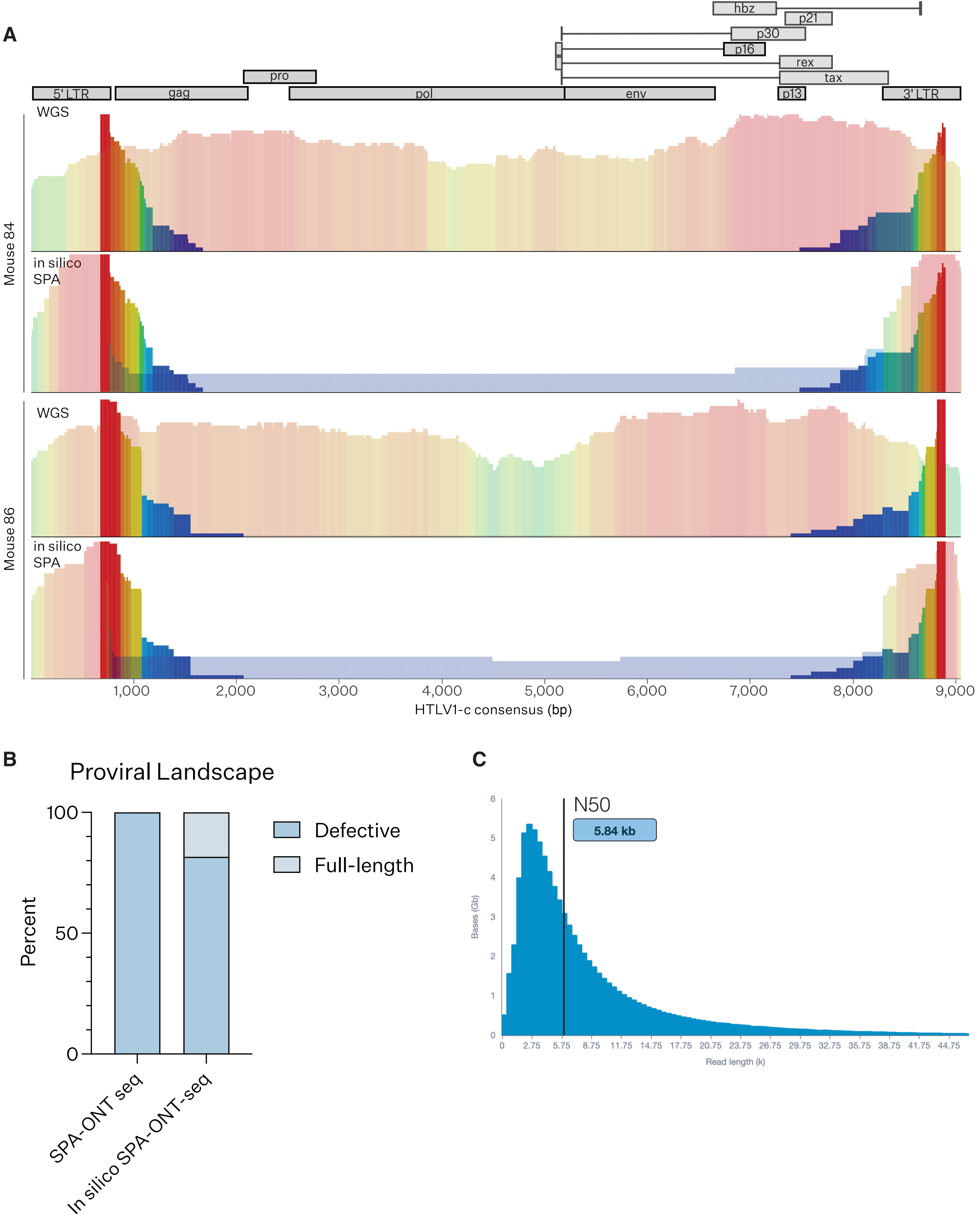

A
hbz
p21
p30
p16
rex
tax
pro
5' LTR
gag
pol
env
p13
3' LTR
WGS
in silico
SPA
Mouse 84
WGS
in silico
SPA
Mouse 86
1,000
2,000
3,000
4,000
5,000
6,000
7,000
8,000
9,000
HTLV1-c consensus (bp)
B
Proviral Landscape
100
50
0
Percent
Defective
Full-length
SPA-ONT seq
In silico SPA-ONT-seq
C
N50
5.84 kb
Bases (Gb)
0
1
2
3
4
5
6
0
2.75
5.75
8.75
11.75
14.75
17.75
20.75
23.75
26.75
29.75
32.75
35.75
38.75
41.75
44.75
Read length (k)

Supplementary Figure 6: Validation of SPA-ONT-seq strategy. (A) Long-read whole genome sequencing (WGS) was performed alongside SPA-ONT-seq on DNA extracted from splenocytes of two HTLV-1c+ hu-NSG mice at 6 weeks post infection. Reads were mapped to a previously published HTLV-1c consensus sequence, and quantified in a 1bp sliding window along the length of the provirus (1st and 3rd panel, hot-cold gradient: transparent represents quantification of reads from WGS; opaque overlay represents quantification of reads from SPA-ONT-seq). We performed an in-silico SPA assay, but filtering WGS reads possessing both forward and revers SPA primer binding sequencing within the LTRs (2ndand 4th panel, hot-cold gradient: transparent represents quantification of reads from in silico SPA-seq; opaque overlay represents quantification of reads from SPA-ONT-seq). (B) Bar graph showing percentage of HTLV-1c structural variants detected by in silico SPA in long-read WGS (40 genomes, n=2) and those detected by SPA-ONT-seq (72 genomes, n=2). (C) Read-length distribution of all reads sequenced by long-read whole genome sequencing of DNA used in SPA-ONT-seq in hu-NSG mice.

A

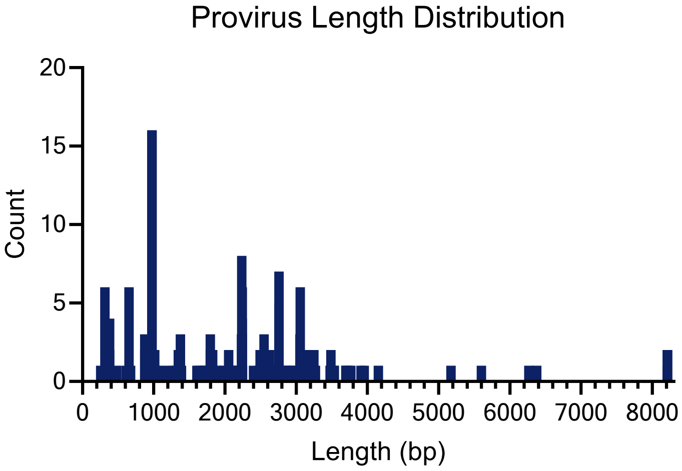
Provirus Length Distribution
Count
20
15
10
5
0
0
1000
2000
3000
4000
5000
6000
7000
8000
Length (bp)

B

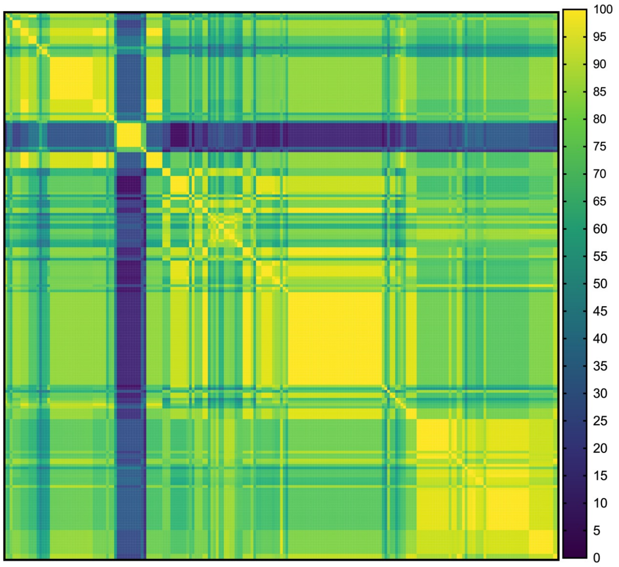
100
95
90
85
80
75
70
65
60
55
50
45
40
35
30
25
20
15
10
5
0

C

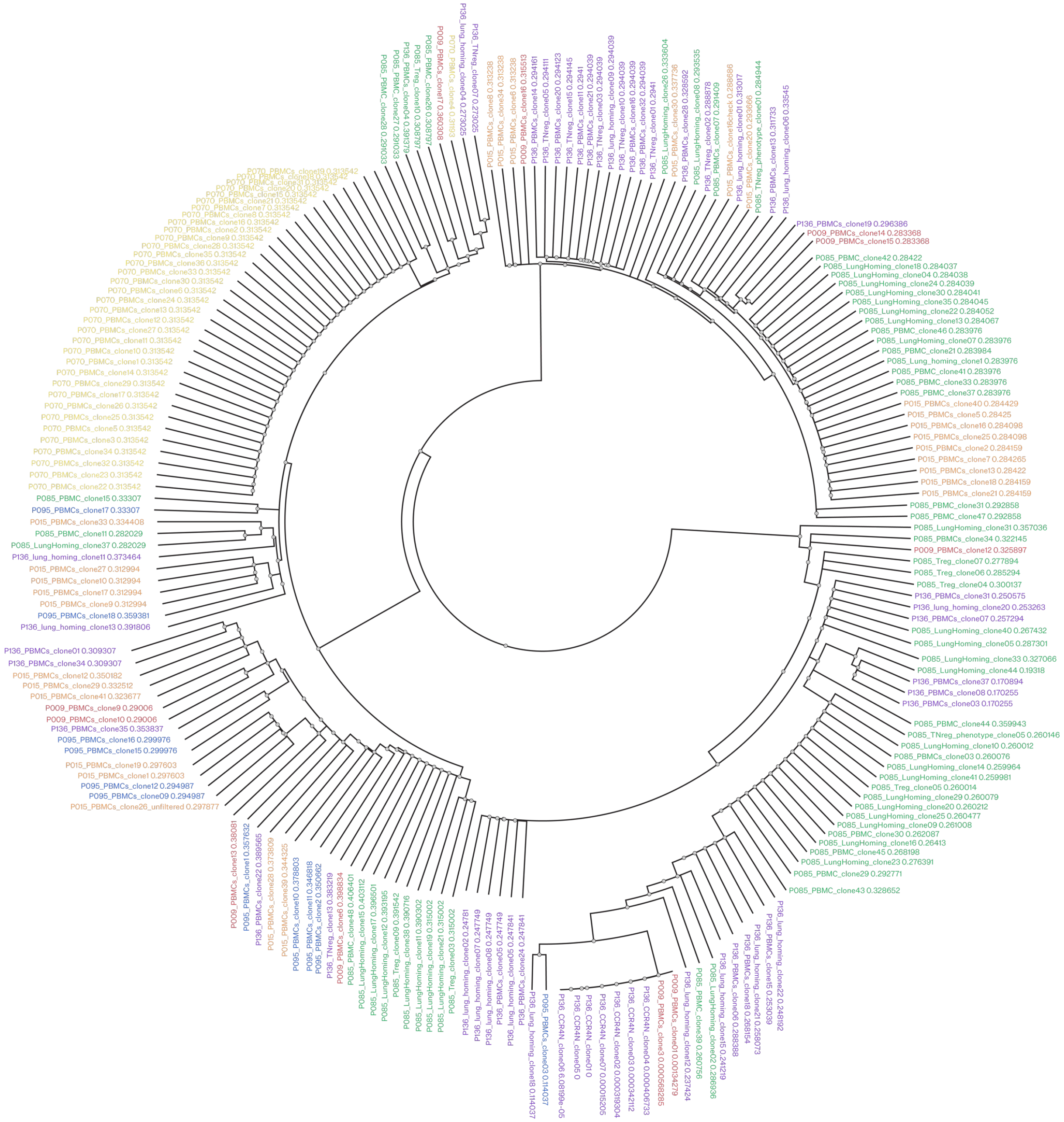

Supplementary Figure 7: SPA-ONT-seq output. (A) Assembled provirus length distribution for all 260 HTLV-1c proviruses assembled by SPA-ONT-seq from human donors. (B) Pairwise identity matrix generated by Clustal Omega for 260 provirus sequences assembled by SPA-ONT-seq in human donors. (C) Distance tree generated by Clustal Omega coloured by donor, used to calculated pairwise identify matrix in (B).

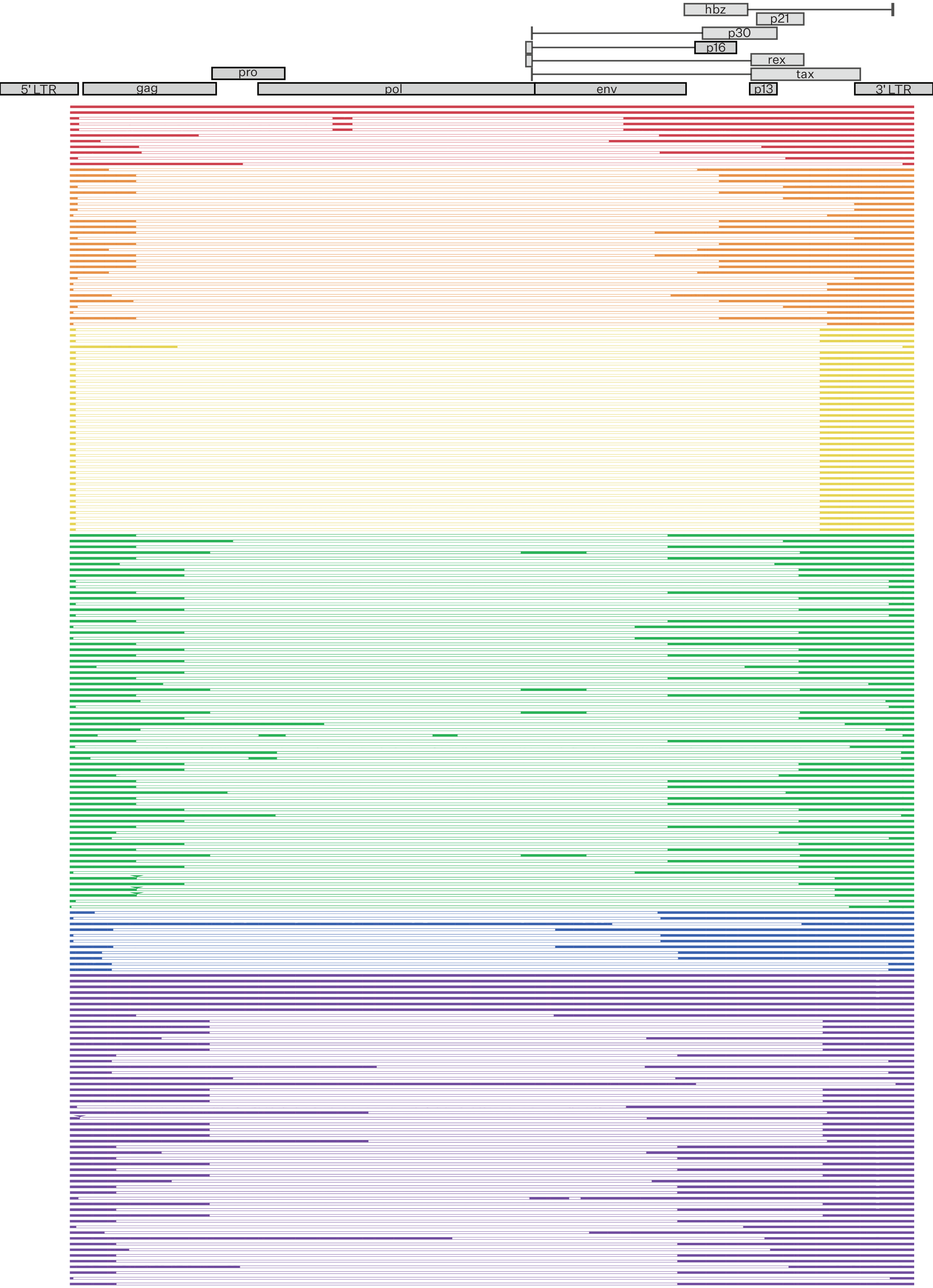
hbz
p21
p30
p16
rex
pro
tax
5' LTR
gag
pol
env
p13
3' LTR

Supplementary Figure 8: Multiple sequence alignment of consensus HTLV-1c provirus sequences assembled by SPA-ONT-seq. Alignment of 260 HTLV-1c provirus sequences, assembled from 6 participants, and aligned to the HTLV-1c consensus sequence21. Sequences coloured by donor (P009 in red, P0015 in orange, P070 in yellow, P085 in green, P095 in blue, P136 in purple) in ascending order.

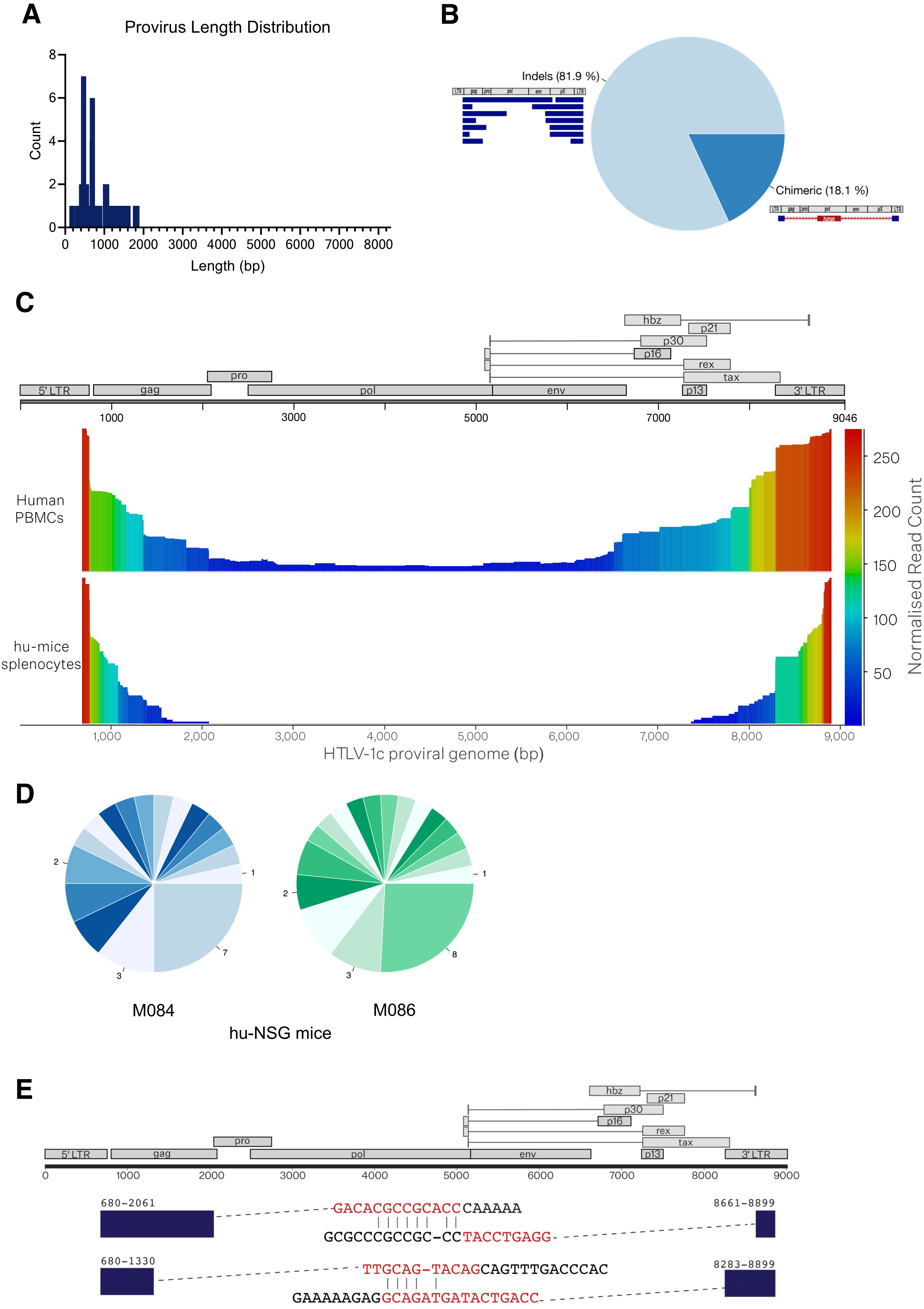
A
Provirus Length Distribution
Count
Length (bp)
0 1000 2000 3000 4000 5000 6000 7000 8000
B
Indels (81.9 %)
Chimeric (18.1 %)
LTR gag pro pol env pX LTR
human
C
hbz
p21
p30
p16
rex
tax
pro
5' LTR
gag
pol
env
p13
3' LTR
1000 3000 5000 7000 9046
Human PBMCs
hu-mice splenocytes
250 200 150 100 50
Normalised Read Count
1,000 2,000 3,000 4,000 5,000 6,000 7,000 8,000 9,000
HTLV-1c proviral genome (bp)
D
M084
M086
hu-NSG mice
E
0 1000 2000 3000 4000 5000 6000 7000 8000 9000
680–2061
8661–8899
GACACGCCGCACCCAAAAA
GCGCCCGCCGC-CCTACCTGAGG
680–1330
8283–8899
TTGCAG-TACAGCAGTTTGACCCAC
GAAAAAGAGGCAGATGATACTGACC

Supplementary Figure 9: The HTLV-1c proviral landscape in a humanised mouse model of infection. (A) Assembled provirus length distribution for all 72 HTLV-1c proviruses assembled by SPA-ONT-seq from humanised mice. (B) Distribution of HTLV-1c structural variants of 72 proviral genomes assembled by SPA-ONT-seq from 2 HTLV-1c+ hu-NSG mice, shown with a schematic representation of each variant detected. (C) Normalised coverage of each nucleotide of proviral genomes enriched from PBMCs in human donors (top, n=6) and hu-NSG mice (bottom, n=2) (hot-cold gradient), aligned to the HTLV-1c consensus genome. (D) Quantification of unique breakpoints in 60 defective proviral genomes containing a large internal deletion originating from two hu-NSG mice (M084, M086). (E) Schematic of representative defective proviral genomes (blue) in humanised mice aligned to the HTLV-1c consensus, with nucleotide resolution of regions of microhomology observed at the breakpoints. Nucleotides from the assembled defective genomes are depicted in red, flanking sequences from the HTLV-1c consensus sequence that show homology are depicted in black, and nucleotide identity is indicated by a line between them.

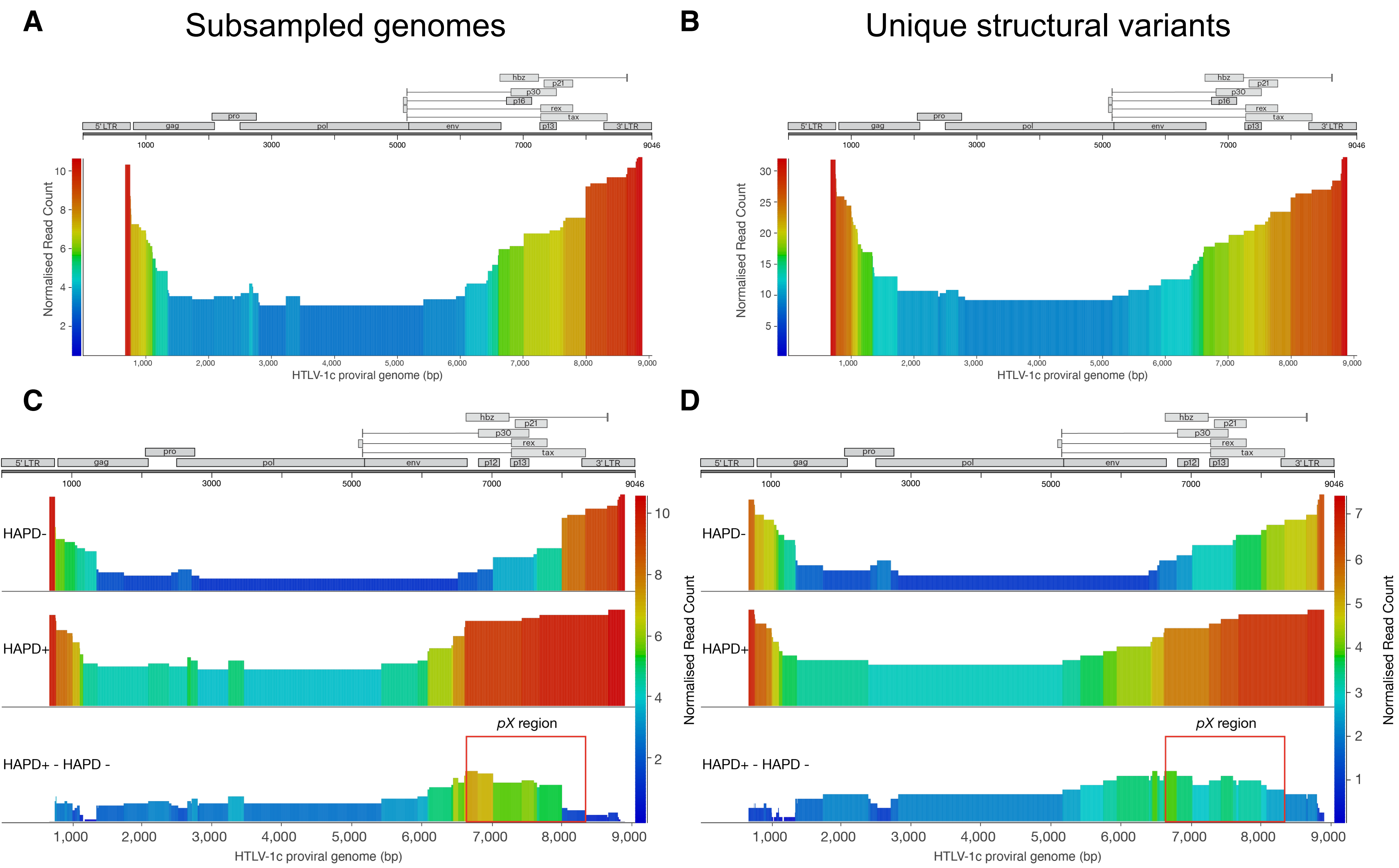

A
Subsampled genomes
B
Unique structural variants
hbz
p21
p30
p16
rex
tax
pro
5' LTR
gag
pol
env
p13
3' LTR
p12
1000
3000
5000
7000
9046
Normalised Read Count
HTLV-1c proviral genome (bp)
C
D
HAPD-
HAPD+
HAPD+ - HAPD -
pX region
1,000
2,000
3,000
4,000
5,000
6,000
7,000
8,000
9,000

Supplementary Figure 10: SPA-ONT-seq sensitivity analyses. Coverage of each nucleotide in the HTLV-1c proviral genome as presented in Figure 2A from (A) 66 contiguous proviral genomes subsampled equally from six HTLV-1c+ individuals (11 genomes per donor) aligned to the HTLV-1c consensus sequence (grey) and (B) 43 contiguous proviral genomes from six HTLV-1c+ individuals where haplotypes with unique breakpoint junctions have been aligned to the HTLV-1c consensus sequence (grey). Coverage was quantified at each nucleotide along the consensus, represented as counts that have been normalised to the largest library size along the length of the provirus (hot-cold gradient). (C) Normalised coverage of each nucleotide of 66 genomes subsampled equally from PBMCs of six HTLV-1c+ individuals (11 genomes per donor) (hot-cold gradient) stratified by HAPD– (top, n=3) or HAPD+ (middle, n=3), and difference (bottom), (D) and 43 proviral haplotypes with unique breakpoint junctions enriched from PBMCs.

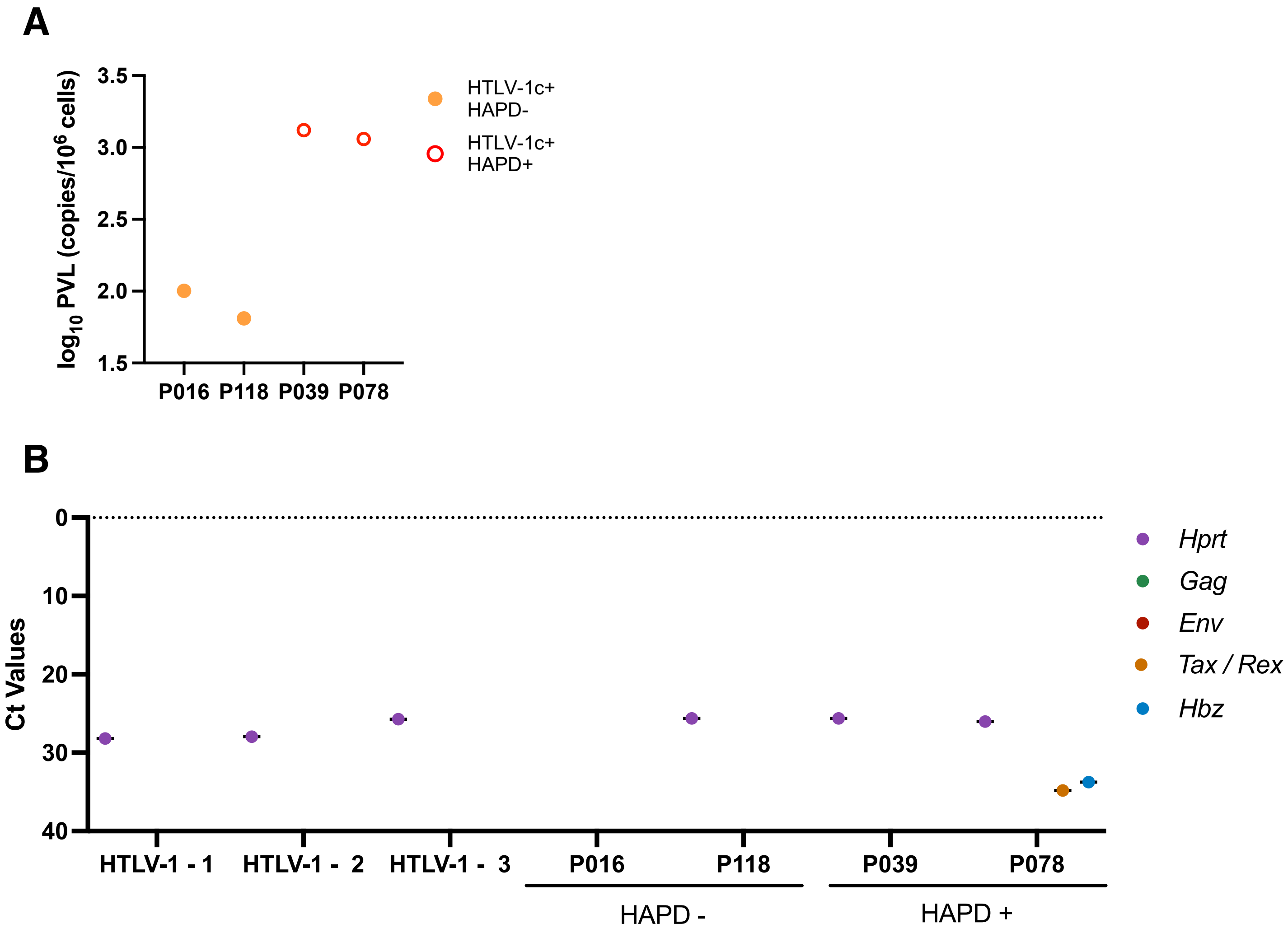

Supplementary Figure 11: *Hbz* mRNA expression in vivo and in vitro. (A) log10PVL of DNA in 4 HTLV-1c+ participants processed for RNA extraction and RT (B) Ct values of cellular housekeeping transcript HPRT (purple) and viral transcripts Gag (green), Env (Red), Tax/Rex (orange) and Hbz (blue) for in HTLV-1 - and HTLV-1 + HAPD – and HAPD + clinical samples detected by RT-qPCR. Values represent mean of technical triplicate.

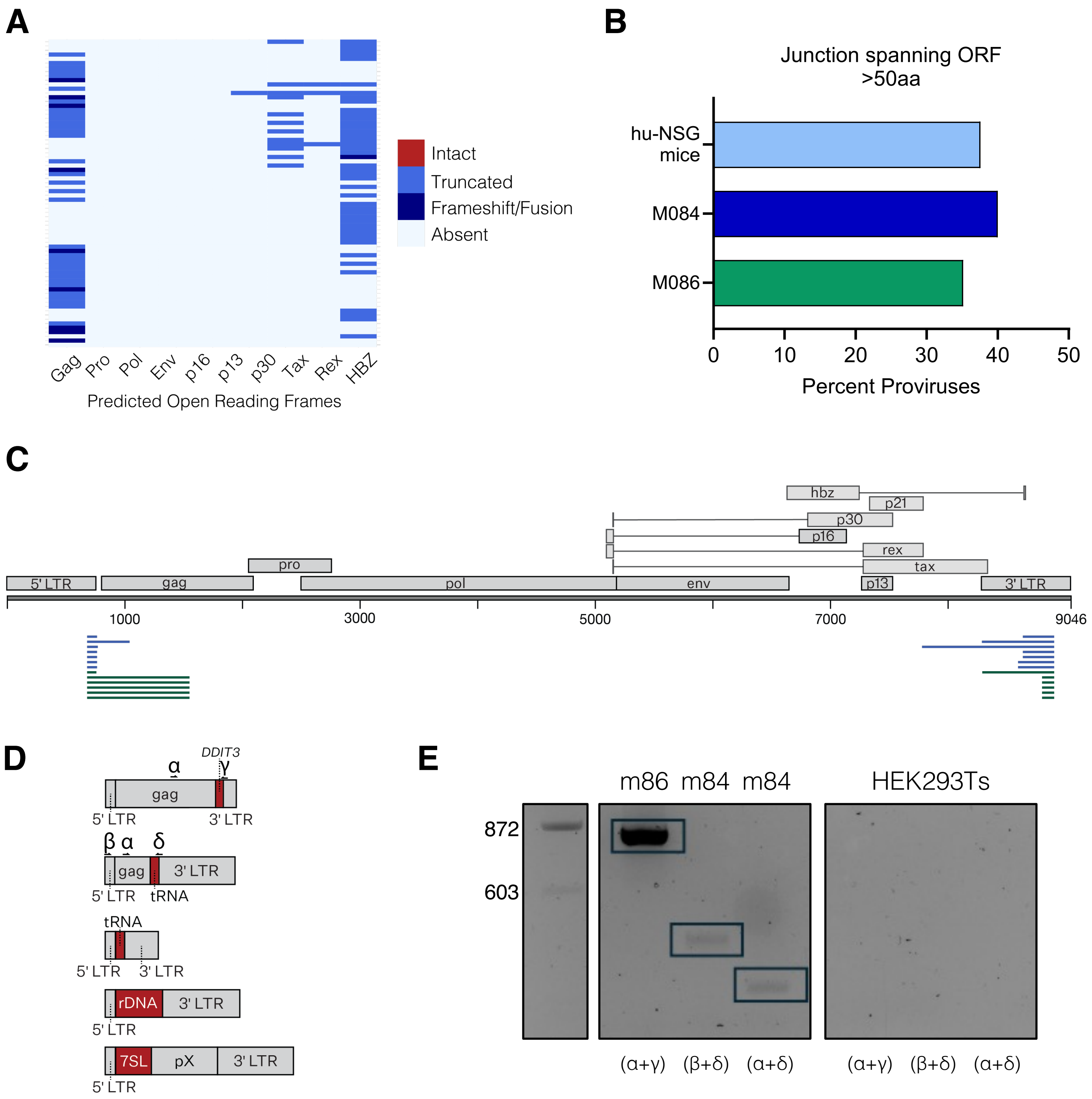
A
Intact
Truncated
Frameshift/Fusion
Absent
Gag
Pro
Pol
Env
p16
p13
p30
Tax
Rex
HBZ
Predicted Open Reading Frames
B
Junction spanning ORF >50aa
hu-NSG mice
M084
M086
0
10
20
30
40
50
Percent Proviruses
C
hbz
p21
p30
p16
rex
tax
pro
5' LTR
gag
pol
env
p13
3' LTR
1000
3000
5000
7000
9046
D
DDIT3
α
γ
gag
5' LTR
3' LTR
β α δ
gag
3' LTR
5' LTR
tRNA
tRNA
5' LTR
3' LTR
rDNA
3' LTR
5' LTR
7SL
pX
3' LTR
5' LTR
E
m86 m84 m84
HEK293Ts
872
603
(α+γ) (β+δ) (α+δ)
(α+γ) (β+δ) (α+δ)

Supplementary Figure 12: Coding potential of defective and chimeric HTLV-1c proviruses from humanised mice. (A) Heatmap depicting coding potential of total proviral landscape in humanised mouse model of infection (hu-NSG mice) at 6 weeks post infection (wpi) (n=2) scored as intact, truncated (including N and C terminal truncations), frameshift or fusion proteins, or absent in the population. Each row represents an individual proviral genome, and each column represents an open reading frame. (B) Frequency of HTLV-1c proviruses detected by SPA-ONT-seq with a novel junction spanning ORF greater than 50 amino acids in length in the humanised mouse model of infection. Junctions include between 5' and 3' defective provirus segments, inversion segments, or chimera segments. Mean frequency of mice (n=2) shown in light blue, M084 shown in dark blue, M086 shown in green. (C) Multiple sequence alignment of the proviral segments of the HTLV-1c:cellular chimeric proviruses, to the HTLV-1c consensus sequence, coloured by mouse. (D) Schematics of representative HTLV-1c:cellular chimeric proviral genomes enriched from hu-NSG mice, with proviral segment shown in grey, and cellular segment in red. Symbols (α, β, γ and δ) represent the approximate position of primers used to PCR validate the chimeric proviruses. (E) PCR validation of HTLV-1c:CHOP and HTLV-1c:tRNA-Glu proviruses. The gel image on the left shows the junction spanning PCR results in DNA isolated from splenocytes of hu-NSG mouse 84 and hu-NSG mouse 86 infected with HTLV-1c, and the gel image on the right shows the junction spanning PCR results in DNA isolated from HEK293Ts as a negative control. Ladder band sizes are as indicated. The black boxes indicates on-target products, confirmed by ONT long-read sequencing.
